# Nanocatalyst-enabled amide bond formation: advances, mechanistic insights, and future perspectives

**DOI:** 10.1039/d6ra05527c

**Published:** 2026-08-06

**Authors:** Satyaranjan Behera, Braja N. Patra

**Affiliations:** a Centre of Excellence in Advanced Materials and Applications, Utkal University, Vani Vihar Bhubaneswar 751004 India; b Department of Chemistry, Utkal University Bhubaneswar 751004 India behera.satyaranjan4@gmail.com brajapatra@gmail.com

## Abstract

Amide bonds are among the most ubiquitous linkages in pharmaceuticals, agrochemicals, polymers, and natural products. Despite their importance, conventional methods for forming these linkages often suffer from drawbacks such as low atom economy, harsh reaction conditions, and the generation of considerable waste. In recent years, nanocatalyst-based approaches have gained increasing attention as a more efficient alternative. Their appeal lies in their unique physicochemical features, including large surface areas, tunable active sites, and adjustable electronic properties, all of which can enhance catalytic activity and selectivity. In this context, the present review examines recent progress in nanocatalytic strategies for amide bond formation. It brings together a variety of catalytic systems, ranging from metal and metal oxide nanoparticles to bimetallic and heterostructured materials, as well as single-atom catalysts and ligand-functionalized hybrid nanomaterials. Rather than listing developments alone, the discussion focuses on how these systems operate, with attention to mechanistic details and reaction pathways. Key transformations considered include the direct amidation of carboxylic acids, the coupling of amines with alcohols through dehydrogenation, decarbonylative processes involving acyl derivatives, and C–H activation routes. Challenges such as catalyst recovery, stability, and correlations between structure and performance are critically discussed. By articulating current advances alongside practical limitations, this review aims to guide future innovation toward greener, more efficient, and industrially viable nanocatalyst-assisted amide synthesis methodologies.

## Abstract 2

Nanocatalyst-enabled amide bond formation: an overview of reaction pathways, catalyst platforms and applications in pharmaceuticals, materials science, and polymer chemistry.

## Graphical abstract

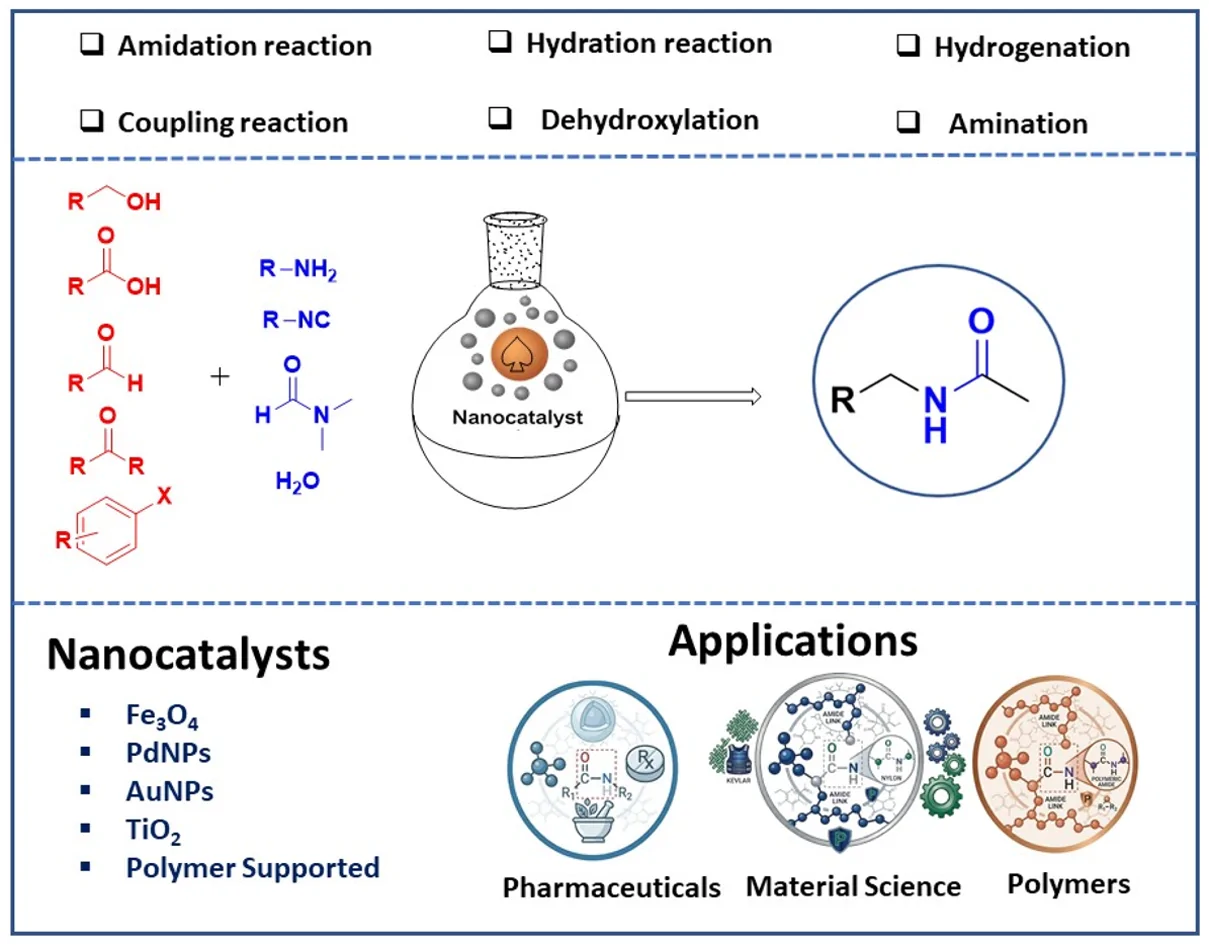

## 1. Introduction

Amides and their derivatives play a central role in organic synthesis and are widely utilized across industrial, biological, and pharmaceutical applications.^1–5^ This broad utility is largely due to their inherent stability, combined with a level of versatility that allows them to function across a wide range of chemical environments. The amide bond is particularly significant in biological systems, where it forms the backbone of peptides and proteins, thereby governing the structure and function of enzymes, hormones, and other essential biomolecules. Beyond natural systems, amides are frequently incorporated into synthetic compounds, including pharmaceuticals, agrochemicals, polymers, and advanced functional materials, where they contribute to molecular rigidity, stability, and predictable reactivity.^6–8^

**Fig. 1:**
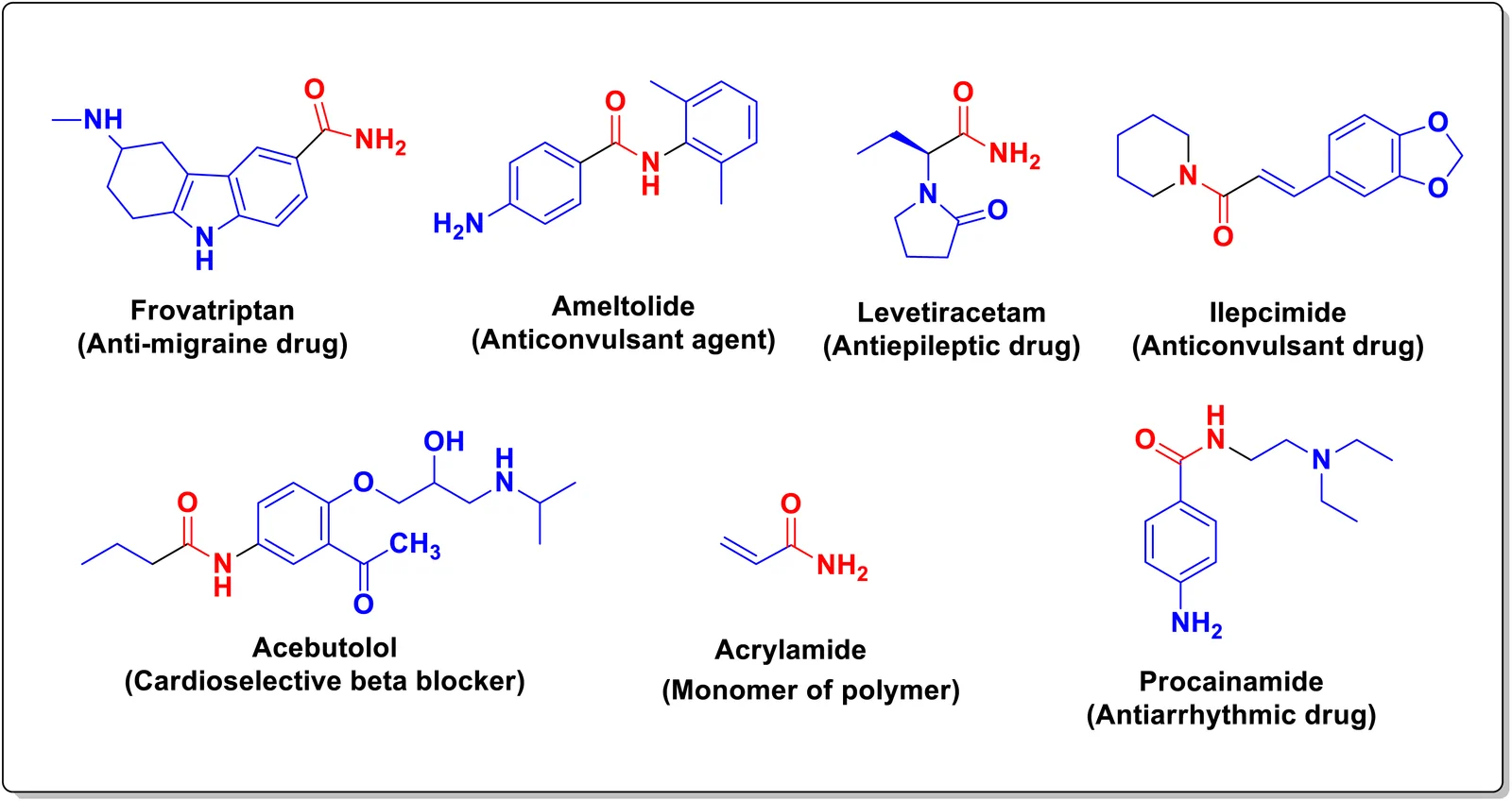
Representative useful amide compounds and their applications.

In medicinal chemistry, amides are widely regarded as key structural elements in many drug molecules. A large number of therapeutics including commonly used analgesics, antibiotics, and anticancer agents incorporate one or more amide functionalities that significantly affect their pharmacokinetic and pharmacodynamic properties^9–12^ (Fig. 1). One of the main advantages of the amide linkage is its relative resistance to hydrolysis under physiological conditions, which contributes to improved metabolic stability and often extends the effective lifetime of a drug. At the same time, the polarity and hydrogen bonding ability of amides can be adjusted to fine-tune solubility and interactions with biological targets. In addition, the design of peptidomimetics frequently relies on amide-based motifs to replicate the structural features of natural peptides, while enhancing bioavailability and resistance to enzymatic degradation.^13,14^

Amides are equally important from an industrial perspective. They are widely used as building blocks for high-performance polymers such as nylon and Kevlar, where extensive intermolecular hydrogen bonding is responsible for their notable mechanical strength, durability, and resistance to heat and chemical degradation. In the agrochemical sector, amide-containing compounds including many herbicides and insecticides benefit from the inherent stability and selectivity of the amide group, which helps sustain biological activity while minimizing unintended environmental effects.^15^ In addition, amides are frequently encountered in catalysis and coordination chemistry, and they participate in a variety of organic transformations, including hydrolysis, reduction, and rearrangement reactions, making them versatile intermediates in synthesis.^16–18^

**Fig. 2:**
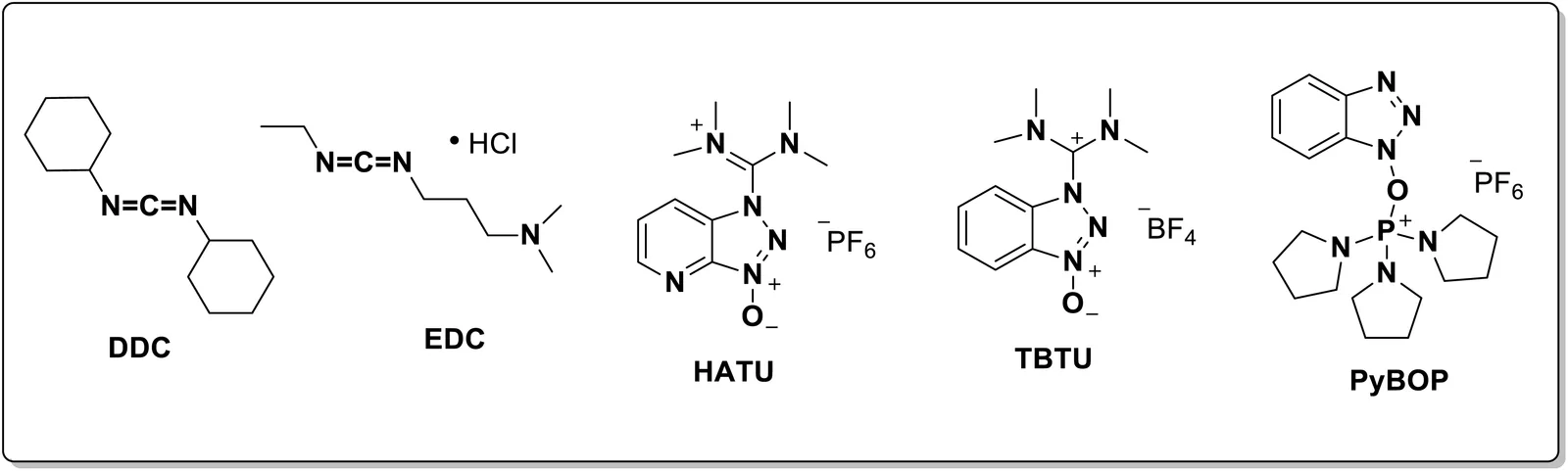
Traditionally used coupling agents for amide bond formation.

With the growing focus on sustainable and environmentally responsible chemistry, there has been increasing interest in more efficient strategies for amide bond formation. Recent developments methodologies such as catalytic amidation, enzymatic coupling, and the development of bio-derived amide containing polymers are gaining attention as they better align with green chemistry principles.^19,20^ However, many conventional methods for amide synthesis still rely on stoichiometric activation of carboxylic acids using reagents such as carbodiimides (DCC, EDC), acid chlorides, anhydrides, or coupling agents like HATU, TBTU, and PyBOP^16,21,22^ (Fig. 2). Although these methods are generally effective, they often generate significant amounts of waste, show limited atom economy, and may require harsh conditions or costly reagents, which can restrict their broader applicability, scalability, environmental sustainability.

Nanotechnology has opened new possibilities for improving amide synthesis by the use of nanocatalysts. Nanomaterial, typically below 100 nm in size, shows distinct physicochemical properties such as high surface area, increased surface reactivity, and tunable electronic features.^23–25^ Such characteristics make them well-suited for promoting a variety of organic transformations, including amide bond formation. In many cases, nanocatalysts outperform conventional systems by offering higher catalytic efficiency, shorter reaction times, and the ability to operate under milder conditions with improved selectivity. An additional practical advantages of a nanocatalyst is ease of recovery and reuse, which can help lower both cost and environmental impact.^26,27^

A broad range of nanocatalytic systems has been explored for this purpose, including metal nanoparticles (*e.g.*, Au, Ag, Pd, Cu), metal oxides (TiO_2_, ZnO, Fe_3_O_4_), carbon-based materials such as graphene oxide and carbon nanotubes, as well as hybrid nanostructures.^28–30^ Their catalytic behaviour is strongly influenced by factors such as particle size, morphology, surface modification, and the nature of the active sites. Mechanistically, nanocatalyst-mediated amidation generally involves activation of the carboxylic acid (or a related substrate), followed by nucleophilic attack by the amine. Depending on the system, this enhancement can arise from acid–base interactions, redox pathways, or surface-mediated adsorption and activation processes.

Overall, this review provides recent advances of nanocatalytic approaches for the synthesis of amides covering catalyst types, mechanistic insights, key synthetic strategies, representative applications and environmental aspects. By highlighting current challenges and emerging opportunities, it outlines pathways toward more efficient, selective, and sustainable amide-forming processes driven by nanocatalyst.

## 2. Nanocatalysts and its classification

Nanocatalysts are generally classified based on their composition, structure, and morphology. Each class exhibits distinct physicochemical properties that govern catalytic performance, including surface area, density of active sites, electronic structure, and overall stability. Ongoing advances in synthetic strategies, surface functionalization, and mechanistic insight are enabling the rational design of nanocatalysts with improved efficiency while also supporting the principles of green and sustainable chemistry.^31,32^

According to the phase relationship between the catalyst and the reactants, nanocatalysts are commonly classified as homogeneous and heterogeneous systems. In homogeneous nanocatalysis, catalytically active species such as single atom catalysts, molecular clusters or ultrasmall colloidal nanoparticles are homogeneously dispersed in the reaction medium, providing excellent contact with substrates and well-defined active centres often affording high catalytic activity and selectivity. Still, there are some major issues such as the isolation from the reaction mixture, recyclability and long-term stability, particularly for the large-scale applications.^33^

In contrast, heterogeneous nanocatalysts have active sites tethered to the surface of solid materials such as metal nanoparticles, metal oxides, carbon-based nanostructures, porous frameworks, and polymer-supported nanocomposites. Catalytic reactions occur mainly at the solid–liquid or solid–gas boundary, where the high surface-area-to-volume ratio of nanostructured materials maximizes the number of accessible active sites while facilitating catalyst recovery and reuse.^34^

The catalytic performance of heterogeneous nanocatalysts can be tailored by controlling properties such as particle size, morphology, surface acidity/basicity, electronic structure, and metal support interactions. These features enhance substrate activation, catalyst stability, and recyclability. In comparison, homogeneous nanocatalysts provide better molecular-level control and high chemoselectivity. Recognizing these differences provides a useful framework for understanding and selecting suitable nanocatalysts for efficient and sustainable amide bond formation.^35,36^

**Fig. 3:**
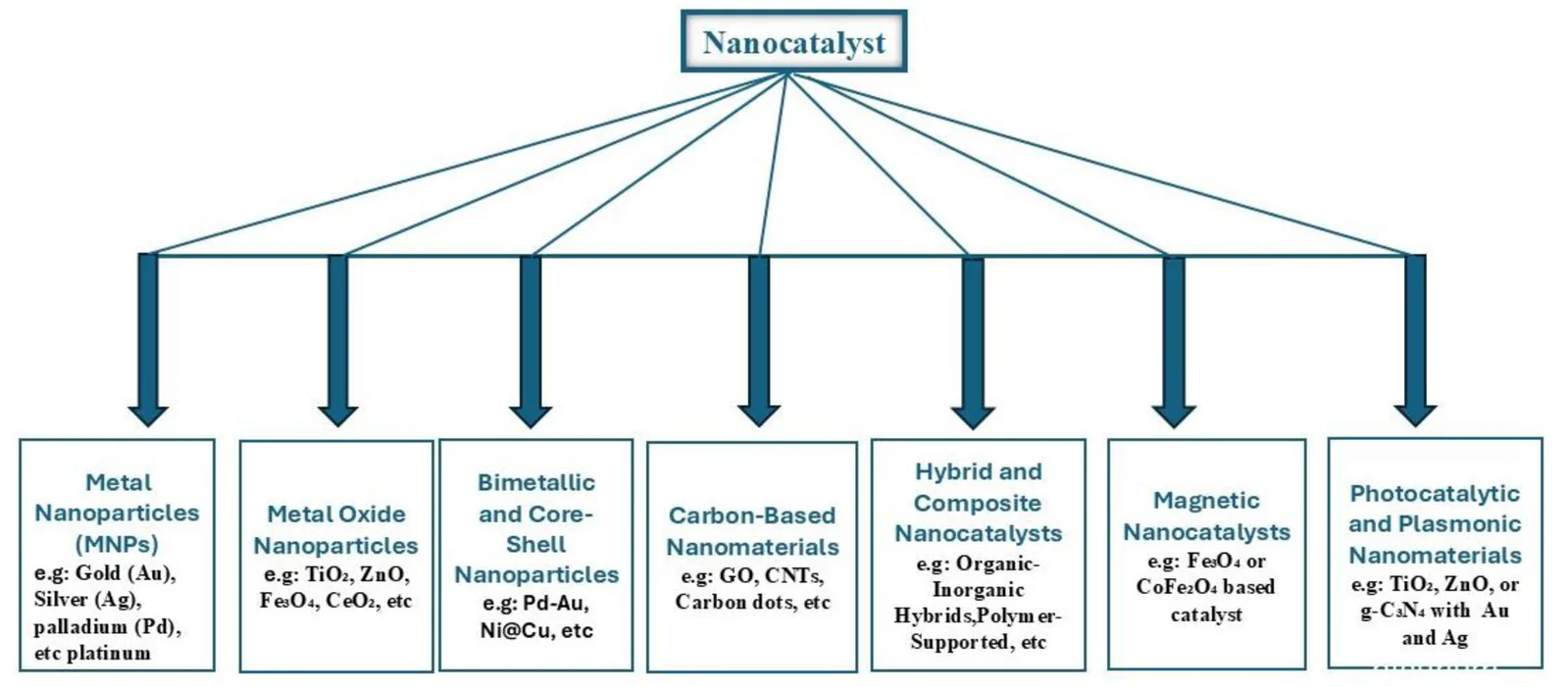
Classification of nanocatalyst for the synthesis of amides derivatives.

Nanocatalysts for amide synthesis, can be broadly classified by their structural and functional characteristics (Fig. 3). Metal nanoparticles such as Au, Pd, and Cu are particularly effective in oxidative amidation and cross-coupling processes. Metal oxides including TiO_2_, ZnO, and Fe_3_O_4_ offer versatile acid–base and redox properties, while bimetallic and core–shell systems often display enhanced catalytic activity due to synergistic effects.^37–39^ Carbon-based nanomaterials provide high surface area and excellent conductivity, making them suitable for a range of catalytic applications. Hybrid platforms such as MOFs and polymer-supported systems introduce additional functionality and structural flexibility.^40,41^ In addition, magnetic nanocatalysts facilitate simple separation and reuse, whereas photocatalytic and plasmonic nanomaterials enable light-driven, more sustainable pathways for amide bond formation.^42^

## 3. Synthetic strategies for amide formation

The most conventional strategy for amide bond formation involves the condensation of carboxylic acids with amines using activating or coupling reagents such as carbodiimides (DCC, EDC) or uronium-based reagents (HATU, HBTU)^21,22^ (Fig. 2). These reagents facilitate activation of the carboxylic acid toward nucleophilic attack by the amine, enabling amide formation under relatively mild conditions. Although extensively applied in peptide synthesis and medicinal chemistry, these approaches often generate stoichiometric byproducts, leading to significant waste and motivating the development of more sustainable alternatives.

Another well-established route is oxidative amidation, where alcohols or aldehydes are coupled with amines in the presence of a catalyst to afford amides. Transition-metal catalysts such as Ru, Rh, and Ag have been shown to promote the direct conversion of alcohols and amines into amides, with hydrogen gas as the only byproduct, making the process highly atom-efficient.^43,44^ This transformation generally proceeds through *in situ* oxidation of the alcohol to an aldehyde, followed by hemiaminal formation and subsequent oxidation to the amide.

In addition, ester aminolysis and transamidation reactions provide another important strategy for amide bond synthesis, where esters or pre-existing amides undergo nucleophilic substitution with amines to form new amide linkages. These processes can be catalyzed by metals, bases, or heterogeneous nanocatalysts and are particularly useful for late-stage functionalization of complex molecules. In recent years, nanocatalyst-enabled methodologies have gained significant attention, as they often allow these transformations to proceed under milder and more sustainable conditions, frequently employing air or molecular oxygen as environmentally benign oxidants.

## 4. Nanocatalysts in specific amide synthesis methodologies

Nanocatalysts have emerged as efficient and sustainable platforms for amide bond formation, offering high activity, selectivity, and recyclability under mild conditions. Their unique surface properties facilitate diverse transformations, including direct amidation, oxidative amidation, transamidation, and more methods. The following methodologies are described below.

### 4.1 Direct amidation of carboxylic acids with amines

**Scheme 1:**
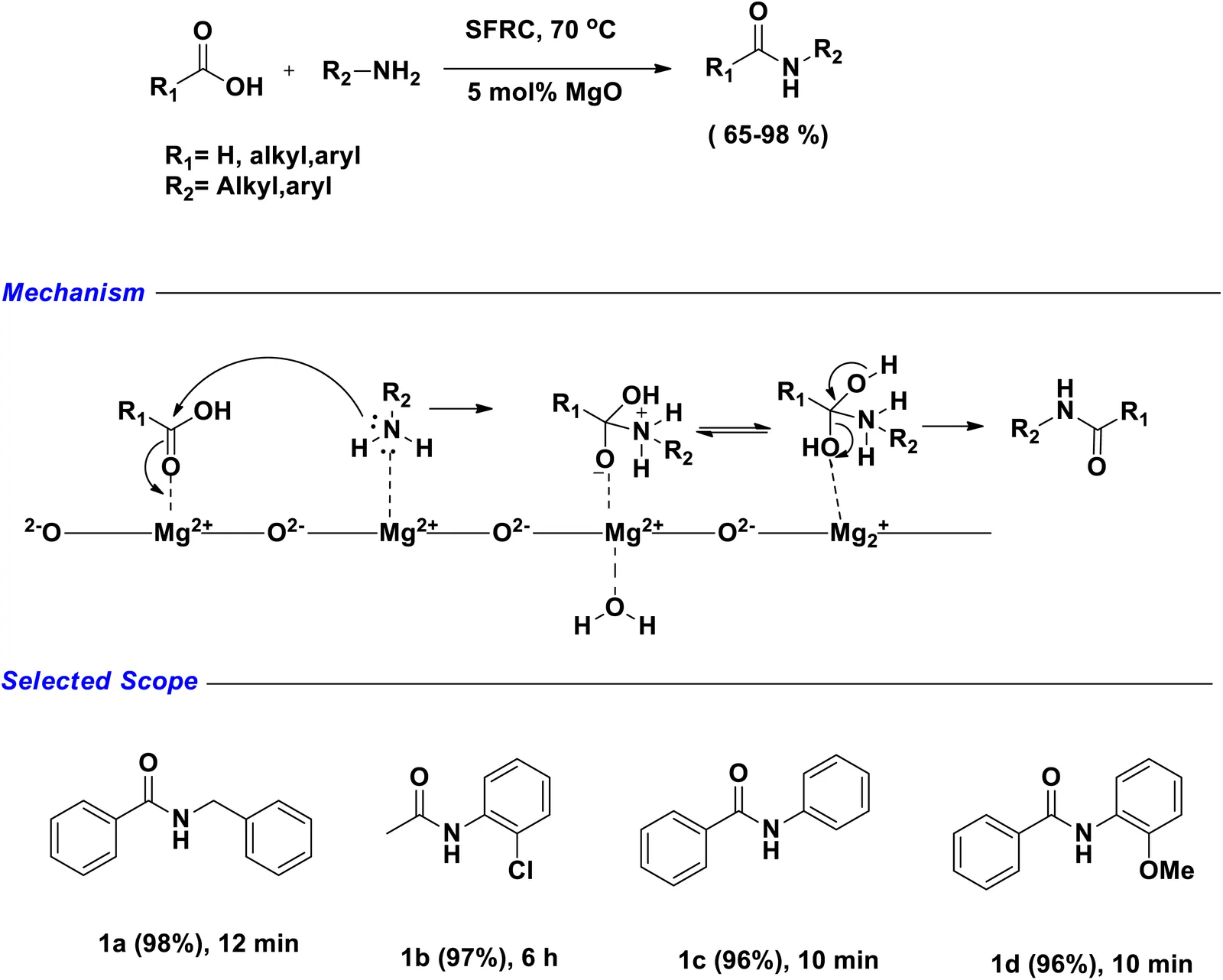
Direct amidation of carboxylic acid with amines using nano MgO catalyst.

In the year 2013 Thakur *et al.* reported a reusable magnesium oxide in nanoform (nano MgO) and used it as a catalyst for the solvent-free synthesis of amides.^45^ The group explored the scope of amide synthesis using various aromatic, heterocyclic, and aliphatic amines with carboxylic acids, with 5 mol% catalyst under solvent-free conditions, yielding excellent product yields (Scheme 1). Electron-rich anilines generally yielded higher yields than electron-poor anilines. Similarly, benzoic acids with electron-donating groups performed better than those with electron-withdrawing groups. Benzylamine and aliphatic amines yielded good to excellent results with several aromatic and benzoic acids. Mixed-substituted anilines (electron-donating and withdrawing) reacted well with acetic acid, forming acetamide derivatives efficiently. An unexpected cyclization reaction was observed when *o*-phenylenediamine was treated with acetic acid, yielding 2-methylbenzimidazole.

**Scheme 2:**
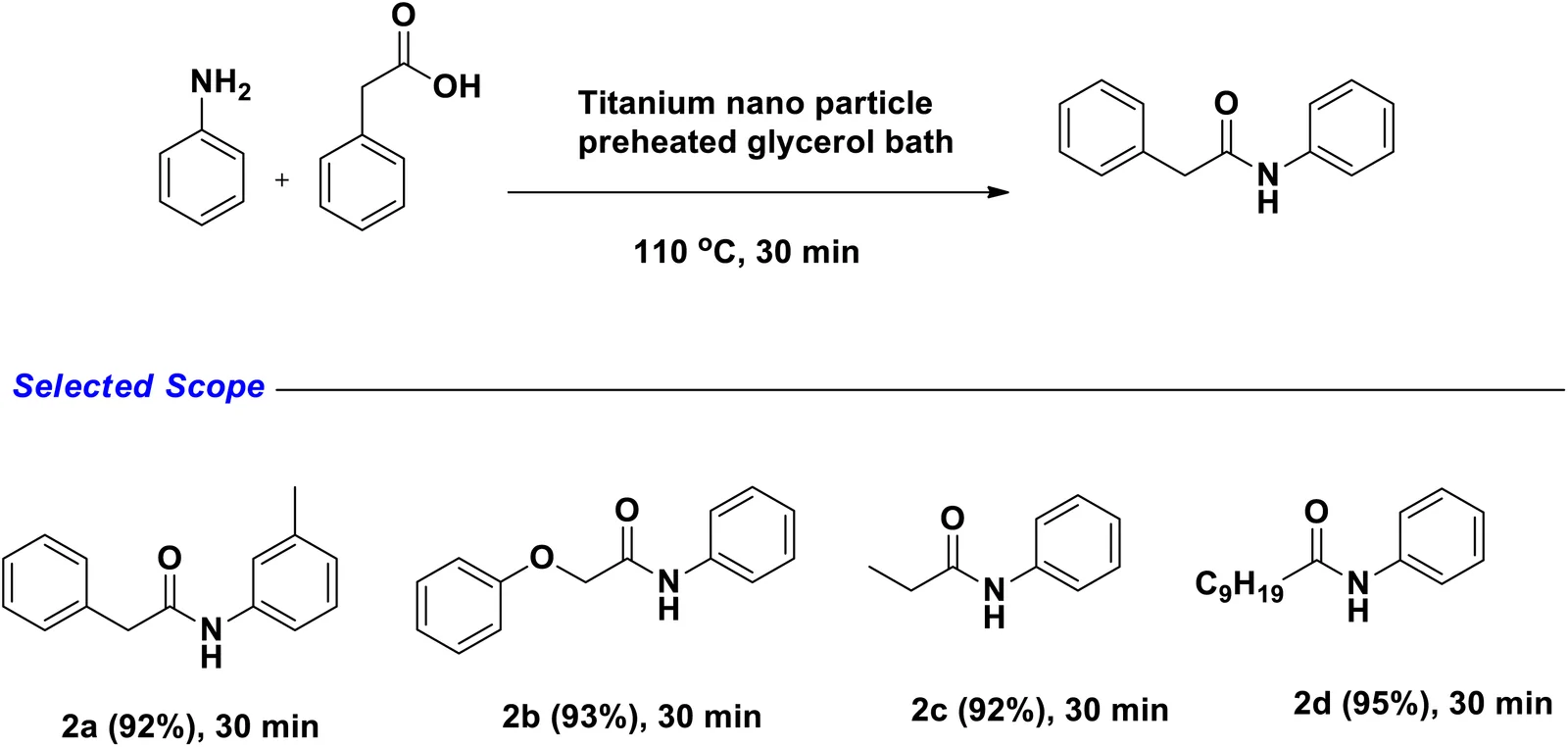
Direct amidation of aromatic and aliphatic carboxylic acids with aromatic amines by nano titanium catalyst.

The hydrothermal sol–gel technique was used for the synthesis of titania nanostructures with a uniform size reported by Gnana Kumar and his group.^46^ The same material was used for direct amidation of aromatic and aliphatic carboxylic acids with aromatic amines. Among the materials studied, sulfated titania nanotubes with the anatase phase exhibited excellent catalytic activity. In the optimization reaction, 24 wt% of the catalyst, at 110 °C temperature, within 30 min of time under solvent-free conditions, to provide excellent yields (75–98%) (Scheme 2).

**Scheme 3:**
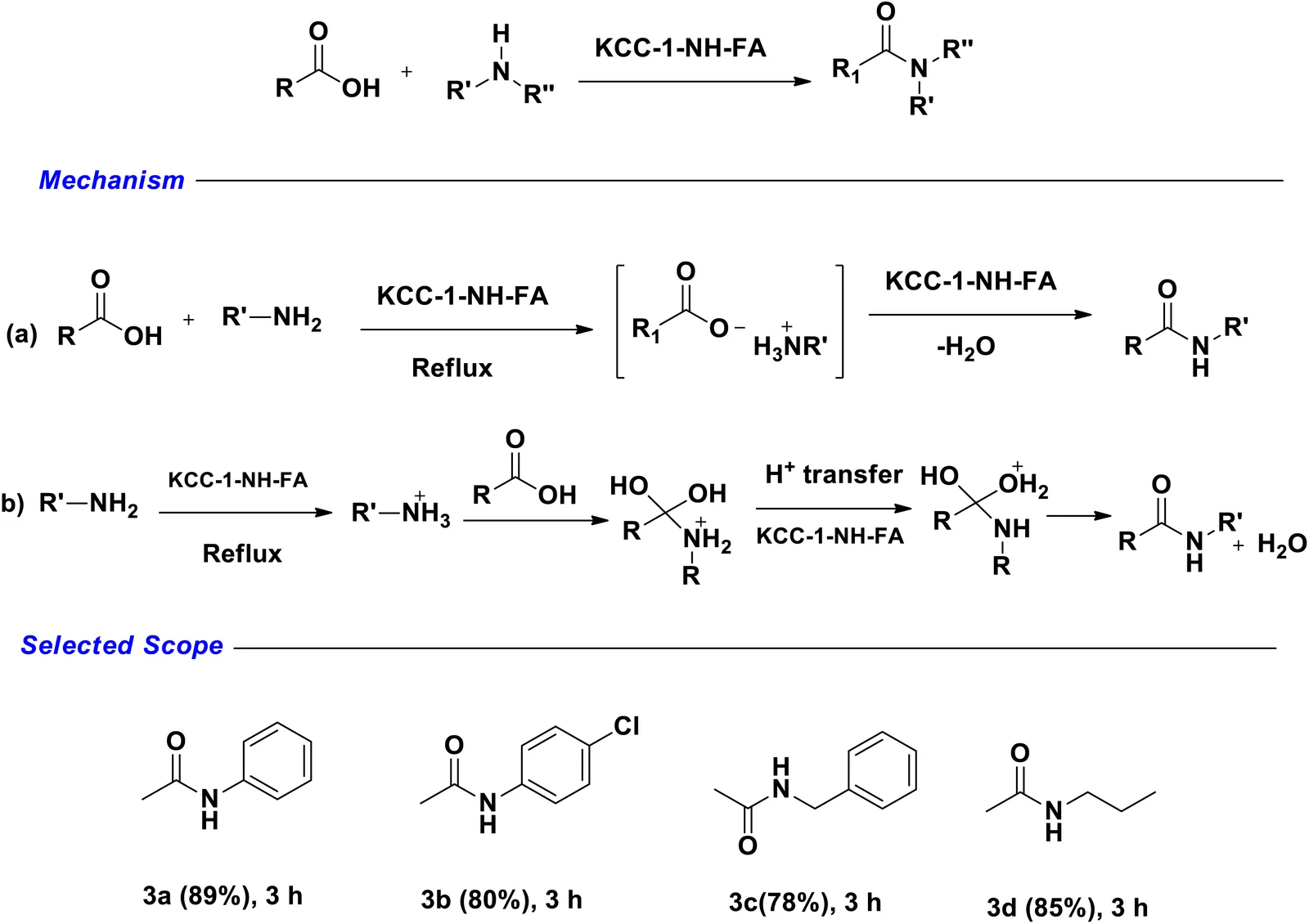
KCC-1-NH-FA catalyzed direct amidation of carboxylic acids with amines. Proposed mechanism Pathways: (a) ammonium carboxylate pathway and (b) tetrahedral intermediate pathway.

Shadjou and group developed and synthesized folic acid functionalized dendritic fibrous nano silica for direct amidation of carboxylic acids with amines, refluxed in toluene with 20 mg of optimized catalyst and provided excellent yields of the product.^47^ With the optimized reaction conditions the reaction of aliphatic, hetero aromatic, cyclic, and linear aliphatic amines with aliphatic carboxylic acid yields the desired amides with 74–89% yields with in short period of time (3–5 h) (Scheme 3). The catalyst can be regenerated and reused for several times.

### 4.2 Oxidative amidation of aldehydes

**Scheme 4:**
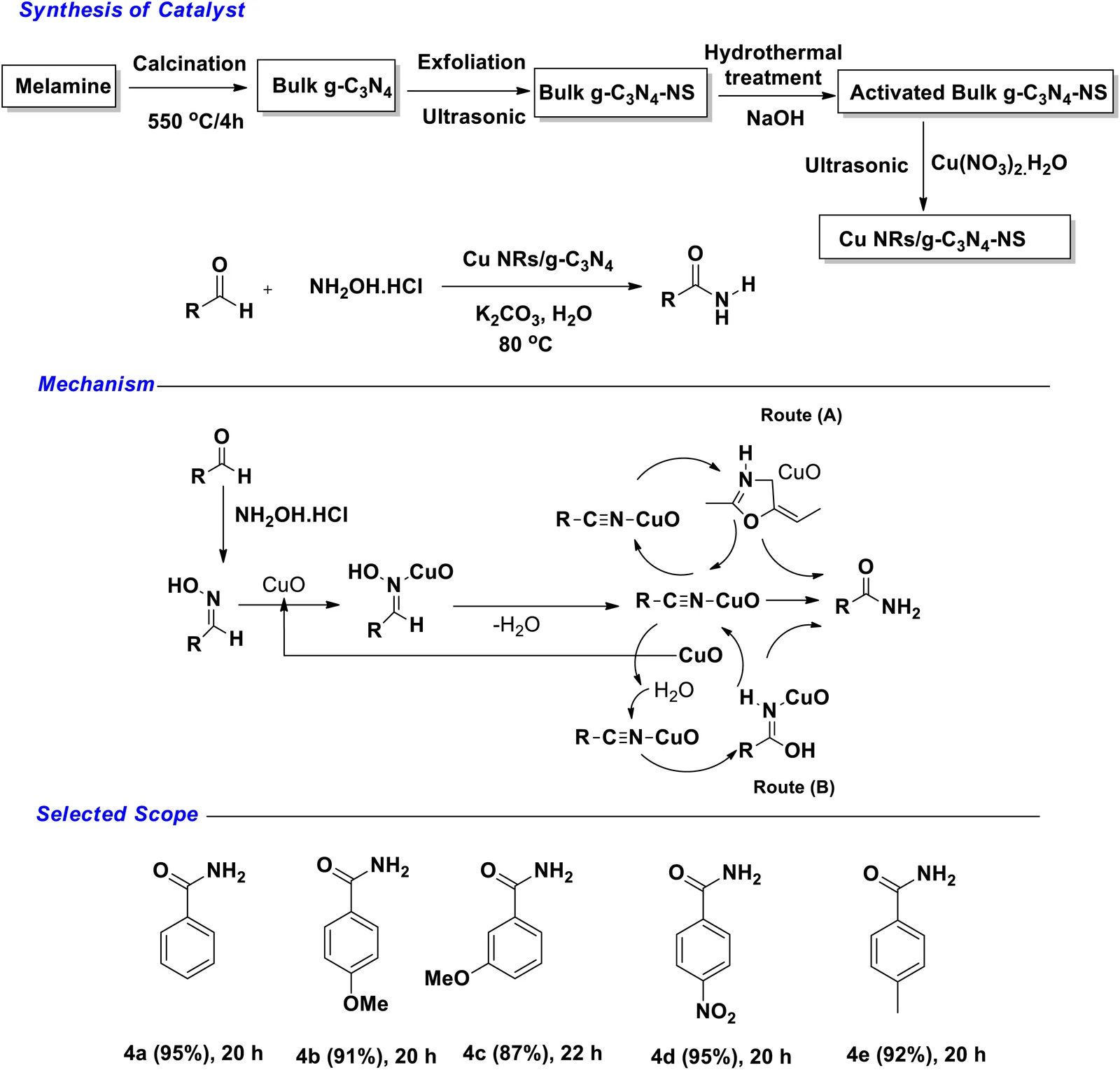
CuO/g-C_3_N_4_-NS catalyzed oxidative amidation of aldehydes with hydroxyl amines in water.

Shokouhimehr and co-workers developed CuO nanorods decorated with g-C_3_N_4_ nanosheets (CuO/g-C_3_N_4_-NS). They used CuO/g-C_3_N_4_-NS as an excellent colloidal nanocatalyst to synthesize amides from aldehydes and hydroxyl amines in water at 80 °C, using K_2_CO_3_ as the base. In this methodology, 10 mol% of the catalyst was sufficient to catalyse the reaction and provided good to excellent product yields^48^ (Scheme 4). High yields were achieved for all the targeted primary amides. The presence of either electron-donating or electron-withdrawing groups on the aldehydes resulted in excellent yields, suggesting that electronic effects do not significantly influence the reaction. Notably, electron-donating groups on the aromatic ring produced yields ranging from 77% to 92%, while electron-withdrawing groups on the ring yielded between 43% and 95%.

**Scheme 5:**
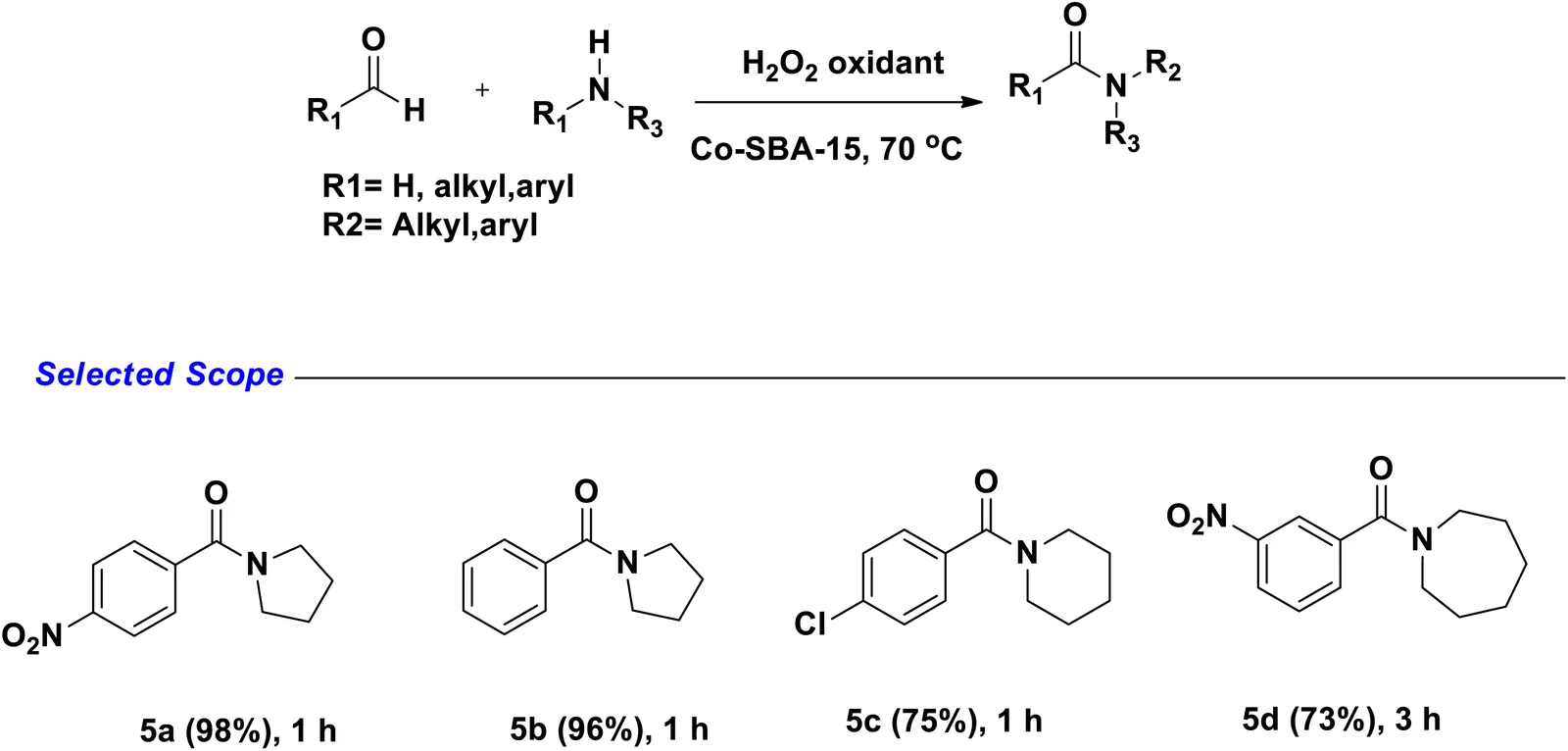
Oxidative amidation of aldehydes with amines using cobalt oxide nanoparticles supported (Co_3_O_4_/SiO_2_) catalyst.

Rajabi and group reported cobalt oxide nanoparticles supported silica (Co_3_O_4_/SiO_2_), used as efficient heterogeneous catalysts for the oxidative amidation of aldehydes with amines under solvent-free conditions.^49^ Varieties of aldehydes with amines were explored using 0.5 mol% of catalyst at 70 °C within 1–5 h in the presence of hydrogen peroxide as an oxidant, affording good to excellent yield (up to 98%) (Scheme 5). The reaction showed strong tolerance toward both electron-withdrawing and electron-donating groups on aromatic aldehydes, with NO_2_- and Cl-substituted substrates giving higher yields. The position of the nitro group had little effect, though 2-nitrobenzaldehyde gave moderate yield. Aliphatic aldehydes were also efficiently converted to amides in good yields.

**Scheme 6:**
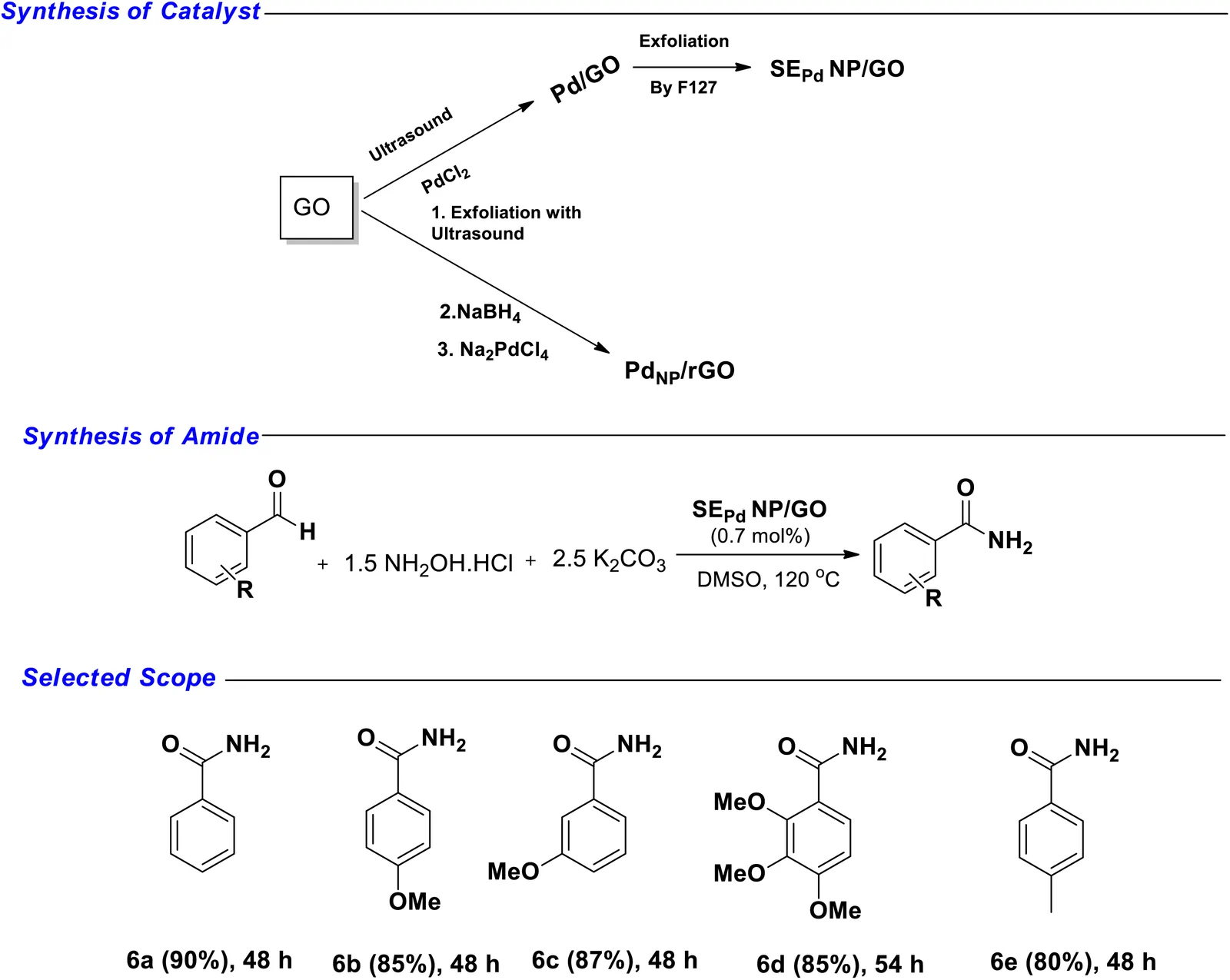
Oxidative amidation using Pd(nanoparticle)/GO catalyst.

Zeynizadeh *et al.* reported Pd nanoparticle-supported graphene oxide (Pd/GO) system, representing one of the first synthesized Pd-based heterogeneous nanocatalyst for amide synthesis.^50^ This catalyst was effectively employed in a one-pot cascade transformation, where aromatic aldehydes react with hydroxylamine hydrochloride (NH_2_OH HCl), initially forming oximes followed by an *in situ* Beckmann rearrangement to yield primary amides. During the catalytic process, the graphene oxide sheets underwent significant exfoliation in the presence of different polyethers, leading to the formation of highly dispersed and active catalytic sites. Among the polyethers tested, F127 demonstrated superior performance, both in enhancing exfoliation and improving overall catalytic efficiency. The developed protocol offers a greener and recyclable approach for amide synthesis, showing clear advantages over conventional stepwise methods due to its cascade nature. Typically, substituted benzaldehydes react with NH_2_OH HCl in the presence of K_2_CO_3_ as a base, using a small amount of water (0.5 mL) and DMSO (1.5 mL) as solvent, along with SEF127-stabilized Pd/GO catalyst (0.7 mol% Pd, 6 wt% F127) at 120 °C (Scheme 6). Under these conditions, the method delivers good to excellent yields ranging from 61% to 90%.

**Scheme 7:**
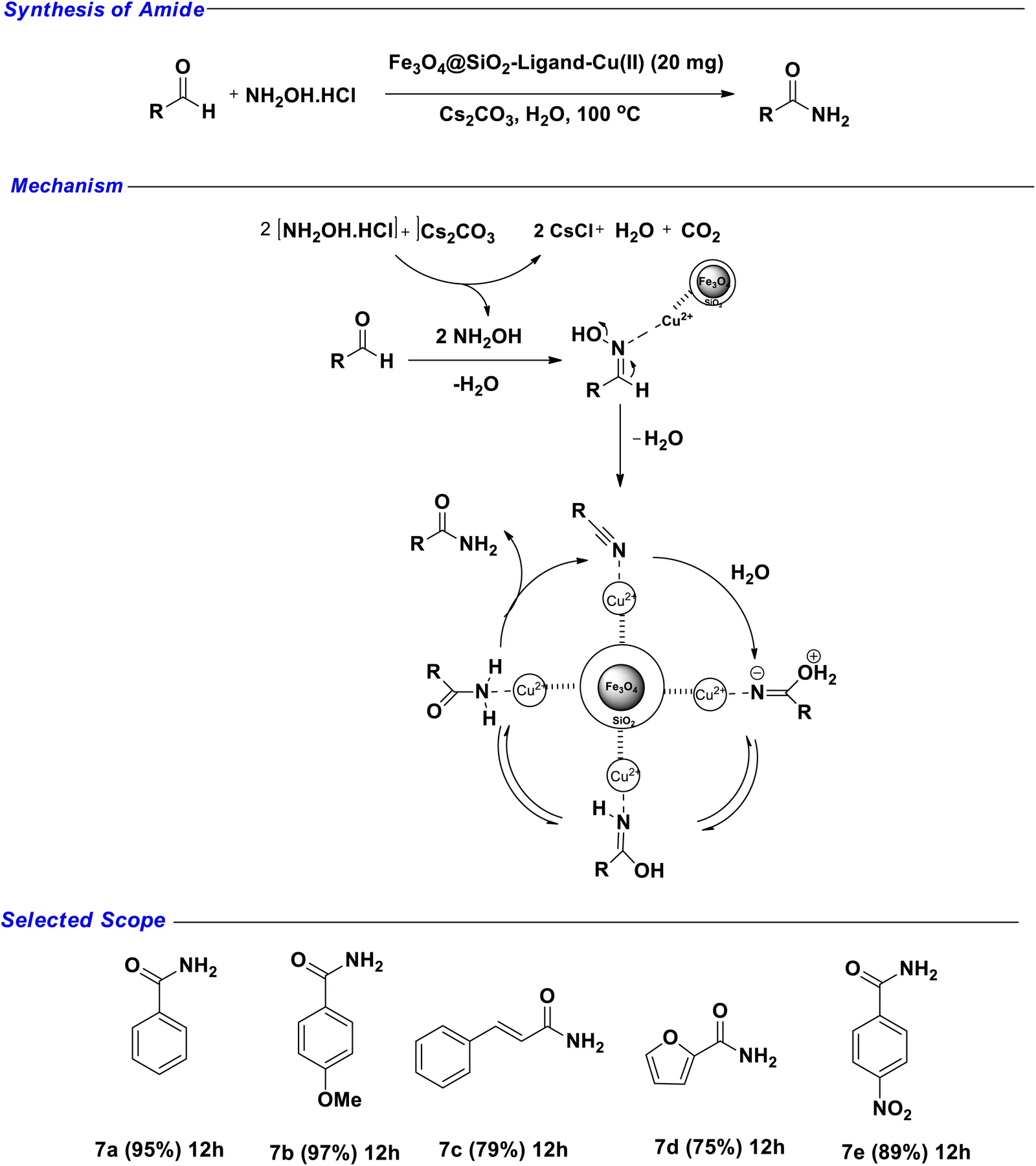
One-pot synthesis of primary amides using Cu(ii)@SMNP catalyst.

Amani and group reported an environmentally benign and efficient protocol for the one-pot synthesis of primary amides from aldehydes using a Cu(ii)-immobilized silica-coated magnetic nanocatalyst (Cu(ii)@SMNP) in aqueous medium.^51^ The catalytic system enables high-yield transformations under mild conditions, using hydroxylamine hydrochloride as the amine source, Na_2_CO_3_ as the base, and water as the sole solvent (Scheme 7). The influence of substituents on the aldehyde substrates was systematically investigated. Electron-donating groups (*e.g.*, –OH, –OCH_3_) facilitated faster conversion and higher yields, whereas electron-withdrawing groups (–NO_2_, –Cl) showed slightly reduced activity but still afforded good product formation. Various aromatic, aliphatic, and heterocyclic aldehydes were efficiently converted, demonstrating broad substrate scope and functional group tolerance. The Cu(ii)@SMNP catalyst was easily separated using an external magnet and reused for at least five consecutive cycles without significant loss of catalytic activity. They claimed this methodology provided a practical and green alternative for primary amide synthesis, highlighting the advantages of heterogeneous catalysis, water as a green solvent, and catalyst recyclability.

**Scheme 8:**
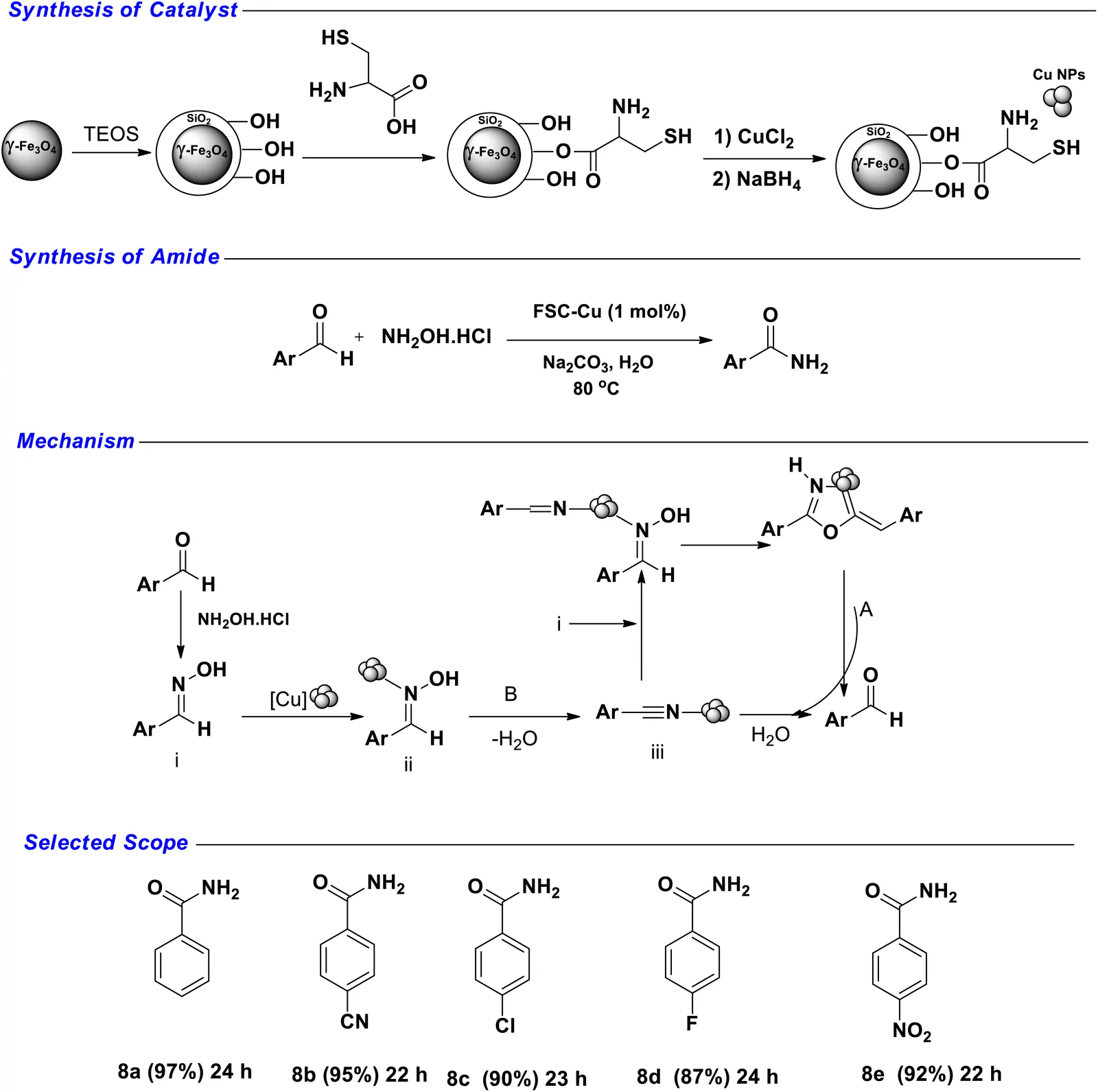
One-pot amidation of aldehydes by FSC-Cu catalyst.

Ziaee and colleagues have developed a magnetically recoverable, eco-friendly Cu-based heterogeneous catalyst for the one-pot transformation of aldehydes into their respective primary amides. The synthesis involved the covalent attachment of Fe_3_O_4_@SiO_2_ nanocomposites and bi-functional cysteine amino acid to the siliceous surface of the nanocatalyst.^52^ The amidation of aryl halides, whether containing electron-withdrawing or electron-donating groups, with hydroxylamine hydrochloride was effectively catalyzed by Fe_3_O_4_@SiO_2_@Cysteine-copper (FSC–Cu) magnetic nanoparticles (MNPs) in an aqueous environment, resulting in high product yields (87–97%) (Scheme 8). Furthermore, the FSC-Cu MNPs can be conveniently separated from the reaction mixture using an external magnet and reused for at least 8 cycles without a significant decrease in catalytic activity.

**Scheme 9:**
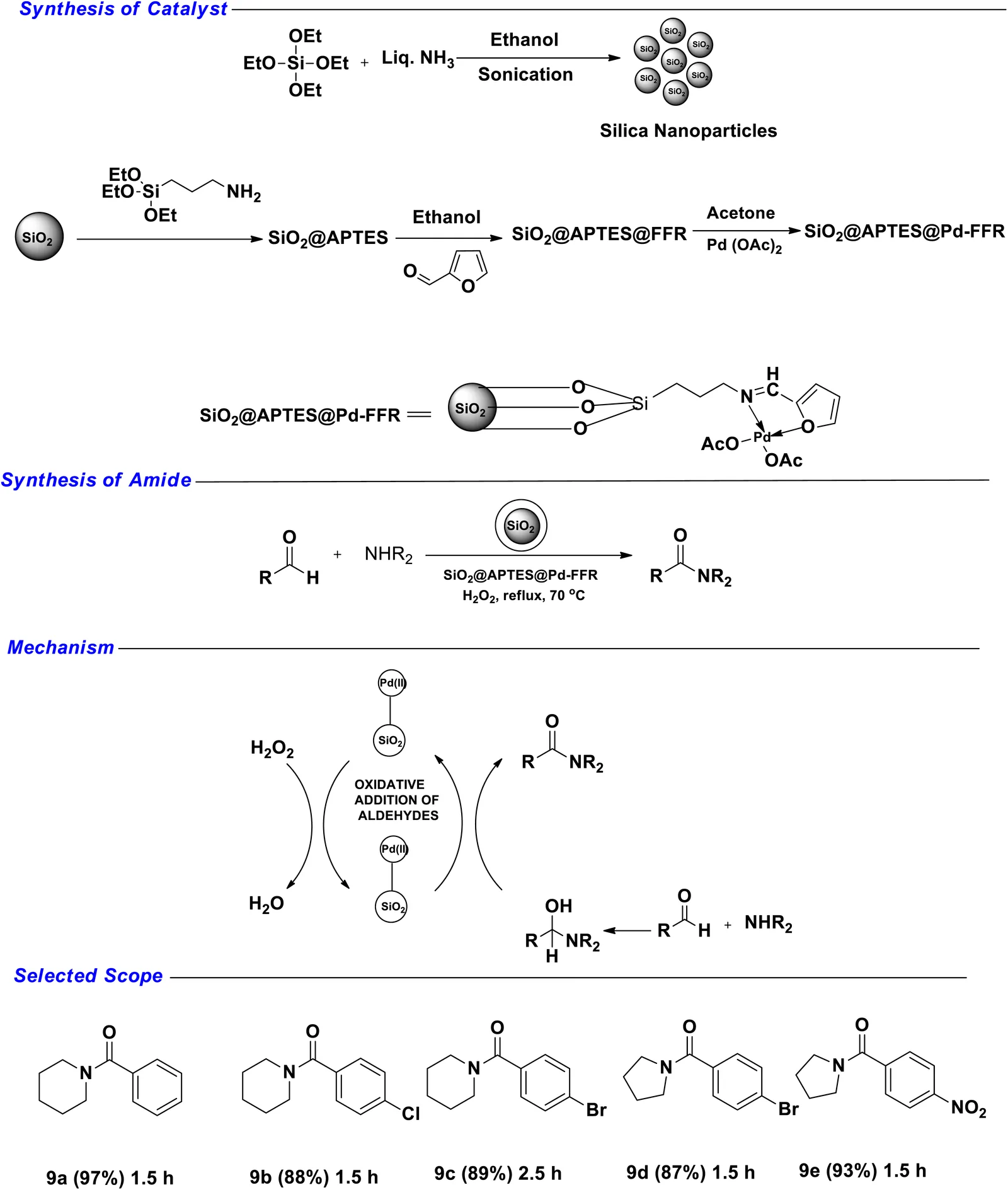
Silica nanosphere-supported palladium(ii) furfural complex catalyzed oxidative amination of aldehydes.

A silica nanosphere-supported palladium(ii) furfural complex (SiO_2_@APTES@Pd-FFR) was developed and used it as an efficient and recyclable catalyst for the oxidative amination of aldehydes under mild conditions, reported by Sharma and group.^53^ The reaction was performed using 20 mg of catalyst in the absence of solvent at 70 °C in the presence of H_2_O_2_ as the oxidant, affording the corresponding imines in excellent yields (81–99%) (Scheme 9). A wide range of aldehydes and primary amines, including those bearing electron-donating and electron-withdrawing substituents, such as aldehydes with electron-withdrawing groups, underwent amination faster in comparison than those with electron-donating groups. Mechanistic studies revealed that the transformation proceeds *via* a carbinolamine intermediate, followed by oxidation which provided amides. The catalyst exhibited excellent stability and reusability, maintaining high catalytic performance over at least five cycles. They claimed the system represents a sustainable, operationally simple approach to C–N bond formation using a heterogeneous palladium nanocatalyst and molecular oxygen as a green oxidant.

**Scheme 10:**
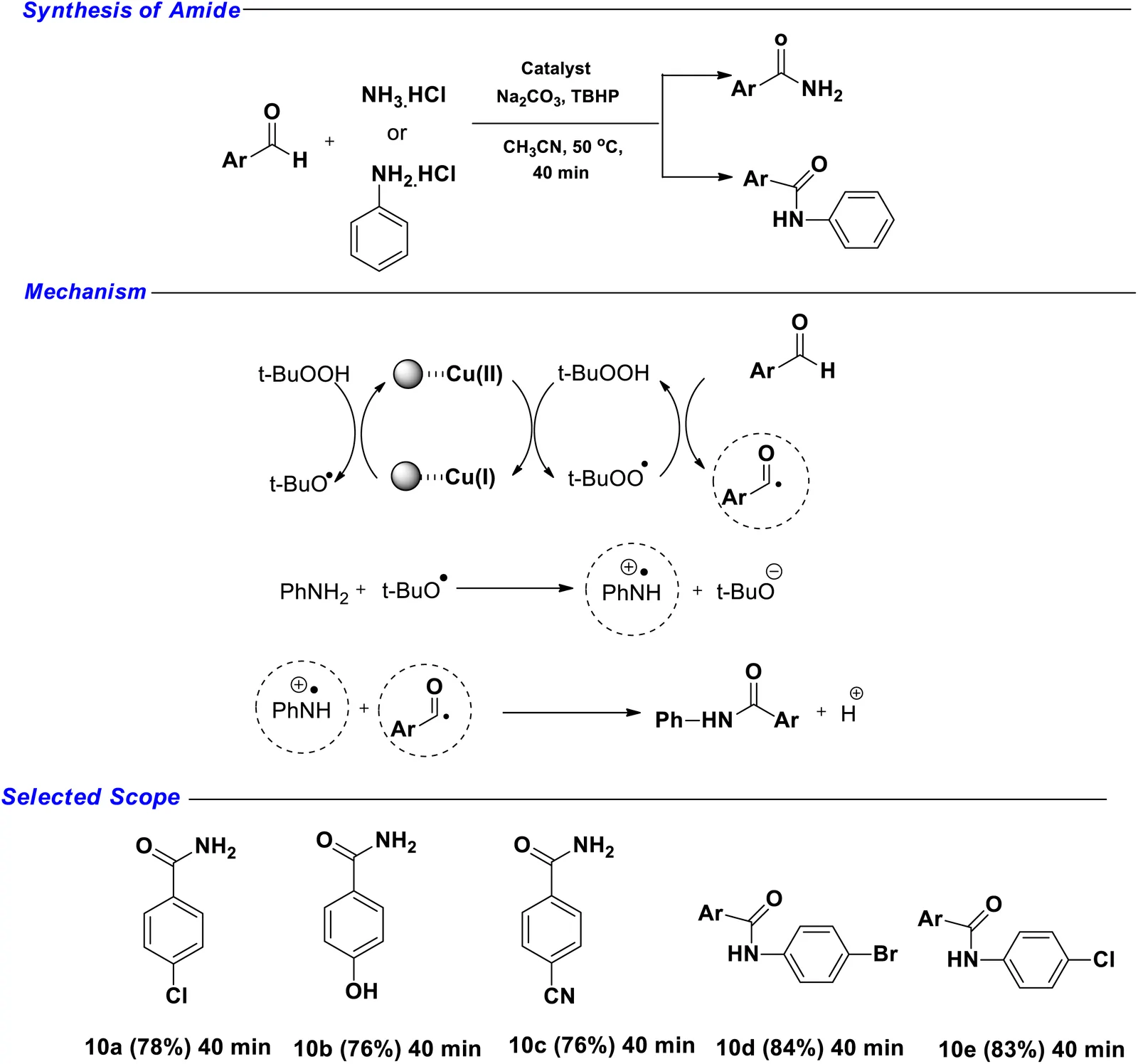
Oxidative amidation of aromatic aldehydes with ammonium hydrochloride.

Mamaghani and group synthesized a copper(ii) complex covalently anchored to a (2-iminomethyl)phenol moiety, supported on HAp-encapsulated a-Fe_2_O_3_, as an inorganic–organic hybrid magnetic nanocatalyst for the synthesis of primary and secondary amides.^54^ The reaction proceeds *via* oxidative amidation of aromatic aldehydes with ammonium hydrochloride and aniline hydrochloride in the presence of 10 mg of catalyst, Na_2_CO_3_ as base, and TBHP as the oxidant, under acetonitrile solvent conditions, affording good to excellent yields (74–84%) (Scheme 10) within 40–42 minutes. Aromatic aldehydes having both electron-donating and electron-withdrawing groups were converted to the related amides in good yields. In some cases, the secondary amides is slightly higher yield than the primary amides. This catalyst can be recovered and reused.

**Scheme 11:**
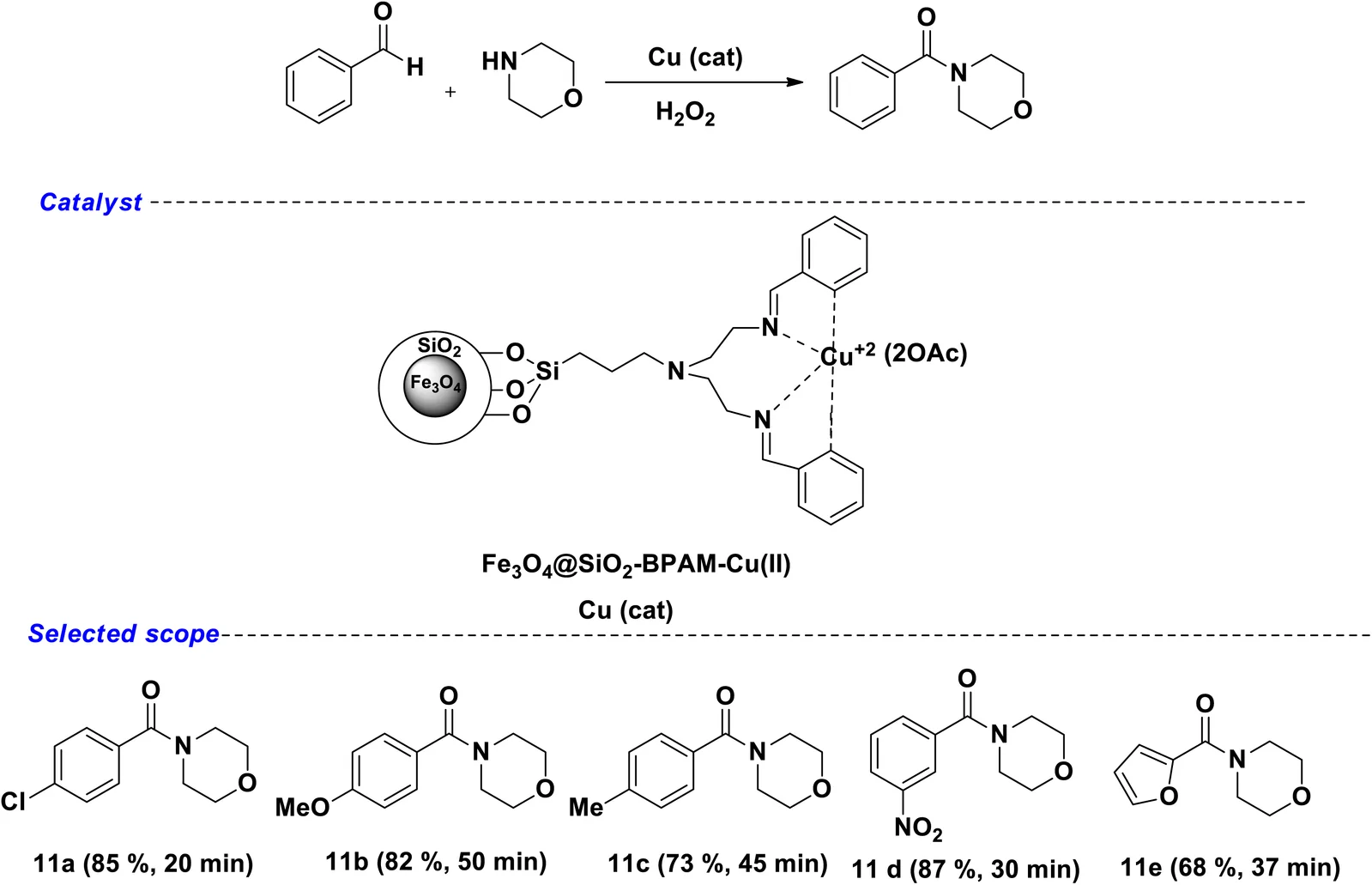
Oxidative amidation of aromatic aldehydes with morpholine.

Abdollahi-Alibeik and his group synthesized Fe_3_O_4_@SiO_2_ supported Schiff base-Cu(ii) complex (Fe_3_O_4_@SiO_2_BPAM-Cu(ii)) for oxidative amidation of aromatic aldehydes and alcohols with amines using hydrogen peroxide as an oxidizing agent for the synthesis of amides.^55^ They investigated using benzaldehyde or benzyl alcohol with morpholine and the mentioned catalyst, without an oxidant; the reaction did not proceed. The optimized reaction conditions are: aldehyde (1 mmol), amine (3 mmol), H_2_O_2_ (2 mmol), Fe_3_O_4_@SiO_2_-BPAM-Cu(ii) (0.05 g), at 50 °C under solvent-free conditions (Scheme 11). Under these conditions, the oxidative amidation of aromatic aldehydes with morpholine provided good to excellent product yields (68–92%).

### 4.3 Halide–isocyanide mediated amide formation

**Scheme 12:**
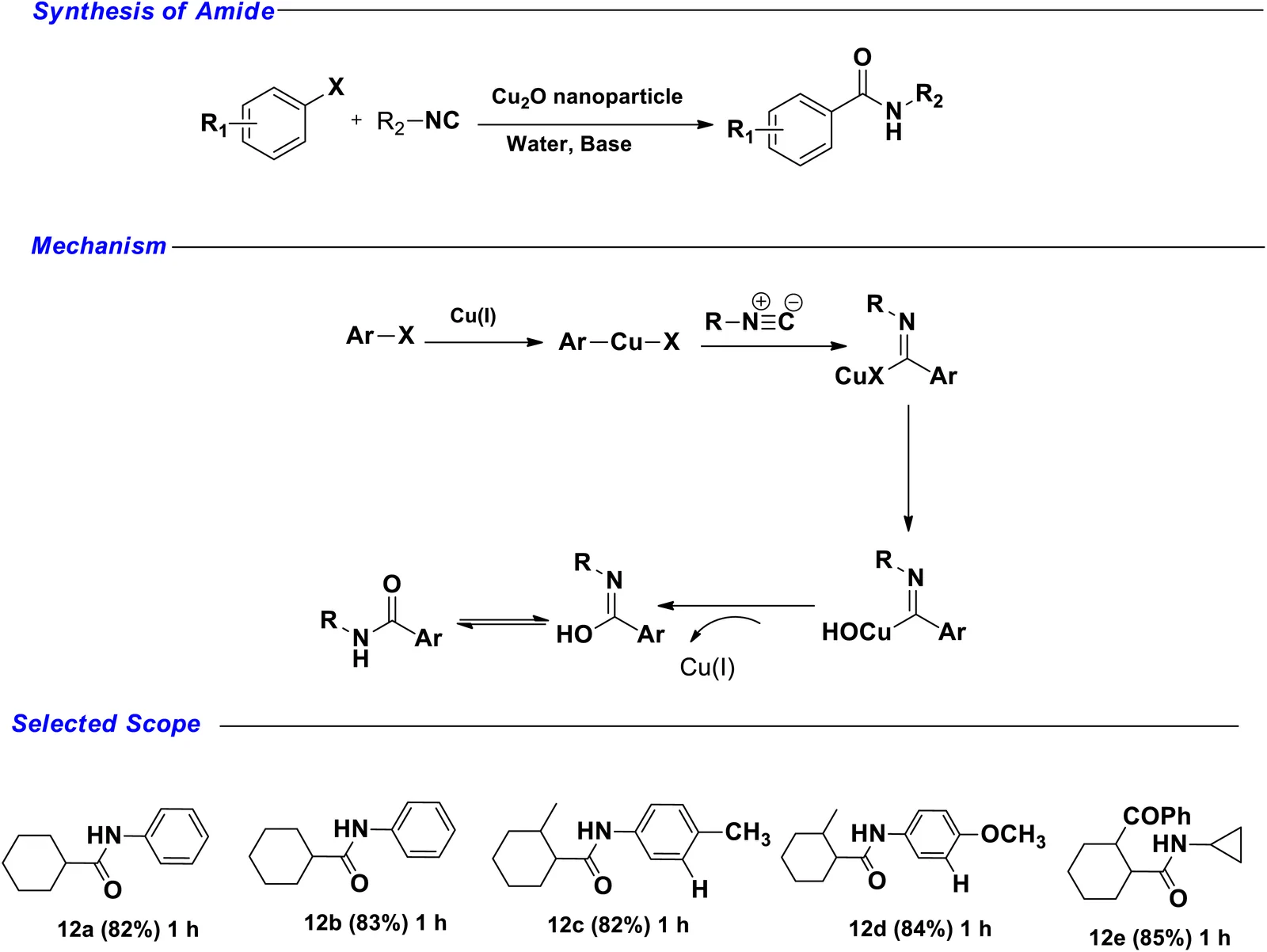
Amidation of aryl halides with isocyanides by using cubic copper(i) oxide nanoparticles.

Sen *et al.* reported that cubic copper(i) oxide nanoparticles are used to synthesize amides by amidation of aryl halides with isocyanides through C–C bond formation. This methodology proceeds smoothly with sonication at 80 °C in the presence of a base, 10 mol% of the optimized catalyst, under aerobic conditions, and in water as the medium^56^ (Scheme 12). The approach's scope and generality were investigated under optimized reaction conditions, utilizing aryl or aliphatic isocyanides paired with aryl halides. The amides produced achieved good to excellent yields (74–88%), regardless of whether aliphatic or aromatic isocyanides were used. Notably, bromide exhibited similar efficiency under the same conditions.

### 4.4 Direct carboxylic acid with formamidines

**Scheme 13:**
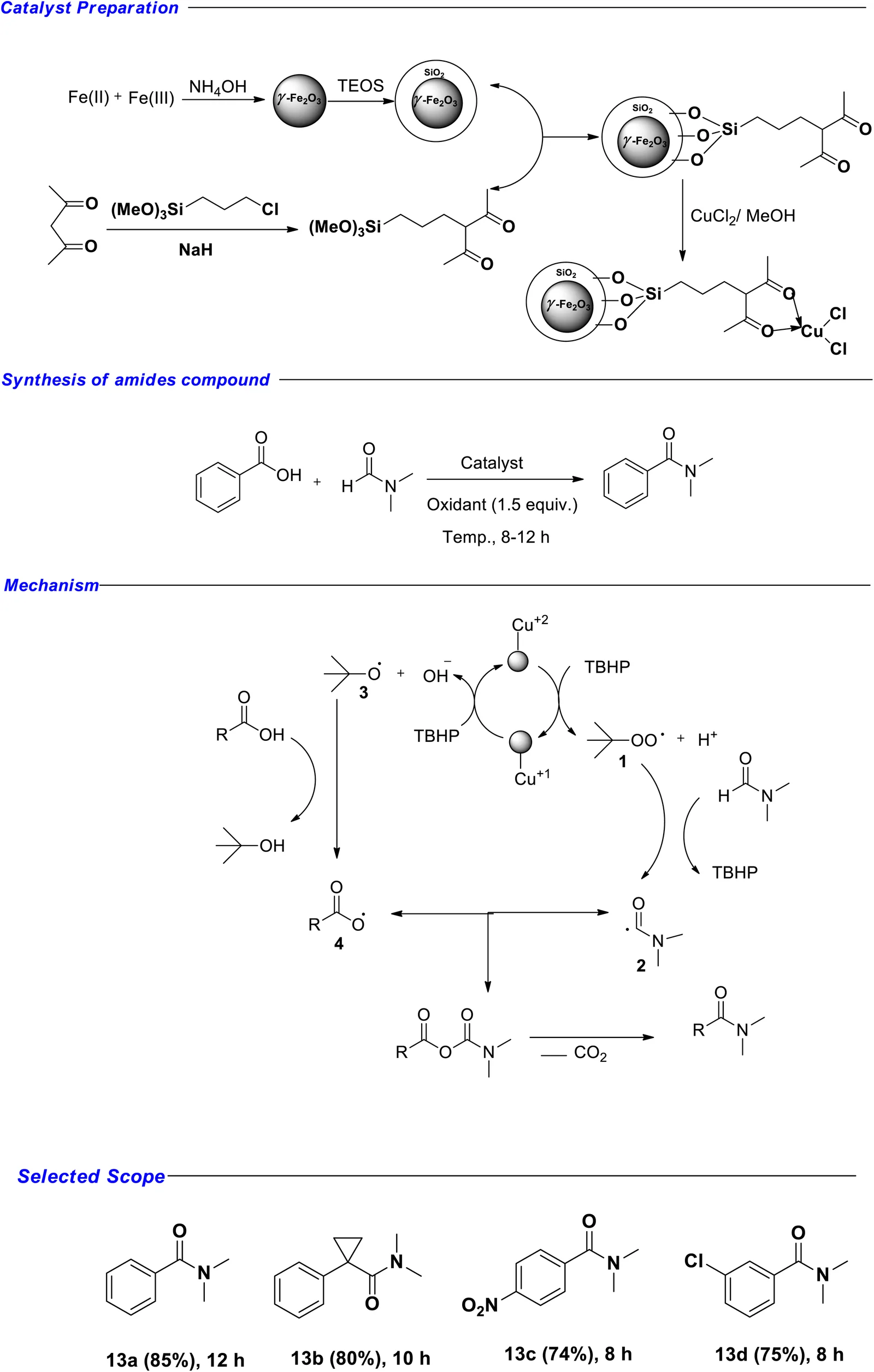
Direct synthesis of amides from carboxylic acid and formamidines by (γ-Fe_2_O_3_@SiO_2_eacacsileCu(ii)) catalyst.

In 2014, Heydari and the group reported that the modified magnetic nanoparticles coated silica and acetylacetone (γ-Fe_2_O_3_@SiO_2_eacacsil), followed by the addition of copper chloride.^57^ The catalyst (γ-Fe_2_O_3_@SiO_2_eacacsileCu(ii)) was explored for its catalytic activity in the oxidative coupling of carboxylic acids with *N*,*N*-dialkylformamides to synthesize amides and their derivatives in good to excellent yields (40–85%). Moreover, different derivatives of tertiary amides were also synthesized using the same methods in good yield. The optimized reaction conditions were 20 mg catalyst (0.46 mol%), sufficient for catalyzed the reaction at 80 °C (Scheme 13). The author also claimed that the magnetic properties of this nanocatalyst facilitated its easy separation and reusability.

**Scheme 14:**
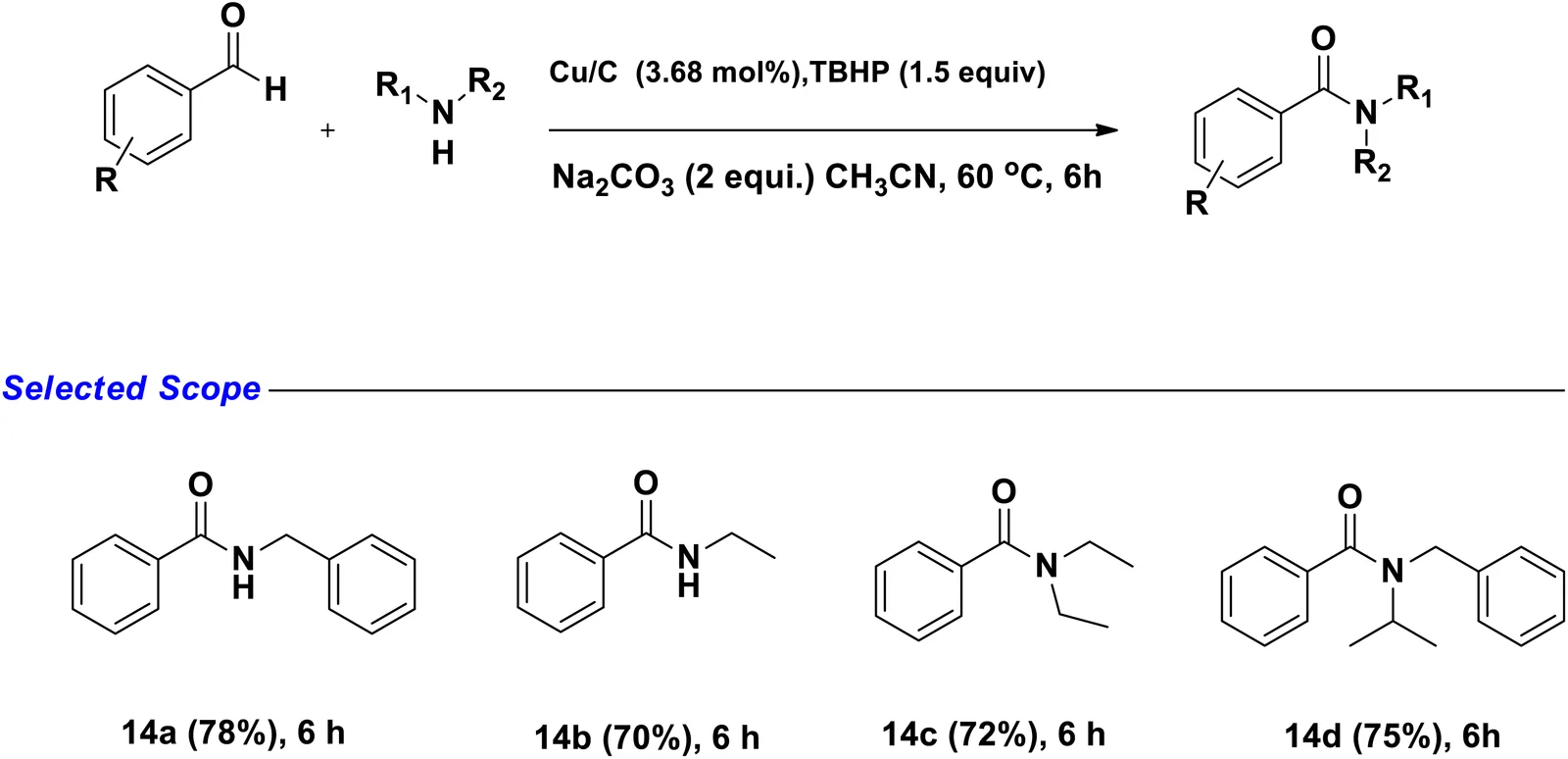
Oxidative coupling reaction for the synthesis of amides by modified charcoal with copper nanoparticles.

Niknam *et al.* reported modified charcoal by copper nano particles and used it as a heterogeneous catalyst for oxidative coupling reaction for synthesis of amides.^58^ In this methodology, aromatic aldehydes allowed to react with amine hydrochloride salts in presence Cu/C catalyst, TBHP as oxidant, and NaCO_3_ were used for the synthesis of amides. The optimized reaction conditions are 2 equiv. of Na_2_CO_3_ base, 1.5 equiv. of TBHP, 20 mg Cu/C catalyst (3.68 mol%), and 60 °C temperature, within 6 h, which provided 10–78% of amides (Scheme 14). Various benzaldehyde derivatives with *meta*- and *para*-substituents reacted with ammonium salts to yield primary amides in moderate to good yields, while *ortho*-substituted derivatives gave no products due to steric hindrance. Secondary and tertiary amides are also synthesized with this protocol using primary and secondary amine hydrochloride salts with benzaldehyde. Electron delocalization and reduced yields for some amines; using free amines instead of hydrochloride salts resulted in lower yields. Moreover, the catalyst can be reused and recovered.

### 4.5 Catalytic direct amination of alcohols

**Scheme 15:**
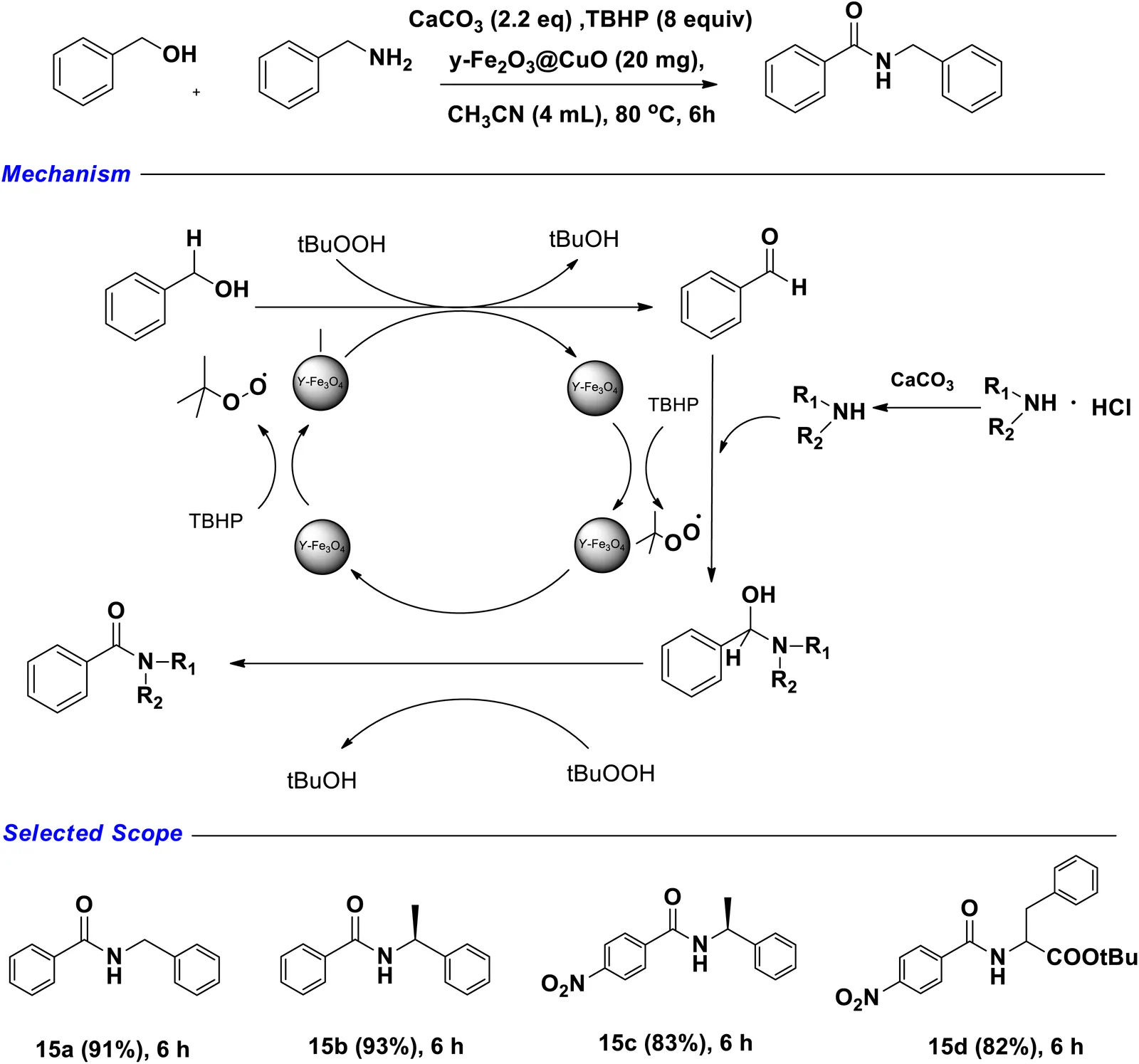
Direct amination of alcohols by maghemite-copper oxide nanocomposite catalyst.

Hassani *et al.* reported and synthesized Maghemite-copper oxide nanocomposite catalyst to synthesize amides. The reaction of alcohols and amines with 20 mg of catalyst, TBHP as the oxidant, and CaCO_3_ as the base in acetonitrile at 80 °C yields good to excellent yields of products^59^ (Scheme 15). This study indicated that aliphatic amines, including allyl amine, butyl amine, and 2-amino-1-butanol, yielded corresponding amides with efficiencies of 51%, 68%, and 72%, respectively. The reaction was particularly effective with benzyl amine and (S)-α-methylbenzyl amine, yielding excellent amide. However, the imidazole reaction proved problematic, with no amide formation observed. Furthermore, benzyl alcohols containing electron-withdrawing groups produced higher yields than those with electron-donating groups. In cases where benzyl alcohols had electron-donating substituents, the oxidation to acid occurred more rapidly than the amidation process.

**Scheme 16:**
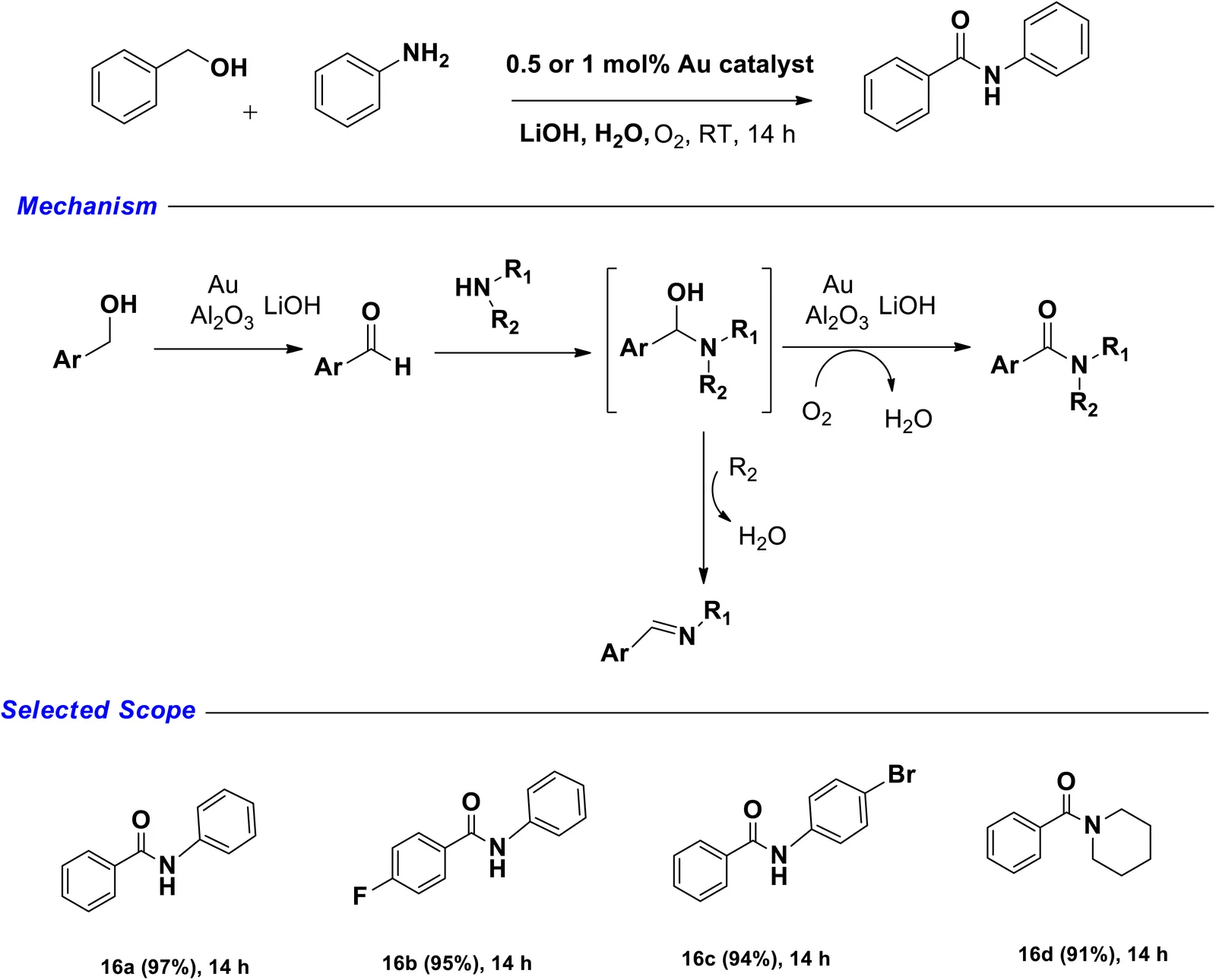
Direct synthesis of amides from alcohols with amines by Au/mesoporous Al_2_O_3_ nanocatalyst.

Chng *et al.* reported Au/mesoporous Al_2_O_3_ nanocatalyst to synthesize amides using alcohol and amines.^60^ The reaction was performed at room temperature and under atmospheric O_2_ pressure to form amide products with up to 97% isolated yield. The optimized reaction conditions are 0.5 mol% Au catalyst, LiOH base, in the presence of O_2_ at RT, within 14 h, producing good to excellent product yields (30–97%) (Scheme 16). In the case of substituted benzyl alcohols, electron-withdrawing groups give good yields, while donating groups give lower yields. On the other hand, in substituted amines aromatic amines with the weakly electron-donating at the *ortho*, *meta*, or *para* position gave good yields of the desired amides (85–89%). Electron-withdrawing halogen groups at either the *meta* or *para* position of the anilino moiety also led to good to excellent products.

### 4.6 Acylation of amines for amide bond formation

**Scheme 17:**
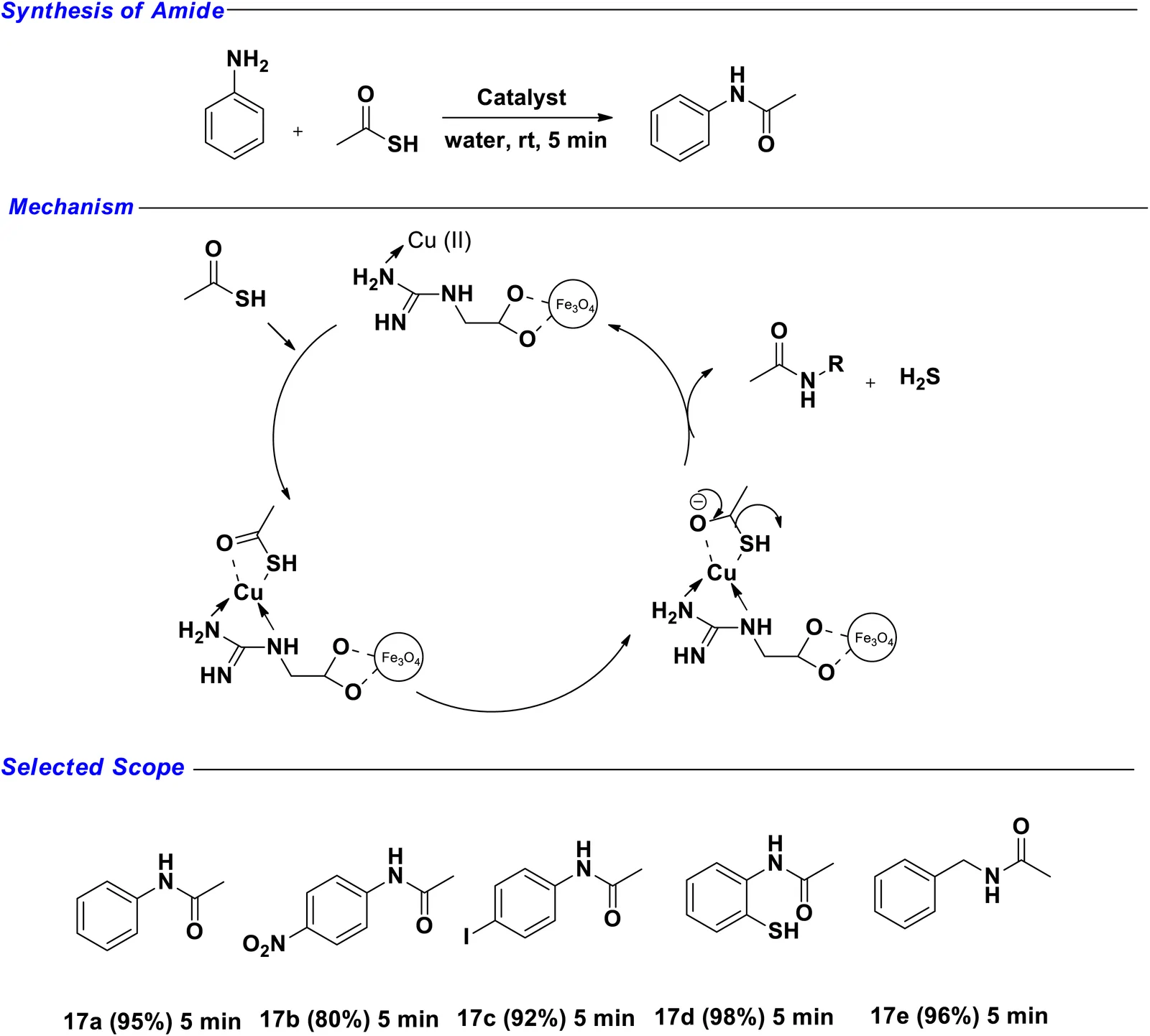
Synthesis of amides by *N*-acylation of various amines catalyzed by Fe_3_O_4_@GAA-Cu(ii).

Miraki *et al.* developed copper-grafted guanidine acetic acid-modified magnetite nanoparticles (Fe_3_O_4_@GAA-Cu(ii)) and used it as a green, superparamagnetic and recoverable nanocatalyst for the synthesis of amides by *N*-acylation of various amines in a very short time with an equimolar amount of thioacetic acid in water at room temperature^61^ (Scheme 17). This method is found to be highly selective for amines and insensitive to other functional groups. It is observed that only the primary amino group will undergo *N*-acylation under these conditions. Tryptophan is successfully *N*-acetylated with excellent yield (89%). Electron-withdrawing groups such as *p*-nitroaniline and *p*-iodoaniline undergo *N*-acetylation, except for *p*-nitroaniline (80%); other *N*-acetylated products are isolated in excellent yields (88–98%). The authors claimed that Mild reaction conditions, high selectivity and efficiency, a simple workup, and excellent yields are among the major advantages of the procedure.

**Scheme 18:**
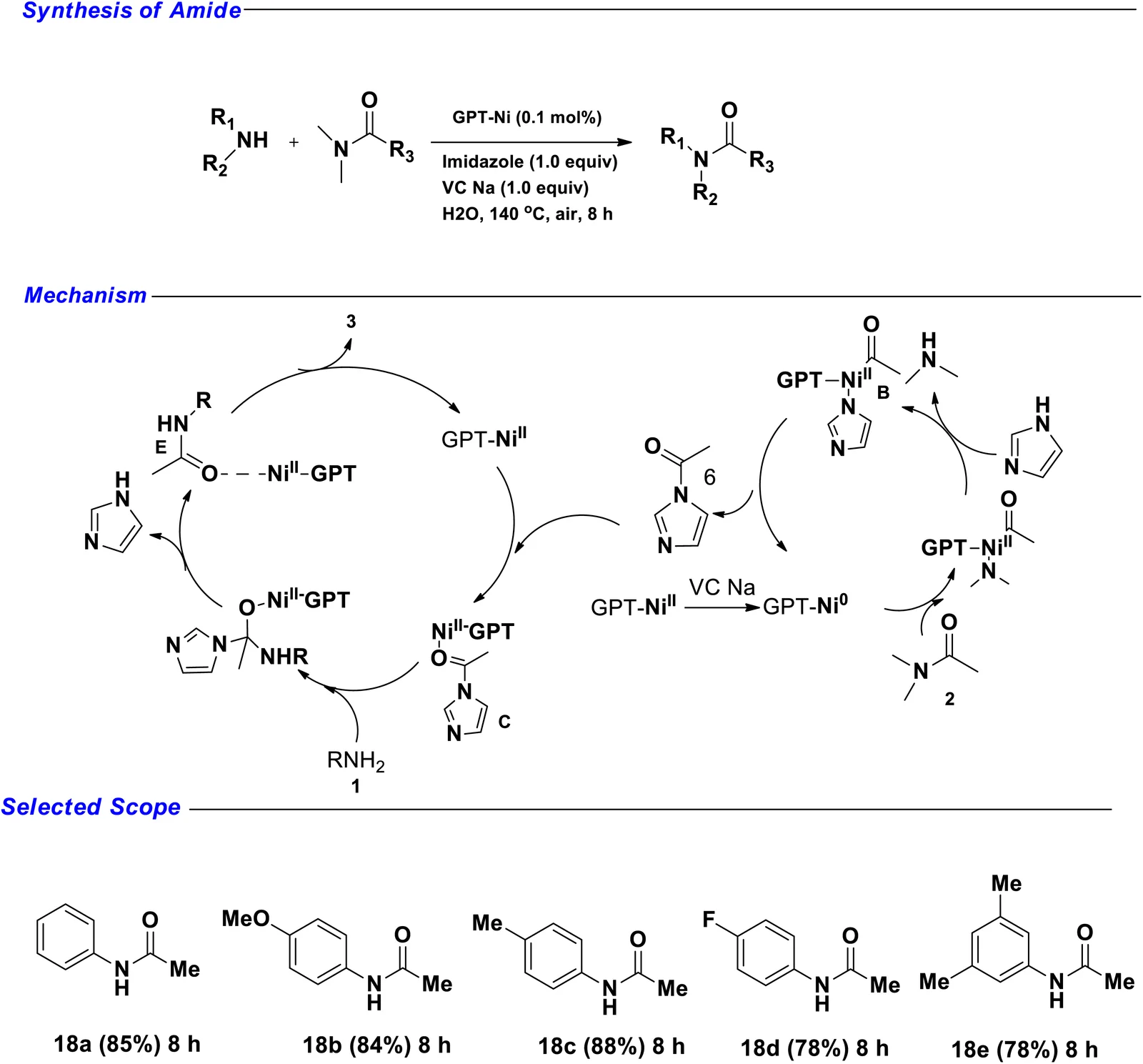
GPT-Ni catalyzed *N*-acylation of amines using DMA or *N*,*N*-dimethylpropionamide (DMP).

Lin and group synthesized Glycosylpyridyl-Triazole@Nickel Nanoparticles (GPT-Ni) and used it as a heterogeneous nanocatalyst for acylation of amines with *N*,*N*-dimethylacetamide (DMA), *N*,*N*-dimethylpropionamide (DMP), and *N*,*N*-dimethylformamide (DMF) as acylating reagents in water.^62^ The reaction carried out in the presence of 0.1 mol% of catalyst at 140 °C temperature for 8 h affords good to excellent yields (40–88%) (Scheme 18). The catalyst can be recovered and reuse *i.e.* the activity of the catalyst remains stable after six times of recycles.

**Scheme 19:**
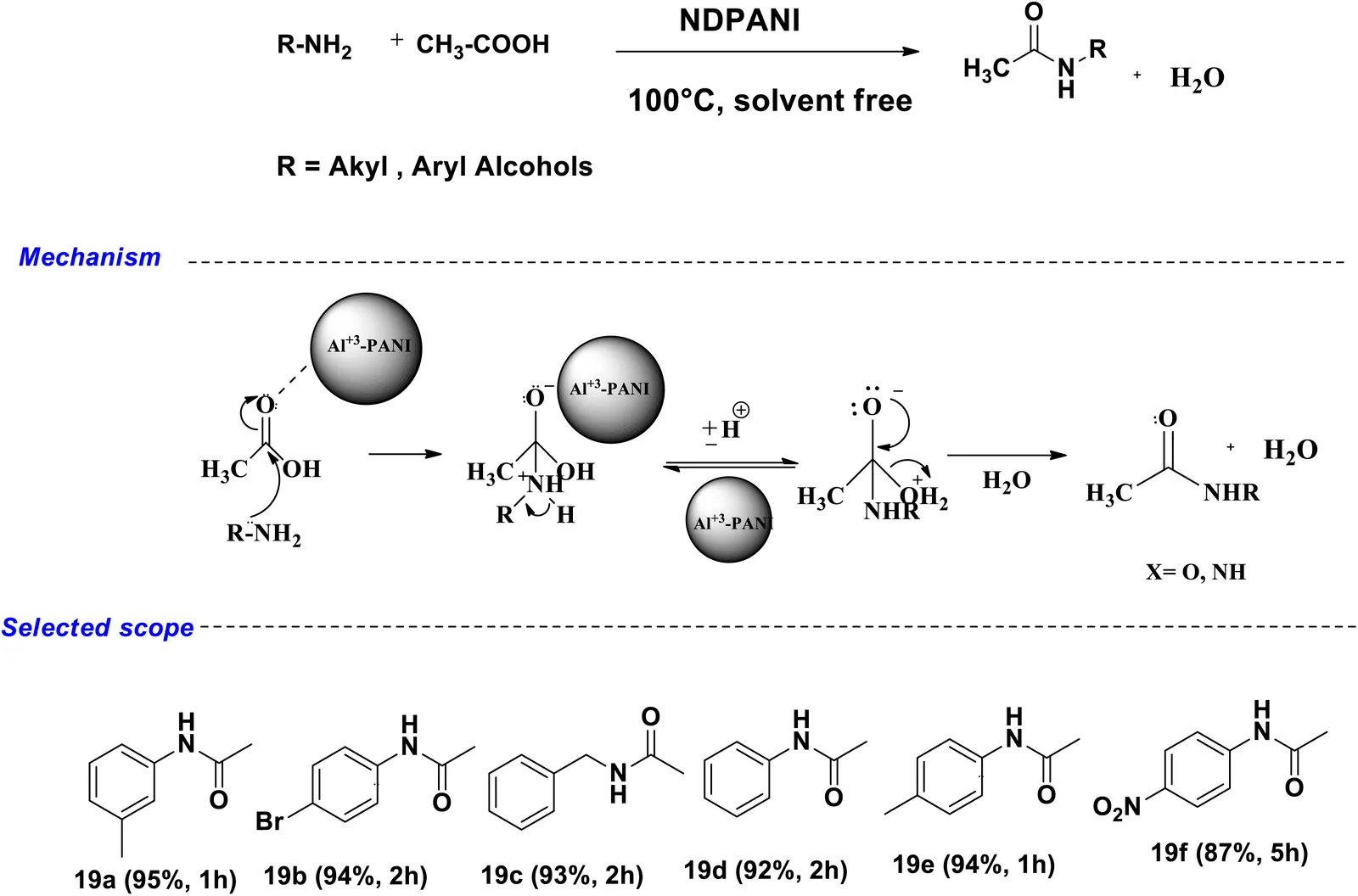
NDPANI catalyzed acylation of amines with acetic acid under solvent free reactions.

In 2021, our group (Behera *et al.*) reported the modification of nanopolyaniline with potash alum and explored its catalytic activity in the acylation of alcohols and amines with acetic acid as an acylating agent.^63^ In this methodology, acylation of amine reactions was carried out under solvent-free conditions at 100 °C using 0.3 mol% of catalyst (NDPANI), yielding good to excellent product yields (86–95%) (Scheme 19). The protocol revealed that phenol reacted more slowly compared to amines. Among the amines, secondary amines were less reactive and took more time than primary aromatic amines and aniline. Also, aromatic amines having electron-withdrawing groups, such as *p*-nitroaniline, showed slower reactivity than those with electron-donating groups like *p*-bromoaniline. The advantages of this catalyst are that it is easy to prepare, inexpensive, non-hazardous, reusable (up to 5 cycles) and easy to handle.

### 4.7 Catalytic coupling of ketones and amines for amide formation

**Scheme 20:**
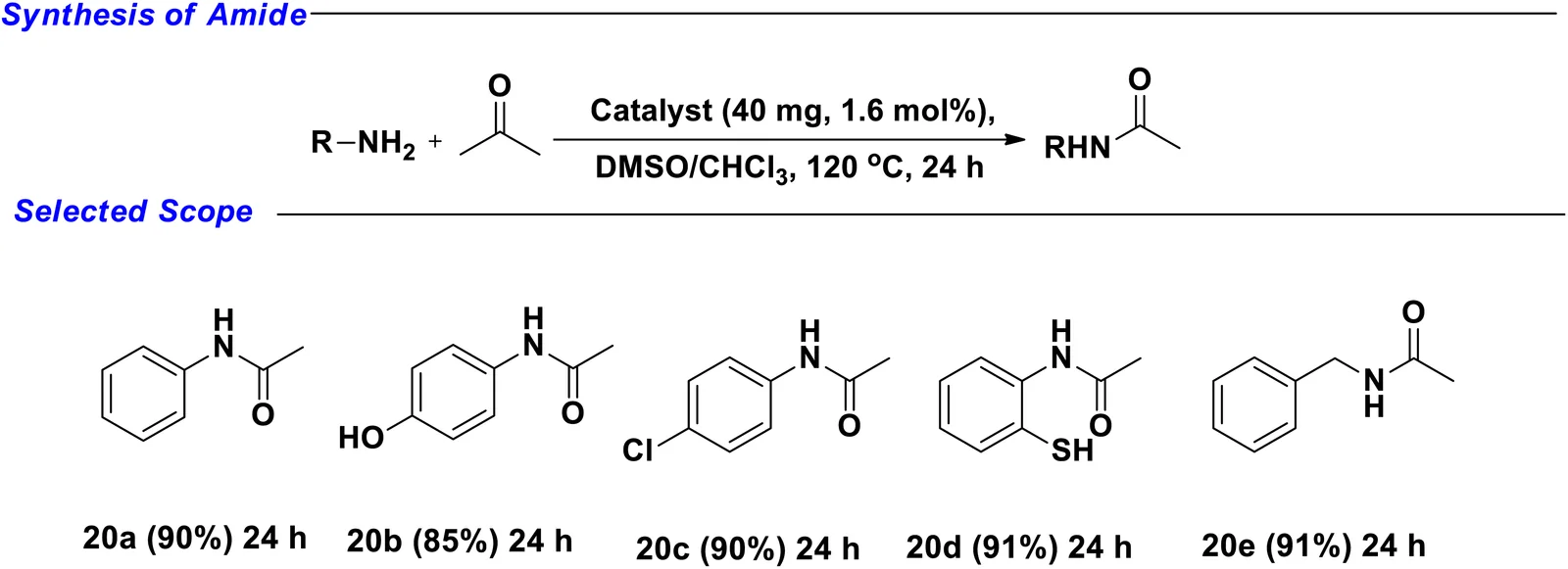
MN-tric-Cu catalyzed oxidative cleavage of ketones with amines.

Yazdani *et al.* developed copper supported on magnetite nanoparticles, modified with the sustainable ligand tricine (MN-tric-Cu), for the oxidative cleavage of ketones with amines to synthesize acetamides, specifically targeting C–C bond interactions.^64^ This methodology optimized the reactions using 40 mg of catalyst in DMSO/CHCl_3_ (1 : 1) at 120 °C in 24 h provided excellent yields of amides (75–91%) (Scheme 20). There is no noticeable difference was observed between electron donating and electron withdrawing substituents. Moreover, this catalyst can be recovered and reused several times.

### 4.8 Hydrogenation of α-hydroxy amides

**Scheme 21:**
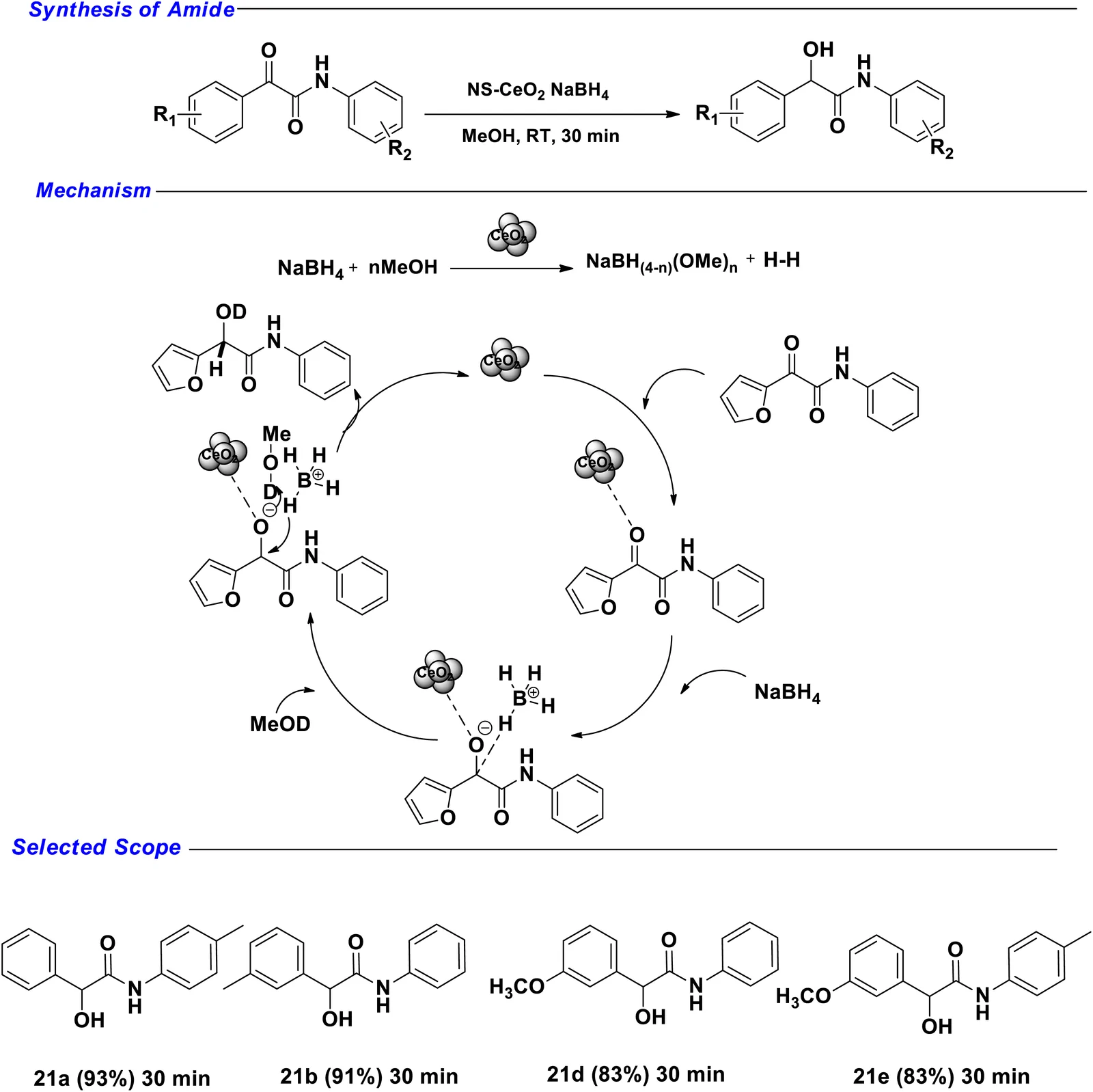
Chemoselective hydrogenation of deactivated α-keto amides by nanoceria catalyst.

Mishra *et al.* described a versatile, efficient, and recyclable ceria nanosphere (NS) catalyst synthesized through hydrothermal methods using oxalic acid as a capping agent.^65^ This nanoceria catalyst facilitated the chemoselective hydrogenation of deactivated α-keto amides in the presence of NaBH_4_, with the reaction failing to proceed in the absence of the catalyst. Various substituted hydroxy amides were synthesized with high yields (71–96%) and minimal catalyst usage (2 mol%) at 60 °C in 30–60 min (Scheme 21). The yield of the resulting products was influenced by the presence of both electron-withdrawing and electron-donating groups on the keto benzene ring. Notably, sensitive functional groups, including fluoro, chloro, bromo, and iodo groups, remained unaffected by the reducing agent. Additionally, electron-donating groups such as methyl (Me) and methoxy (OMe) gave good yields of the corresponding products, whereas weakly electron-withdrawing groups yielded excellent yields, likely due to reduced electron density on the carbonyl carbon. Interestingly, the presence of a *p*-chloro group in the substrate yielded the product.

### 4.9 Hydration of nitriles

**Scheme 22:**
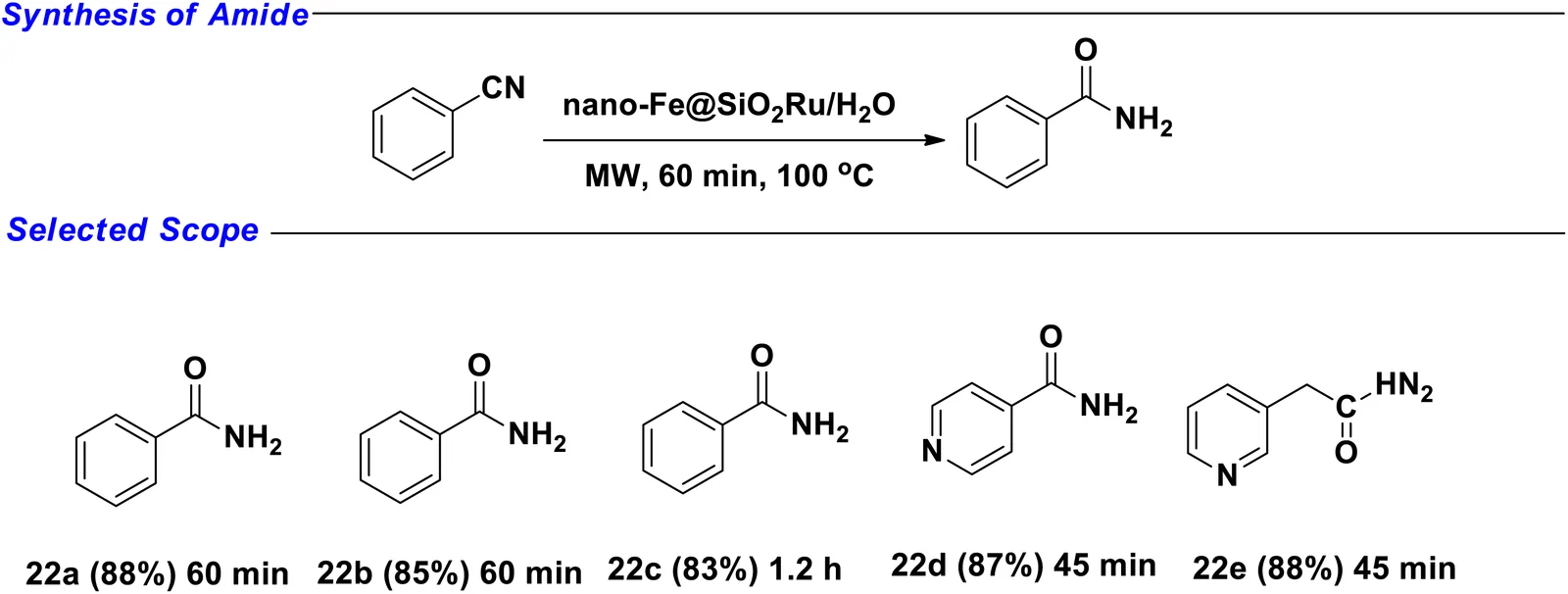
One-pot synthesis of amides from nitriles *via* hydration catalyzed by nano-Fe@SiO_2_Ru.

Varma *et al.* used ruthenium hydroxide nanoparticles on magnetic silica (nano-Fe@SiO_2_Ru) for one-pot synthesis of amides from nitriles by hydration.^66^ In this methodology, 100 mg of catalyst was successfully catalyzed under MW at 100 °C in water for 40 min to 2.5 h to obtain a good to excellent yield of product (72–88%) (Scheme 22). The hydration of various nitriles was investigated, revealing that the catalyst exhibited significant activity for the hydration of both activated and inactivated nitriles, including aliphatic and heterocyclic types, in pure water. They demonstrated the efficient conversion of several benzonitrile derivatives and aliphatic nitriles to their corresponding amides. The reaction rates were minimally affected by the electronic properties of the substituents on the benzonitrile aromatic ring. Notably, the hydration of isonicotinonitrile *N*-oxide did not produce the expected isonicotinoamide *N*-oxide; instead, isonicotinoamide was formed. Additionally, it was noteworthy that the hydration of 1,4-dicyanobenzene depended on time. A 45-minute exposure to microwave irradiation at 100 °C resulted in the selective formation of the monohydrated derivative, whereas a 2.5-hour exposure led to complete dihydration of 1,4-dicyanobenzene, yielding terephthalamide in quantitative yield.

**Scheme 23:**
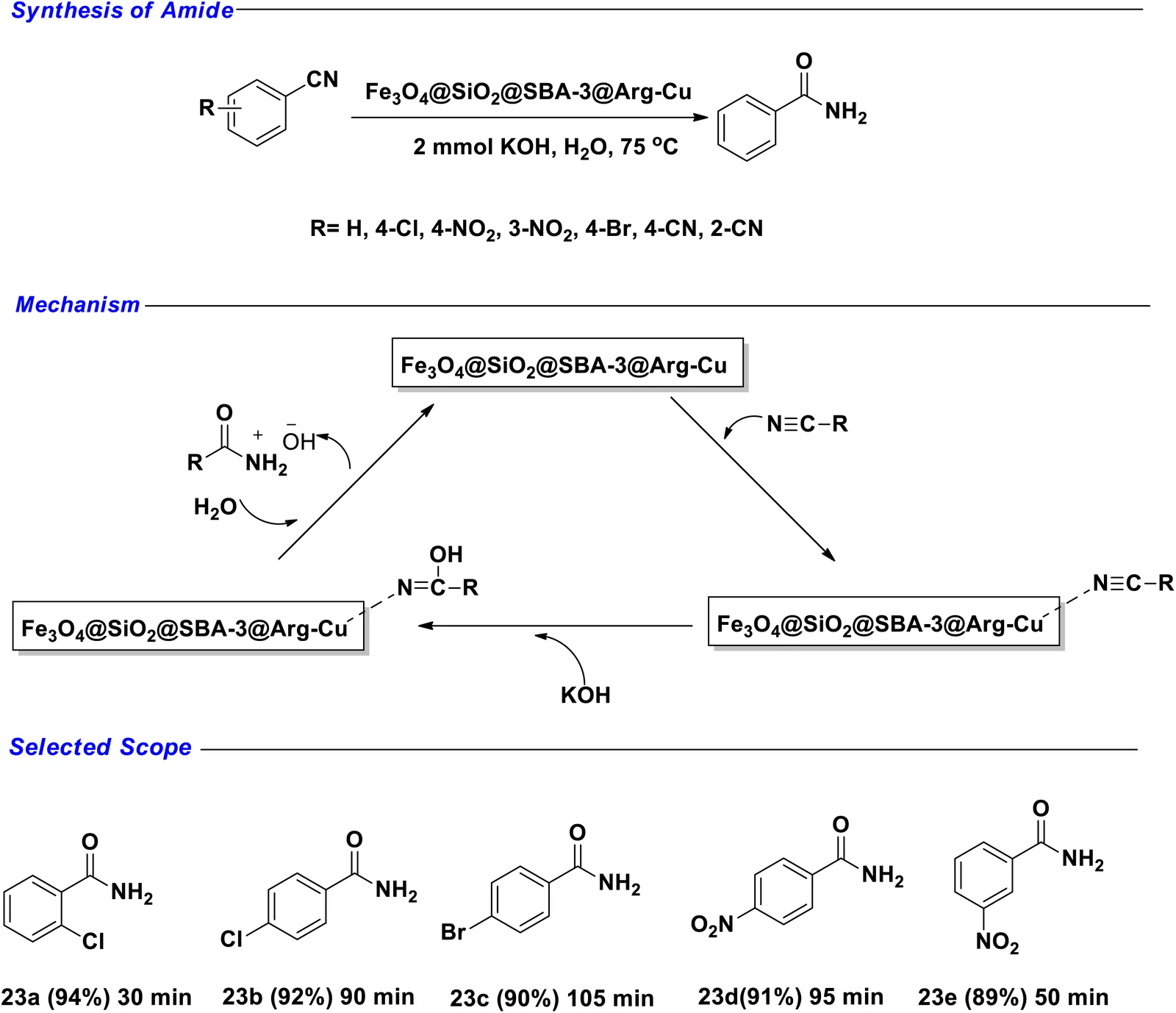
Fe_3_O_4_@SiO_2_@SBA-3@CPTMS@Arg-Cu nanocatalyst catalyzed synthesis of amides from nitriles.

Heidarnezhad and group synthesized the Fe_3_O_4_@SiO_2_@SBA-3@CPTMS@Arg-Cu nanocatalyst by grafting l-arginine onto a mixed-phase magnetic mesoporous SBA-3 support.^67^ This catalyst was used for the synthesis of amides from nitriles, with 40 mg of catalyst in water at 75 °C, and it provided an excellent yield of product (83–94%) (Scheme 23). The methodology offered green reaction conditions, shorter reaction time and reusability of the catalyst up to four cycles.

**Scheme 24:**
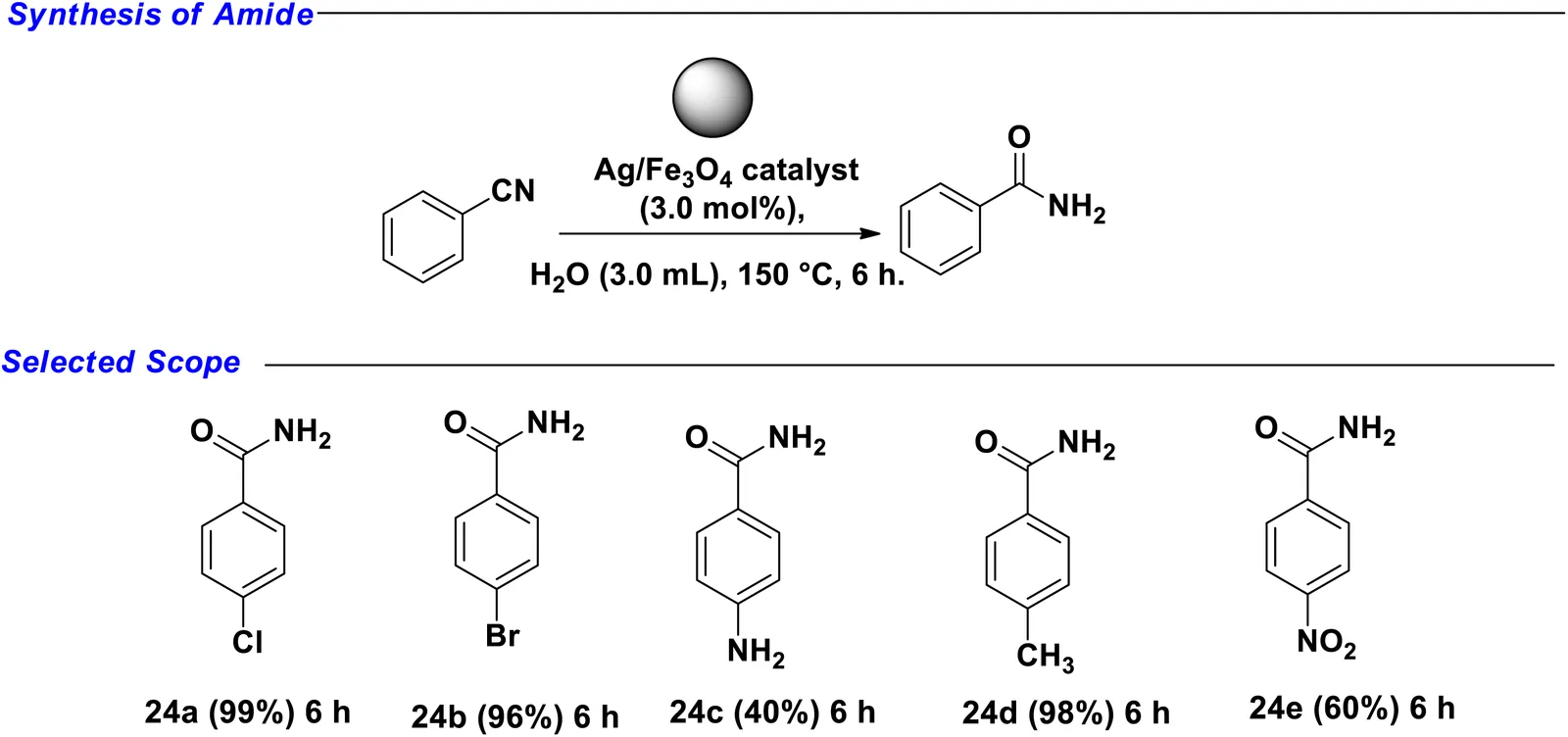
Hydration of nitriles catalyzed by Ag/Fe_3_O_4_ catalyst.

A Fe_3_O_4_-supported Ag nanocatalyst was synthesized and used for the selective hydration of nitriles to amides in water, as reported by Park and group.^68^ In the hydration of nitriles, the reaction proceeds with 3.0 mol% Ag/Fe_3_O_4_ catalyst and 3 mL of H_2_O, producing lower to excellent yield (13–99%) (Scheme 24). The reaction rates were not influenced substantially by the electronic effects of the substituents on the aromatic rings of nitriles. Both 4-chlorobenzonitrile and 4-bromobenzonitrile showed high conversions of more than 95%. The nitro and amino groups on the nitrile were less active than the halide substituents. When the hydration of *o*-, *m*-, and *p*-tolunitriles takes place, the steric effects of *ortho*-substituted nitriles decrease the rate of reaction and yield were observed. The hydration of aliphatic nitriles, such as acetonitrile and acrylonitrile, was also achieved with high conversions. The catalyst can be easily recovered with a magnet and reused.

### 4.10 Aerobic oxidative decarboxylative ammoxidation

**Scheme 25:**
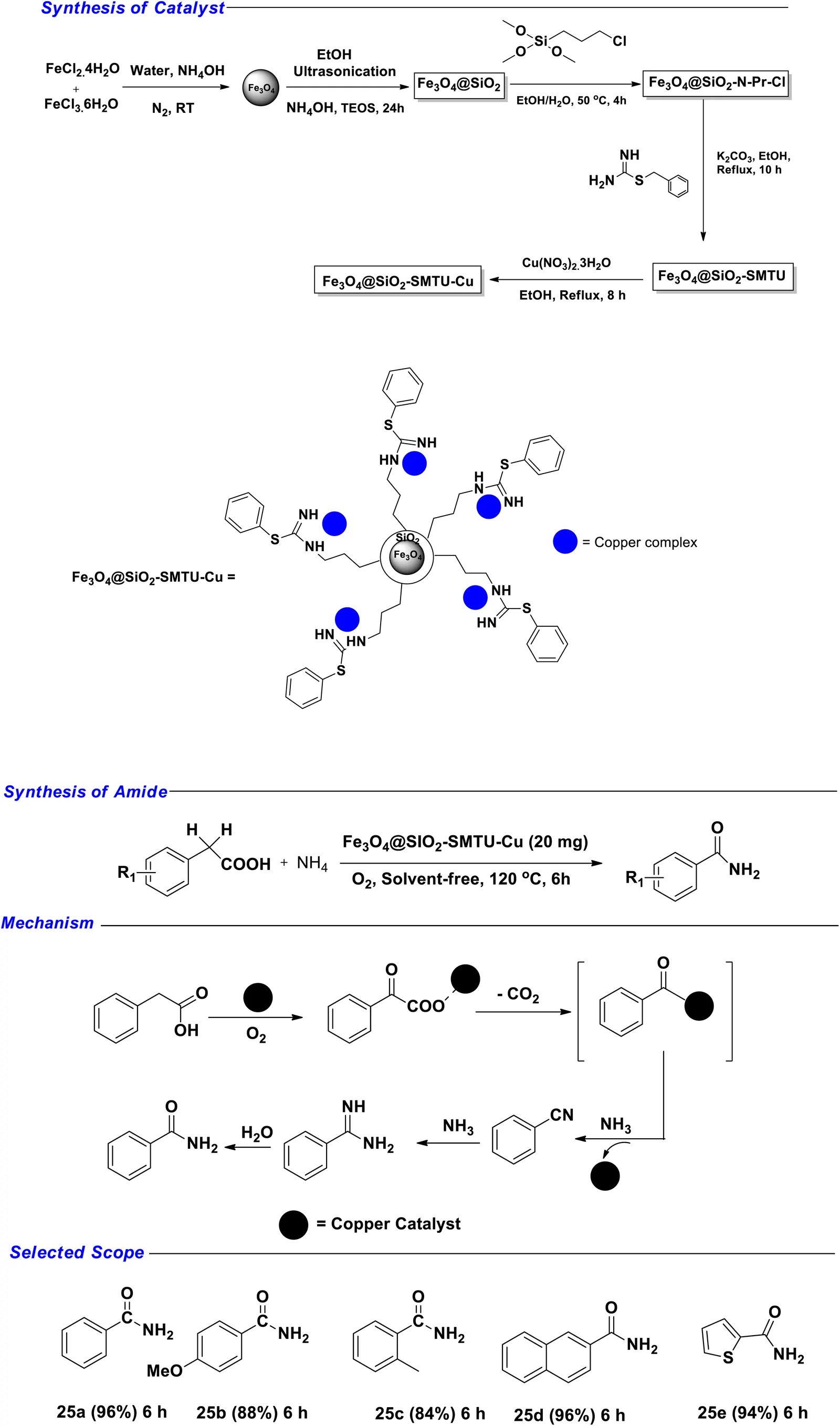
Aerobic oxidative decarboxylative ammoxidation catalyzed by Fe_3_O_4_@SiO_2_-SMTU-Cu.

Riadi *et al.* developed a copper(ii) complex supported on the surface of magnetic nanoparticles modified with S-benzylisothiourea (Fe_3_O_4_@SiO_2_-SMTU-Cu) catalyst.^69^ The catalytic activity of this catalyst was explored for the synthesis of amides *via* aerobic oxidative decarboxylative ammoxidation. The reaction of substituted phenylacetic acid with ammonia was explored under solvent-free conditions at 120 °C, with 20 mg of catalyst yielding an excellent yield of the product (76–96%) (Scheme 25). The author claimed this method gives notable advantages, such as easy separation of the catalyst by external magnetic field, excellent yields, short reaction times, nontoxic metal catalyst, and simplicity of operation, making this method a facile tool for the synthesis of amides.

### 4.11 Reductive-acetylation of nitroarenes

**Scheme 26:**
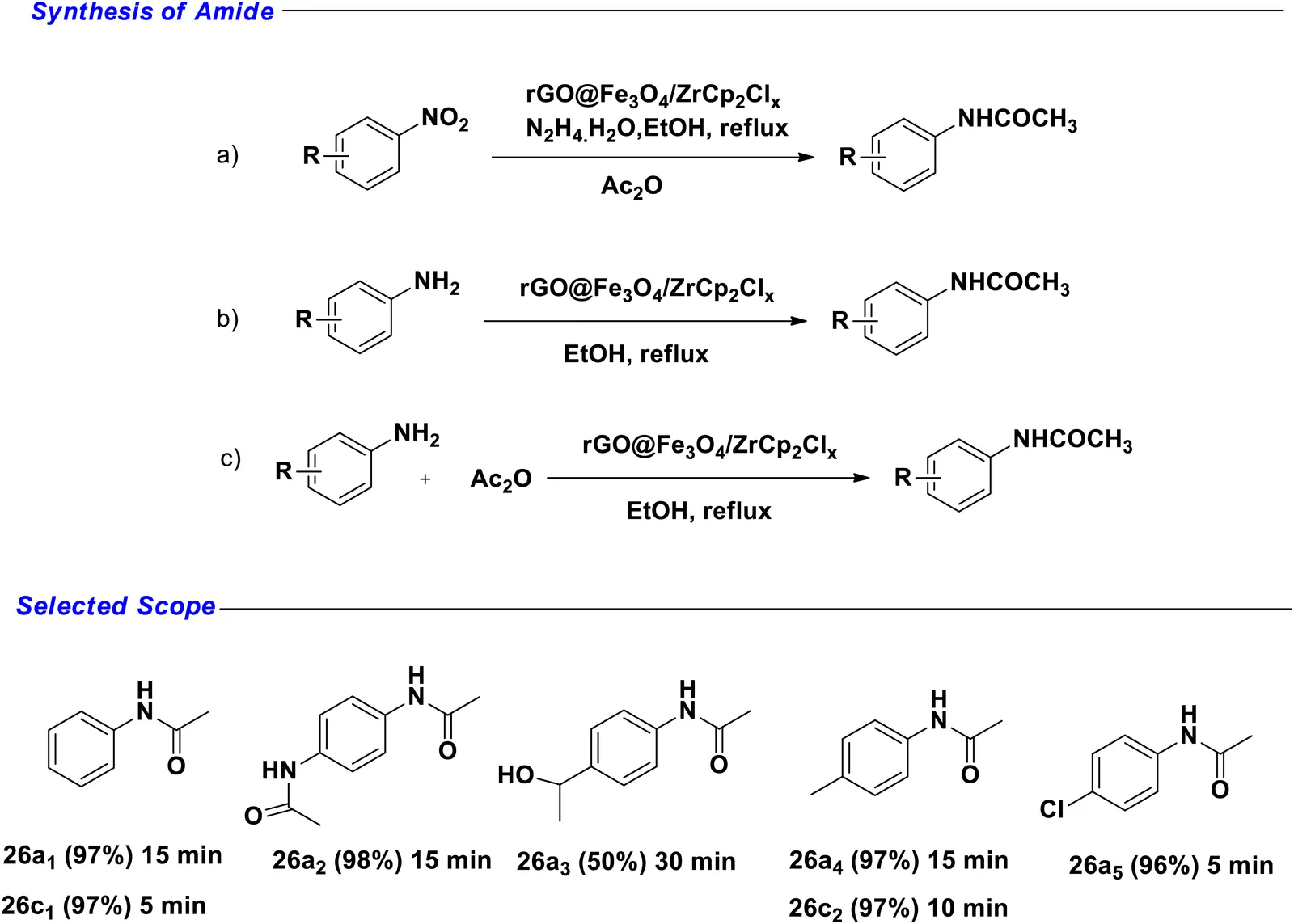
*(a) Reductive acetylation of nitroarenes (b) acetylation of arylamines using rGO@Fe*_*3*_ *O*_*4*_ */ZrCp*_*2*_ *Cl*_***x***_ **catalyst.**

Bayzidi *et al.* developed a tailored zirconocene complex on rGO@Fe_3_O_4_ as a novel magnetic nanocatalyst (rGO@Fe_3_O_4_/ZrCp_2_Cl_*x*_), where *x* = 0, 1, or 2.^70^ The author used the same catalyst for the synthesis of amides by following methods: (a) the direct amidation of aryl halides with acetamide to form *N*-arylacetamides, (b) the one-pot reductive acetylation of nitroarenes using sodium borohydride and acetic acid (c) acylation of arylamines (Scheme 26). All methods exhibited broad substrate scope, excellent yields (up to 95%), and functional group compatibility under mild conditions. Notably nanocatalyst showed high stability and was reused up to six times without significant loss of activity or zirconium leaching. The reaction was optimized with 20 mg of catalyst and refluxed in ethanol. In the reductive acetylation of nitroarenes a selectivity was noticed between hydroxy and amine groups. In the acetylation reaction, electron-releasing substituents accelerate the acetylation reaction at higher rates than electron-withdrawing groups. This work presents a green, efficient, and easily recyclable approach to C–N bond formation, offering valuable advantages for sustainable organic synthesis and potential industrial applications.

**Scheme 27:**
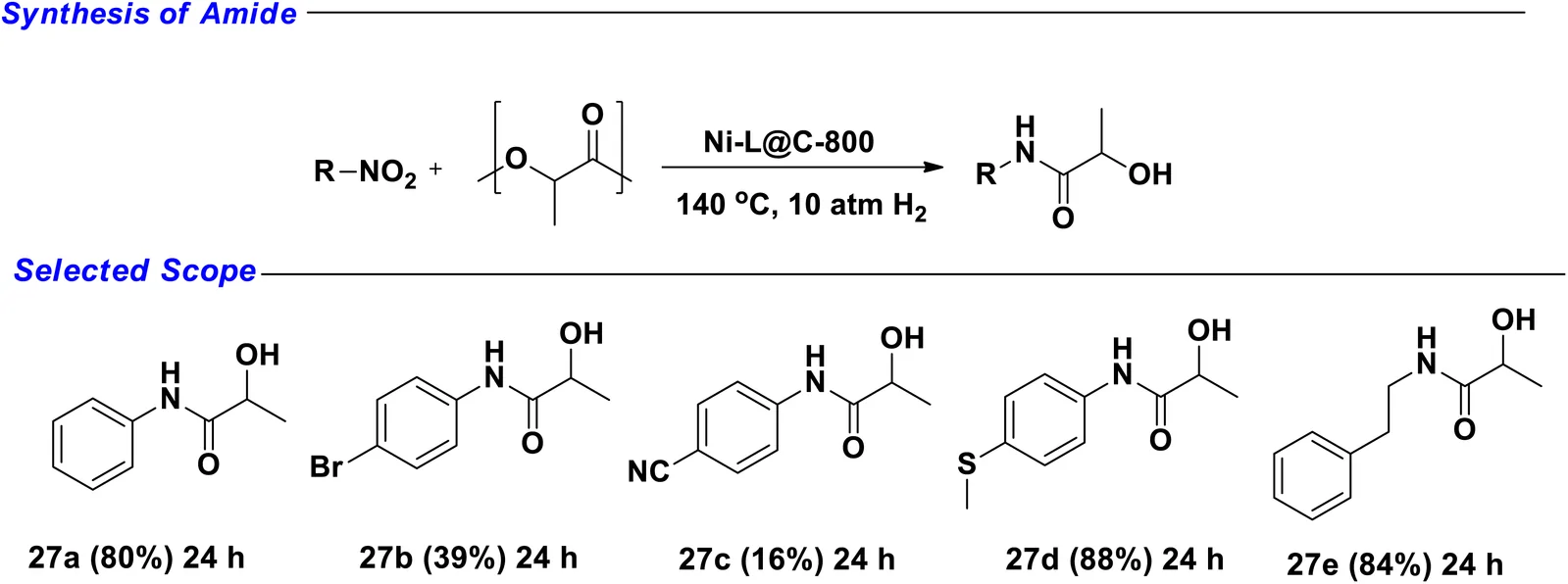
Of Ni–L@C-800 catalyzed reductive amidation of nitro compounds with polylactic acid.

A reductive amidation of nitro compounds with polylactic acid (PLA) was catalyzed by Ni-nanoparticle-based catalysts, were reported by Gao and coworkers.^71^ The catalyst was synthesized by combining Ni(NO_3_)_2_·6H_2_O and 1,2-diaminobenzene on a carbon support, followed by pyrolysis under argon (Scheme 27). These Ni-based catalyst successfully catalyzed both aromatic and aliphatic nitro compounds were transformed into their corresponding amide products (16–84%). In addition to the synthetic performance, the recycling and reusability of Ni–L@C-800 were tested. H_2_O is central to amide product formation by promoting the hydrolysis of polylactic acid to produce lactic acid monomers.

**Scheme 28:**
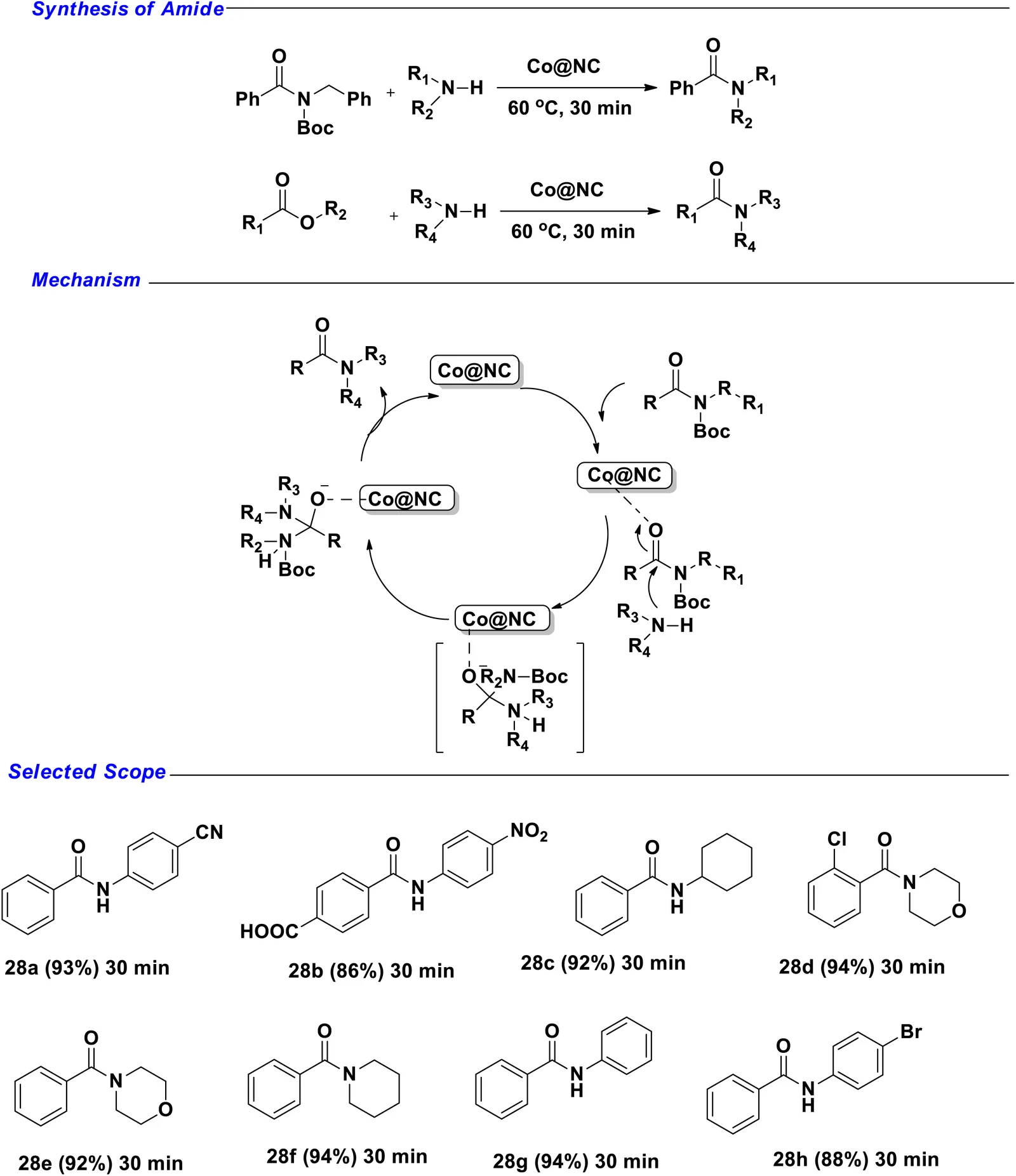
Synthesis of amides *via* transamidation method under solvent-free conditions by Co@NC catalyst.

Srivastava and group reported magnetic Co@NC nanoparticles for the synthesis of amides *via* transamidation of secondary carboxamides and amidation of esters under solvent-free conditions.^72^ In the transamidation methodology, *N*-Boc-activated amides with aryl/aliphatic amines were optimally carried out at 60 °C in the presence of 5 mg of catalyst under solvent-free conditions (Scheme 28). A variety of Boc-protected aliphatic and aromatic amides, with diverse aliphatic, aromatic, cyclic, acyclic, and sterically hindered amines, was explored. Aromatic amines, specifically aniline derivatives featuring electron-releasing groups at the *ortho*, *meta*, and *para* positions, were effectively accommodated with *tert*-butylbenzoyl(benzyl)carbamate that resulting in high to excellent yields (81–94%). They also revealed *N*-Boc *N*-benzyl benzamide with electron-releasing groups at the *ortho*, *meta*, and *para* positions (CH_3_, OCH_3_) underwent a smooth transformation, yielding the desired products in high to excellent yields. Conversely, electron-withdrawing groups (halogens, trifluoromethyl, nitro) at the *ortho*, *meta*, and *para* positions smoothly provided the transamidation products in 88–94% yields. The group also explored direct amidation using esters and amides with excellent yields of the product under the same reaction conditions. The catalyst can be recovered easily and reused several times.

### 4.12 Name reactions

**Scheme 29:**
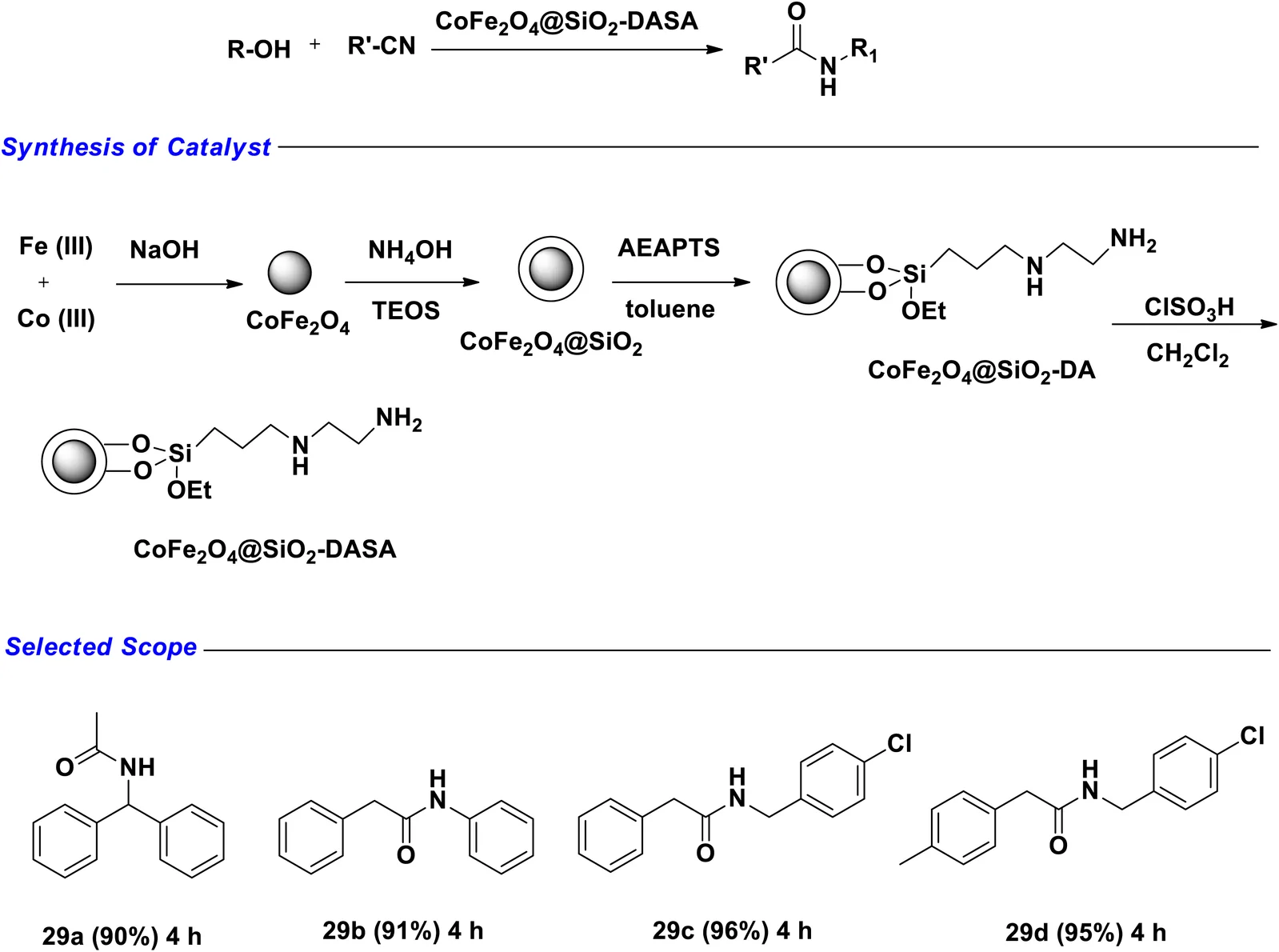
CoFe_2_O_4_@SiO_2_–DASA catalyzed synthesis of amides *via* the Ritter reaction.

Xiao-Na Zhao and group developed a magnetic CoFe_2_O_4_ nanoparticle-immobilized diamine-*N*-sulfamic acid (CoFe_2_O_4_@SiO_2_–DASA) and used it as a catalyst for the synthesis of amides *via* the Ritter reaction^73^ (Scheme 29). The synthesis of amides was explored using substituted alcohols, nitriles, and 10 mg of catalyst at 80 °C with excellent yields of products (81–96%) within 4 h. They claimed the catalyst can be recovered and reused at least 6 times.

**Scheme 30:**
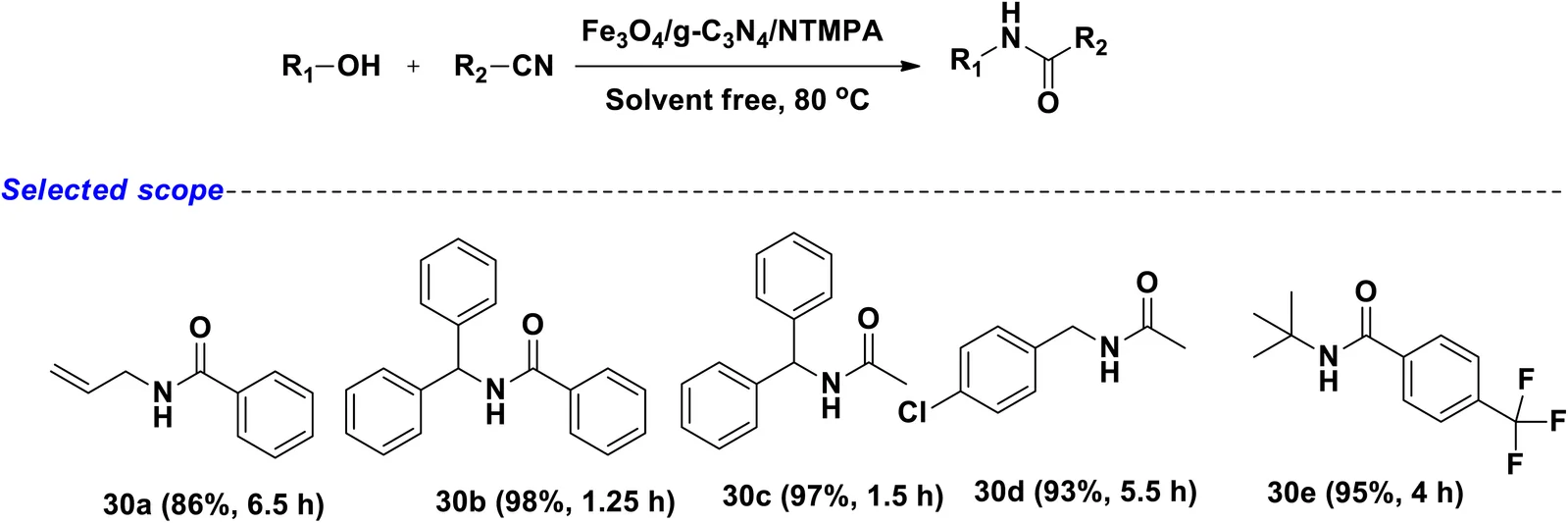
Fe_3_O_4_/g-C3N4/NTPA catalyzed synthesis of amides *via* the Ritter reaction.

Karimitabar *et al.* reported a green and efficient approach for amide synthesis *via* the Ritter reaction using a magnetically recoverable Fe_3_O_4_/g-C_3_N_4_/NTMPA nanocomposite catalyst under solvent-free conditions at 80 °C^74^ (Scheme 30). Comprehensive characterization (FT-IR, XRD, FE-SEM, TEM, DLS, EDX, BET, and TGA) confirmed successful synthesis, mesoporous structure, and good thermal stability of the catalyst. The system exhibited broad substrate scope, converting tertiary, benzylic, and primary alcohols into amides with good to excellent yields (82–98%) within 1.25–6.5 hours. Reactivity trends showed that stable carbocation-forming alcohols (tertiary and diphenylmethanol) gave faster reactions and higher yields, while electron-withdrawing groups, such as nitro, slightly reduced reactivity. Aromatic nitriles generally provided yields above 90%, with electron-withdrawing substituents enhancing reaction efficiency, whereas electron-donating groups led to slightly longer reaction times. Importantly, the catalyst retained over 90% activity after six cycles, highlighting its robustness, recyclability, and suitability for sustainable amide synthesis.

### 4.13 Miscellaneous

**Scheme 31:**
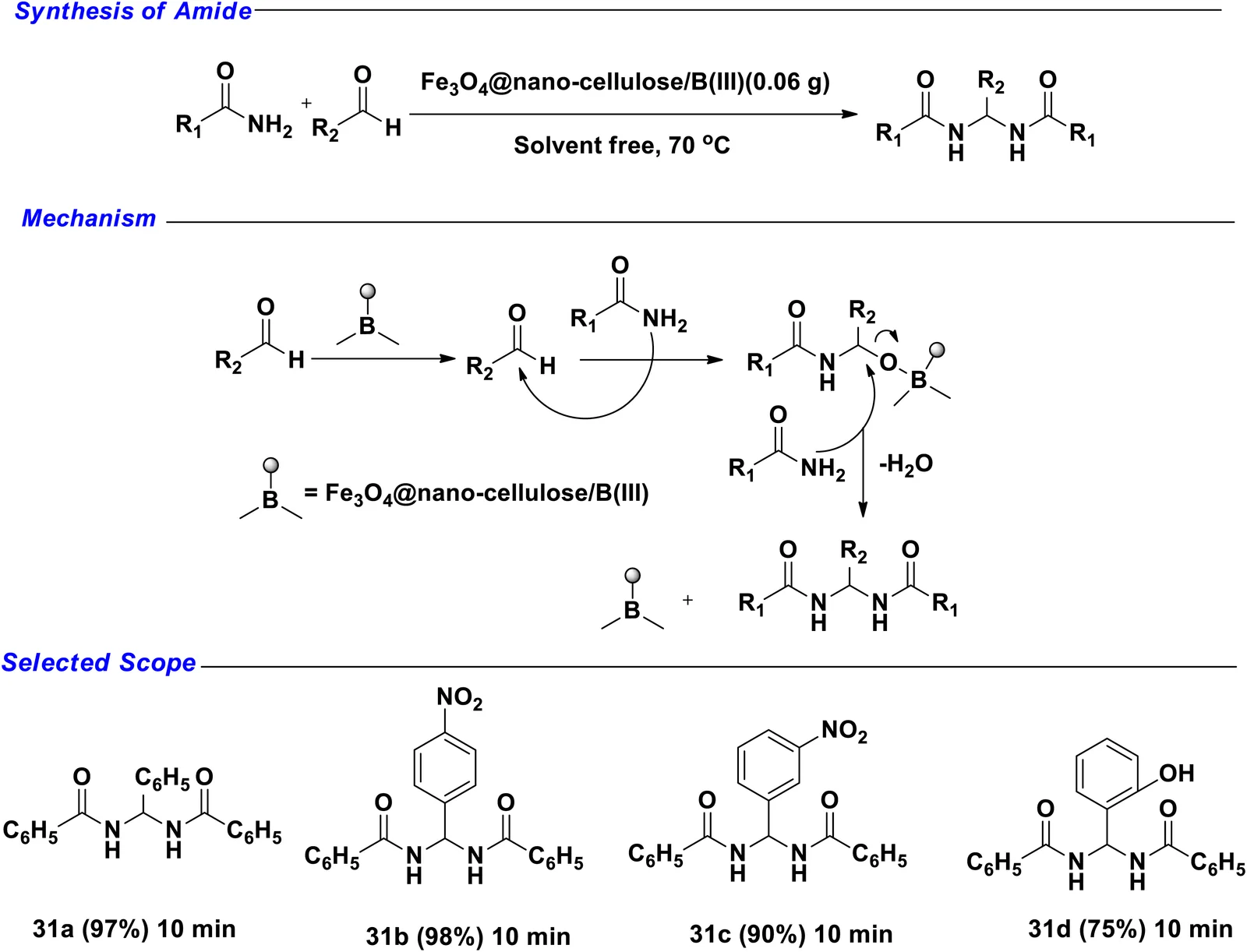
Synthesis of *N*, *N*′-alkylidenebisamides by Fe_3_O_4_@Nano-cellulose/B(iii).

Fe_3_O_4_@Nano-cellulose/B(iii) used as superparamagnetic nanocatalyst for the synthesis of *N*, *N*′-alkylidenebisamides reported by Mirjalili and group.^75^ In this methodology, the one-pot three-component condensation reaction of various aldehydes and amides was explored using 0.06 g of catalyst at 70 °C under solvent-free conditions, which provided excellent yields (75–98%) (Scheme 31). The magnetic catalyst was easily recovered and reused several times.

**Scheme 32:**
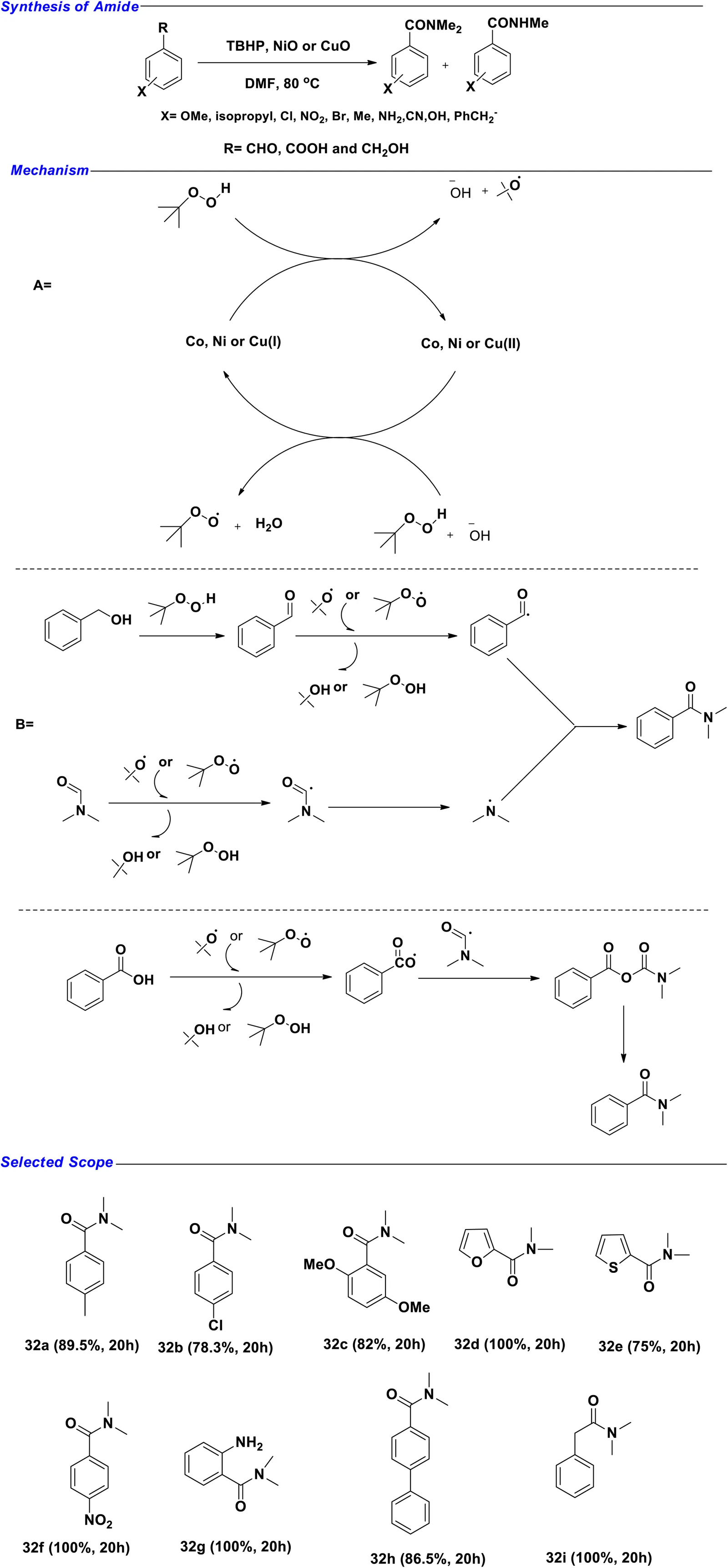
Oxidative amidation of benzyl alcohol, benzaldehyde, and benzoic acid by NiO and CuO nanocatalysts.

Mobin and co-workers reported the synthesis of NiO and CuO nanocatalysts through a sol–gel-assisted thermal decomposition approach using the complexes [Ni(hepH)(H_2_O)_4_]SO_4_ (SSMP-1) and [Cu(µ-hep)(BA)]_2_ (SSMP-2) as single-source molecular precursors (SSMPs). In these complexes, hep-H represents 2-(2-hydroxyethyl)pyridine, while BA denotes benzoic acid^76^ (Scheme 32). The prepared nanocatalysts were evaluated for the oxidative amidation of benzyl alcohol, benzaldehyde, and benzoic acid. The reactions were performed in *N*,*N*-dimethylformamide (DMF) as both the solvent and amine source, with *tert*-butyl hydroperoxide (TBHP) as the oxidant at 80 °C, which provided conversions of 14.4 to 100% in 20 h.

Among the investigated catalysts, CuO exhibited superior catalytic performance, efficiently promoting the oxidative amidation of a wide range of oxygenated and unsaturated organic substrates, demonstrating a broad substrate scope. Furthermore, the CuO nanocatalyst demonstrated excellent stability and recyclability, recovering without structural alteration and being reused for up to 6 consecutive cycles without significant loss in catalytic activity.

**Scheme 33:**
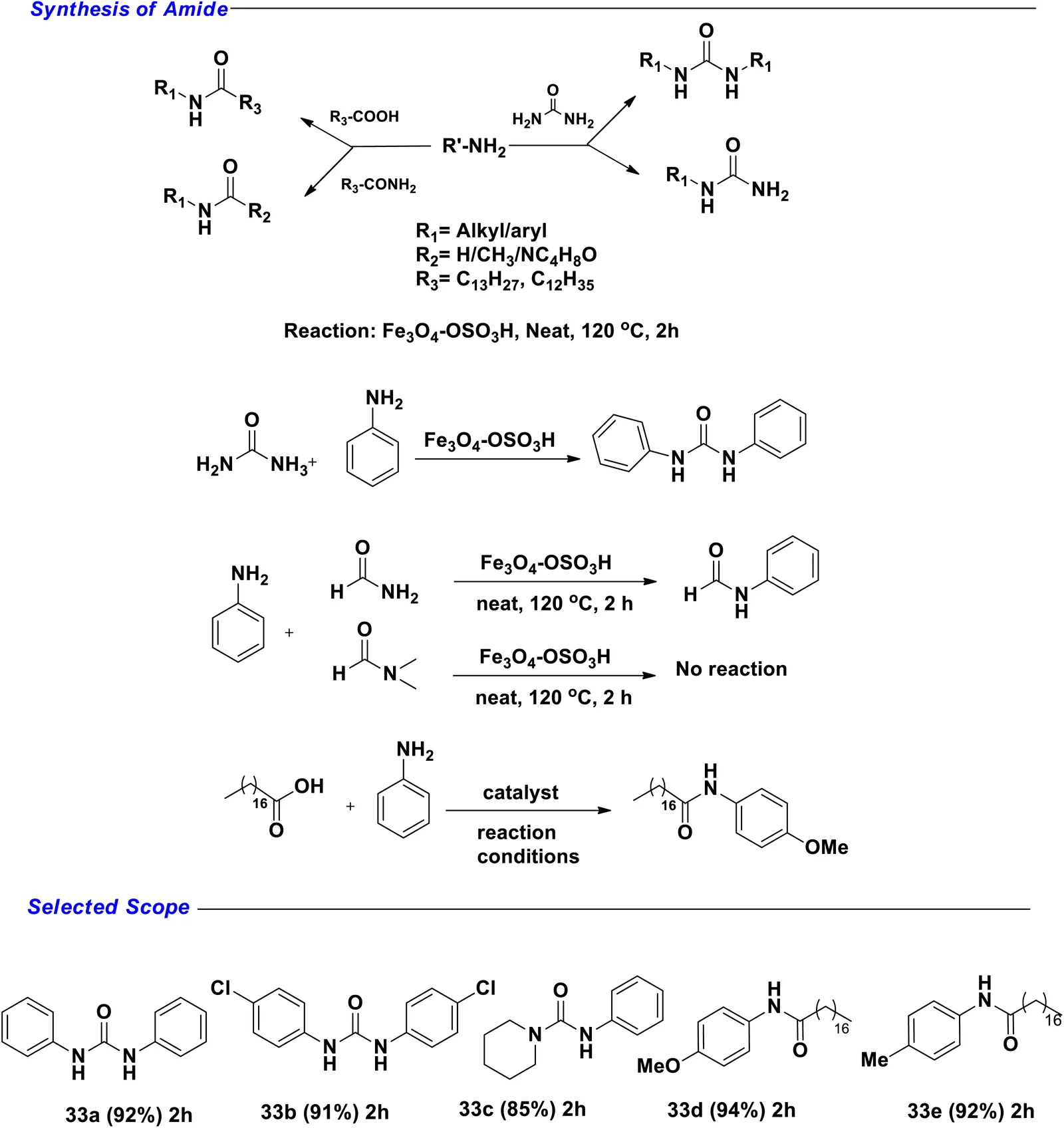
Synthesis of amides *via* transamidation reactions using Fe_3_O_4_–OSO_3_H.

Ganesan *et al.* reported the synthesis of Fe_3_O_4_–OSO_3_H and evaluated its catalytic activity for the preparation of symmetrically and unsymmetrically substituted urea derivatives (84–96%) *via* transamidation reactions^77^ (Scheme 33). The catalyst was also applied to the transamidation of cyclic and acyclic amides and the amidation of fatty acids (76–94%), showing good recyclability for up to five cycles without significant loss of activity. The reaction proceeded faster with electron-rich 4-methylaniline than with 4-chloroaniline, indicating the influence of substituent electronic effects. However, strongly electron-withdrawing nitroaniline derivatives did not afford the desired products and attempts to synthesize unsymmetrical ureas through one-pot transamidation were unsuccessful. Additionally, secondary amines such as morpholine, piperidine, and diphenylamine failed to produce the corresponding amides in fatty acid amidation reactions.

**Scheme 34:**
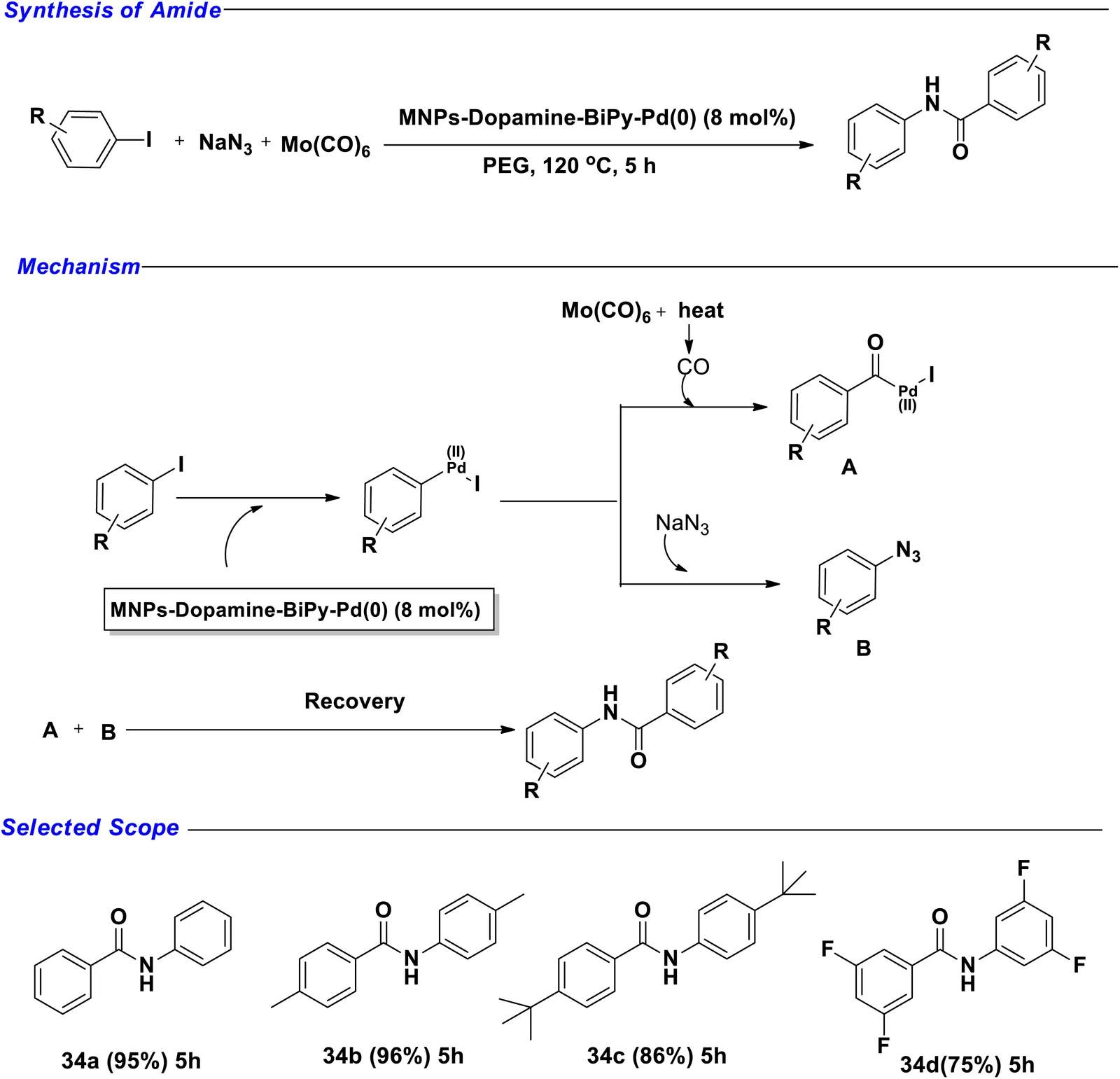
MNPs-dopamine-BiPy-Pd(0) catalyzed synthesis of amides *via* azidation/carbonylation of aryl halides.

Zheng and co-workers developed a palladium(0) complex immobilized on dopamine–bipyridine functionalized Fe_3_O_4_ magnetic nanoparticles (MNPs–dopamine–BiPy–Pd(0)) and evaluated its catalytic performance for the mild synthesis of amides *via* azidation/carbonylation of aryl halides^78^ (Scheme 34). Under the optimized reaction conditions (8 mol% catalyst, 120 °C in PEG), a broad range of aryl iodides bearing both electron-donating and electron-withdrawing substituents were efficiently converted into the corresponding amides using sodium azide as the nitrogen source and Mo(CO)_6_ as the carbonyl source, affording yields of 75–97% within 5 h. Substrate scope studies revealed that the electronic nature of the substituents had minimal influence on the reaction rate, although aryl iodides with electron-donating groups generally provided slightly higher yields than those bearing electron-withdrawing groups. Notably, the magnetically recoverable MNPs–dopamine–BiPy–Pd(0) catalyst exhibited excellent recyclability, retaining high catalytic activity over eight consecutive reaction cycles.

**Scheme 35:**
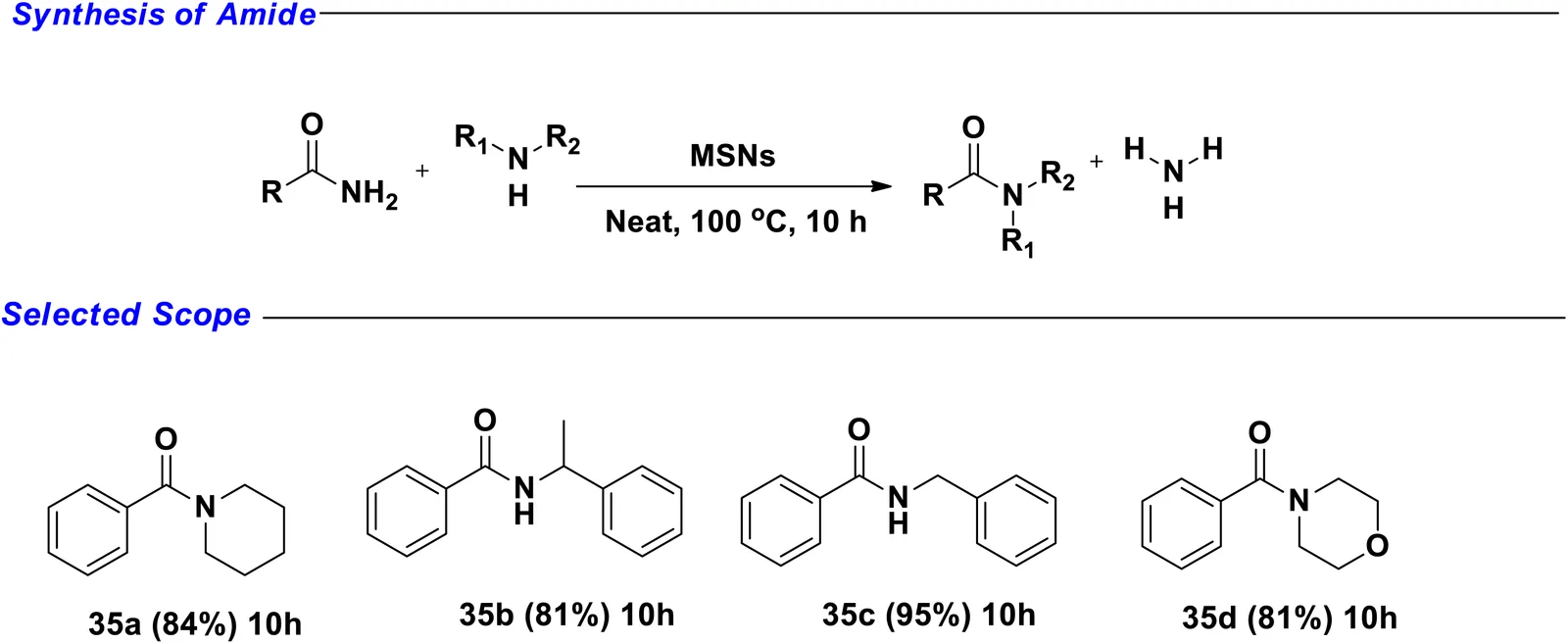
Transamidation of amides with amines by mesoporoussilica nanoparticles (MSNs).

Eidi and co-worker reported a protocol that described transamidation of amides with amines in the presence of mesoporoussilica nanoparticles (MSNs) used as a green, heterogeneous, and recyclable nanocatalyst, under solvent-free conditions.^79^ In this methodology transamidation of a variety of carboxamides or thiourea/urea with amines proceeds with the optimized reaction conditions such as 10 mg of MSNs catalyst at 100 °C under solvent free condition in 10 h (Scheme 35). Using same protocol various aromatic, aliphatic, and cyclic/acyclic amines, both primary or secondary was explored for the synthesis of different types of amides in high yields (65–96%). The transamidation reaction between benzamide and benzylamine, aliphatic, and heterocyclic amines also explored and provided good yields. The hindered amines provide lower yields compared to other types of amines. The transamidation reaction between aliphatic amides with benzylamine gives better yields compared to aromatic amines and hindered amines.

**Scheme 36:**
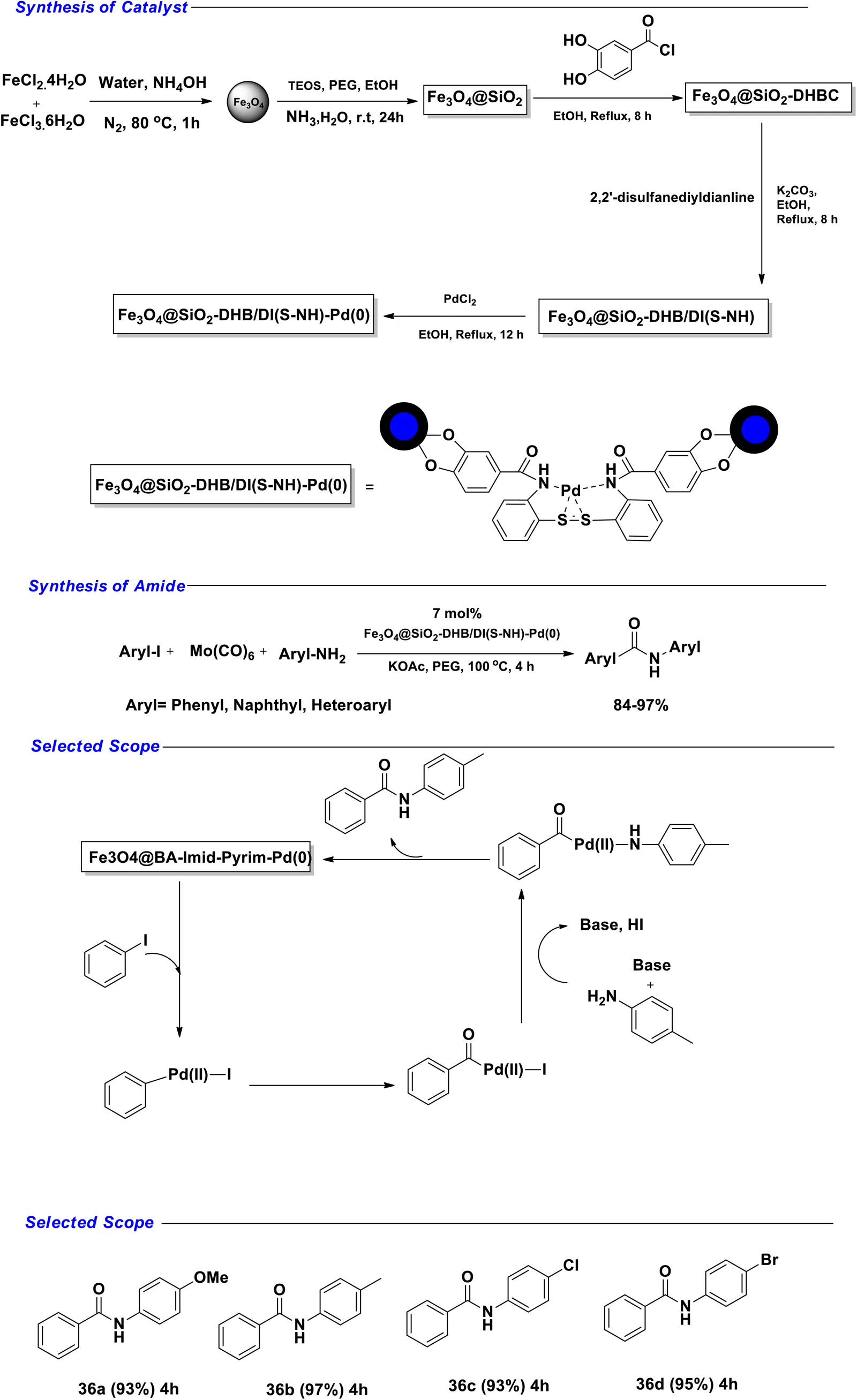
Carbonylation reaction between aryl iodides and amines catalyzed by [Fe_3_O_4_@SiO_2_-DHB/DI(S–NH)–Pd (0)].

Kazemi *et al.* reported a magnetic Fe_3_O_4_@SiO_2_–DHB/DI(S–NH)–Pd(0) catalyst for the three-component synthesis of amides *via* a carbonylation reaction between aryl iodides and amines.^80^ Under the optimized reaction conditions such as 7 mol% Fe_3_O_4_@SiO_2_–DHB/DI(S–NH)–Pd(0) catalyst, KOAc as base, PEG as solvent, at 100 °C temperature, the catalytic system efficiently promoted the formation of amides (Scheme 36). A variety of substituted aryl iodides and anilines were well tolerated, and the corresponding amide products were obtained with high efficiency (84–97%), demonstrating the broad substrate scope of the catalytic protocol.

**Scheme 37:**
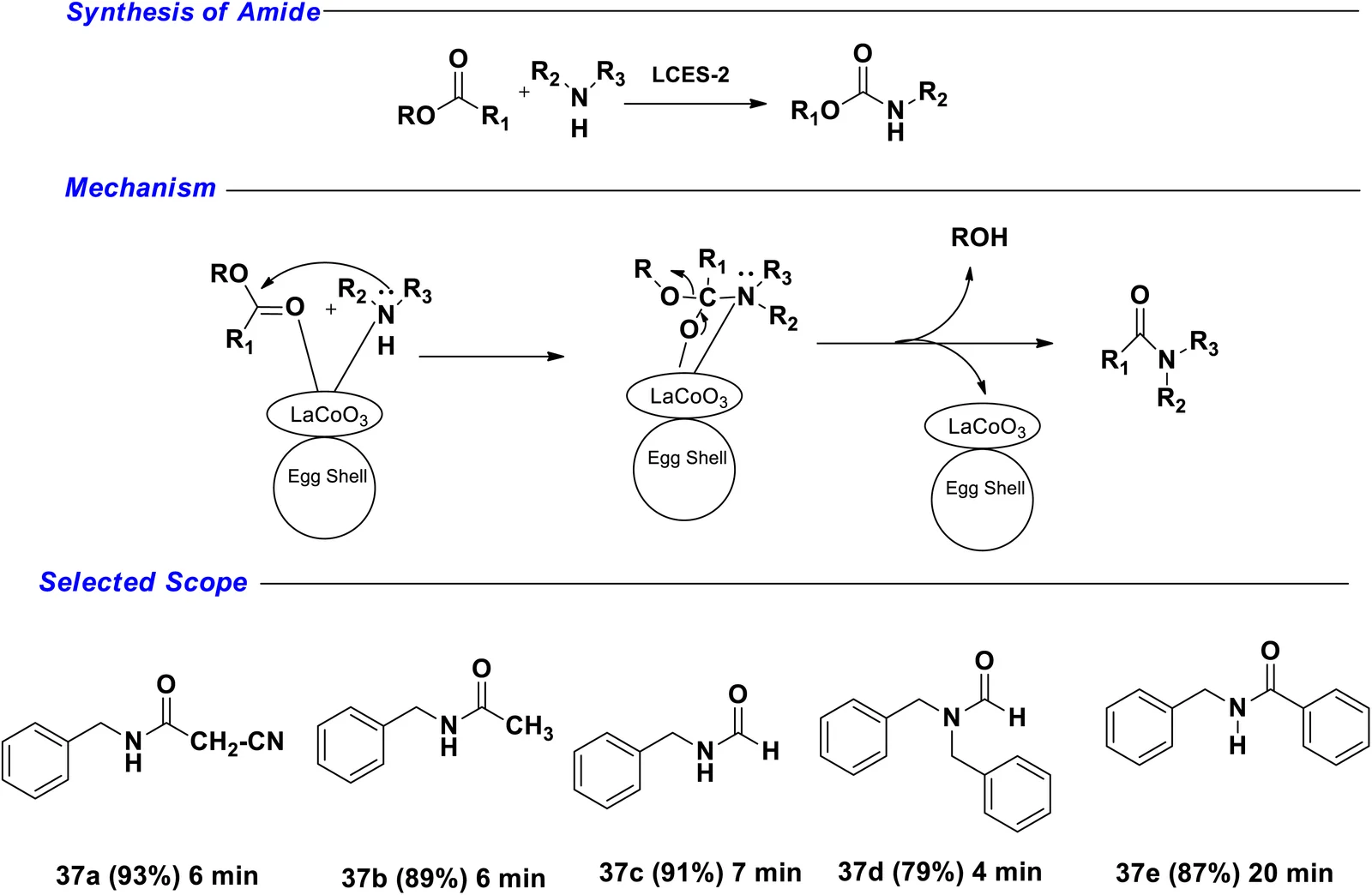
Direct amidation of esters catalyzed by LCES-2 catalyst.

Chowhan *et al.* reported a LaCoO_3_ nanorod-immobilized waste eggshell (LCES-2) catalyst as an efficient nanocatalyst for the direct amidation of esters under base- and solvent-free conditions *via* a simple grinding method.^81^ The amidation reaction between various esters and amines was carried out at room temperature using 20 mg of catalyst, affording the corresponding amides in excellent yields (78–93%) within a short reaction time (4–30 min) (Scheme 37). This protocol highlights a rapid, environmentally benign approach to amide synthesis using a recyclable heterogeneous catalyst.

**Scheme 38:**
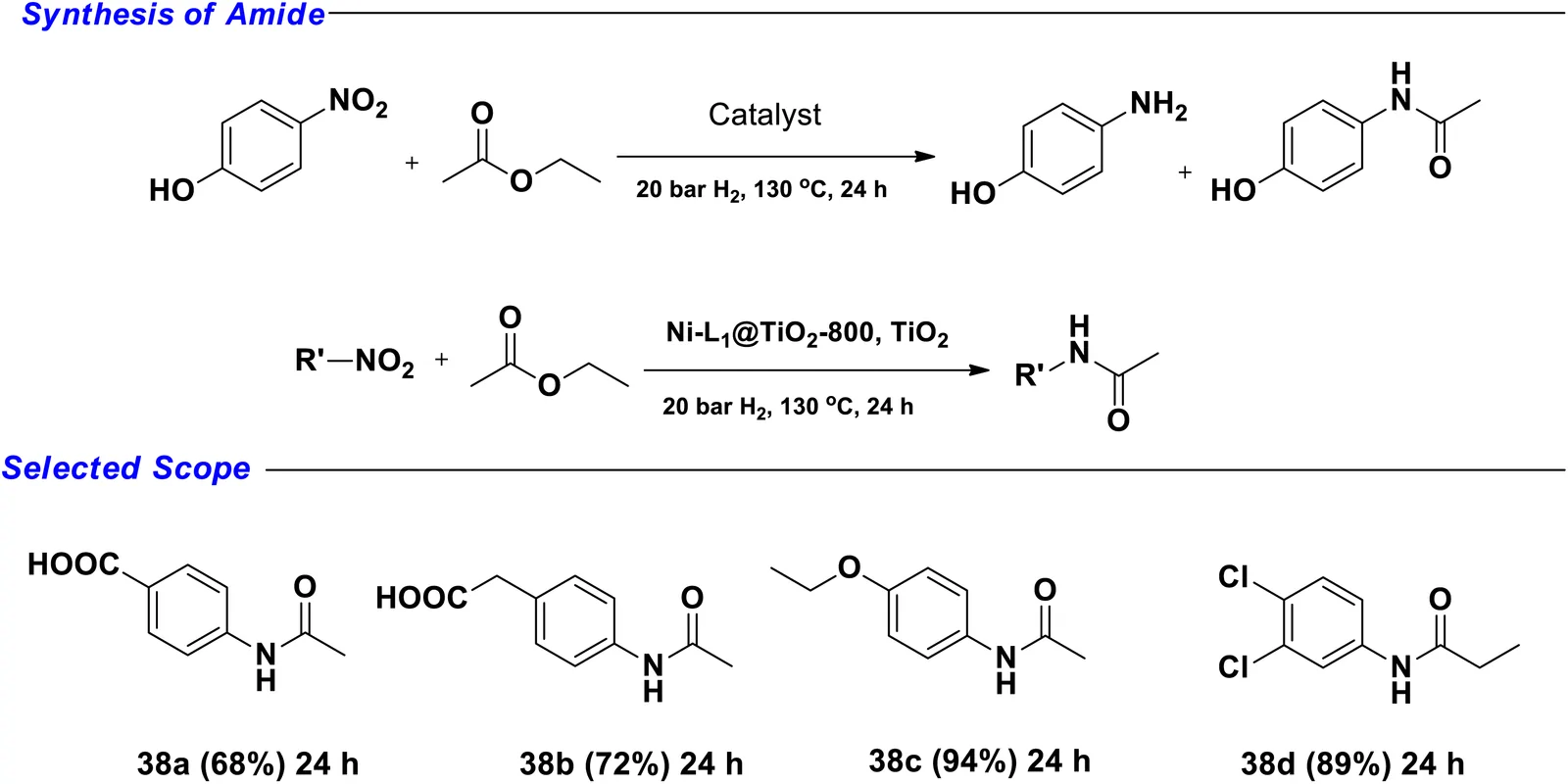
Synthesis of amides using esters with nitro compounds catalyzed by nickel-based catalyst (Ni-L1@TiO_2_-800).

Gao *et al.* reported the reductive amidation of esters with nitro compounds under additive-free conditions using a heterogeneous nickel-based catalyst (Ni-L1@TiO_2_-800).^82^ They successfully used as catalyst for the synthesis of amides using esters with nitro compounds in the presence of 30 mg of Ni-L1@TiO_2_-800 catalyst, 20 mg of TiO_2_ under 20 bar H_2_ at 130 °C within 24 h of time (Scheme 38). In this methodology, electron-rich/-poor nitroarenes, aliphatic nitro compounds, and various functional groups (halogens, ketones, esters, nitriles, vinyl, heterocycles) were well tolerated, yielding amides up to 89%. Optimization in toluene (1 : 4 nitro : ester ratio) further demonstrated the method's chemoselectivity and generality. They claimed this catalyst can be recover and reused.

**Scheme 39:**
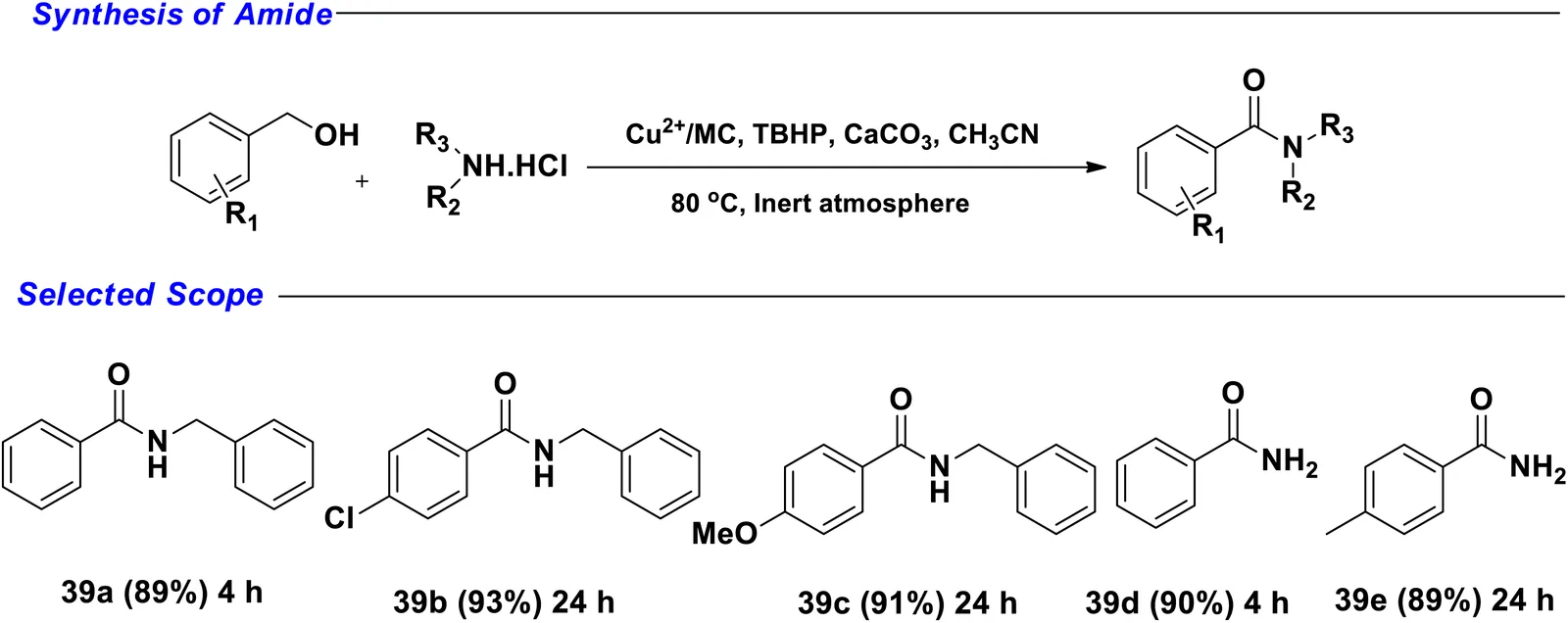
Direct amidation reaction of alcohols with amine hydrochloride salts using Cu^2+/^MC catalyst.

Ghafuri and coworkers reported a Cu^2+^/mesoporous carbon nanocatalyst (Cu^2+/^MC) for oxidative amidation reactions of alcohols.^83^ In this methodology the amidation reaction of alcohols with different amine hydrochloride salts in the presence of TBHP as oxidant, CaCO_3_ as base and acetonitrile as solvent under an inert atmosphere at 80 °C of temperature (Scheme 39). To evaluate the catalytic performance of the Cu^2+/^MC nanocatalyst, several alcohols bearing electron-drawing and electron-donating groups, along with different amine hydrochloride salts, were studied under optimized conditions, yielding high to excellent yields (80–93%). This catalyst can be recovered and reused.

**Scheme 40:**
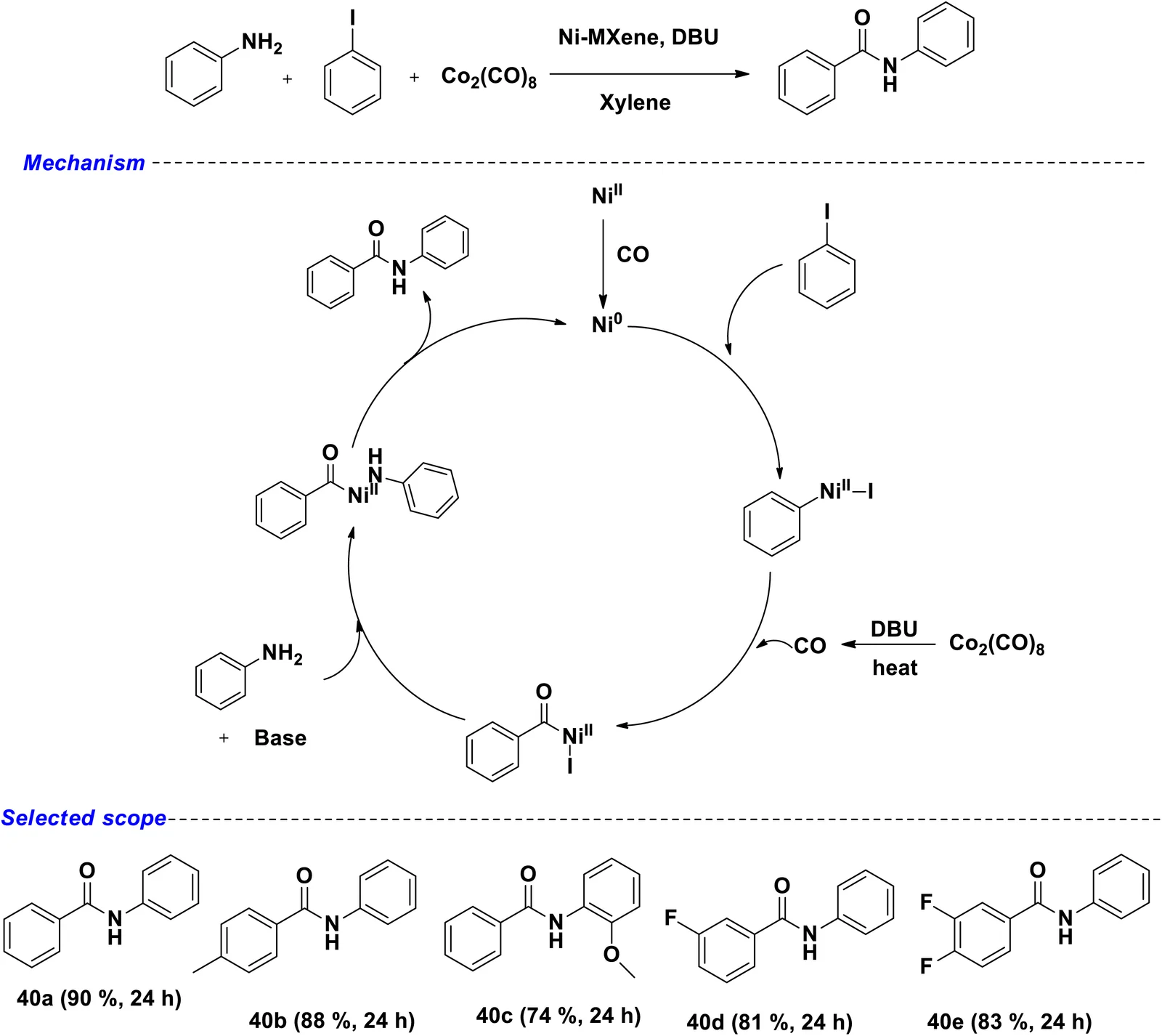
Direct amidation reaction of aryl halide with amine using Ni-Mxene catalyst.

In the year 2026, Jawale and his co-worker synthesized Ni-doped MXene and used it as a highly effective and recyclable heterogeneous catalyst for the synthesis of *N*-arylbenzamides from arene halides with arene amines, employing Co_2_(CO)_8_ as a solid CO surrogate.^84^ The reaction of arene amines (1.2 mmol), arene halides (1 mmol), under the optimized conditions such as base (3 mmol), xylene (3 mL), Ni-MXene (15 mol%), Co_2_(CO)_8_ (0.6 mmol) in a pressure tube, time (24 h) at emperature 140 °C produce *N*-arylbenzamides product *n* good to excellent yields (65–90%) (Scheme 40). The position of substituents slightly influences coupling efficiency, with *para*- and *meta*-substituted arene amines giving excellent yields, while *ortho* substitution results in slightly lower yields due to steric hindrance. Electron-donating groups (*e.g.*, methyl, ethyl, phenyl, methoxy) generally provide good to excellent yields, whereas electron-withdrawing groups, such as fluoro, also provide excellent yields due to their strong electronegativity. In contrast, chloro-substituted substrates give only moderate yields, and bromobenzene and chlorobenzene are unreactive under the optimized conditions.

**Scheme 41:**
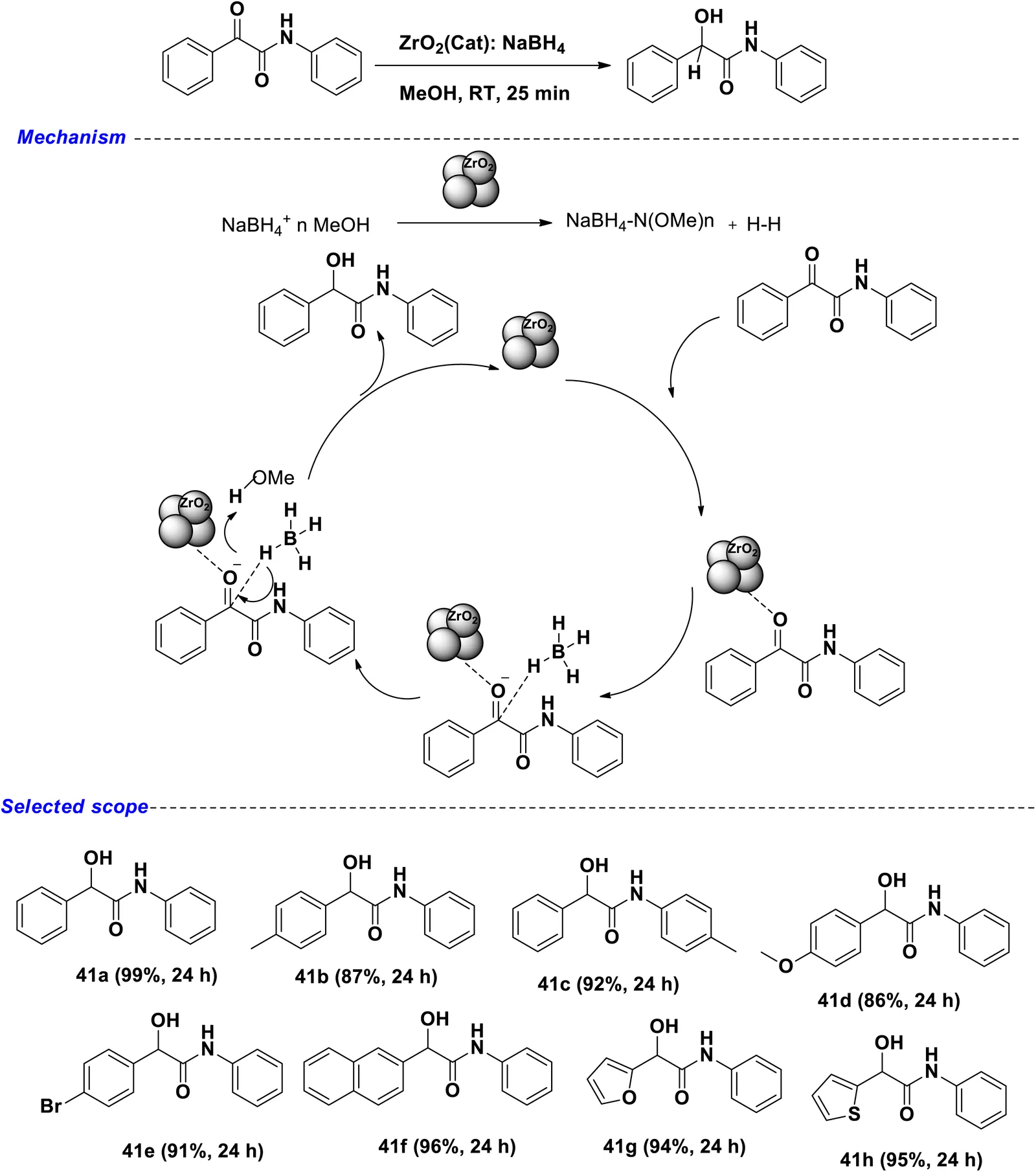
Chemoselective reduction of α-keto esters and amides using ZrO_2_ nanoparticles.

Mathkar *et al.* reported the synthesis and application of zirconia (ZrO_2_) nanoparticles as efficient catalysts for the chemoselective reduction of α-keto esters and amides^85^ (Scheme 41). Monoclinic ZrO_2_ nanoparticles showed the highest catalytic activity, achieving 83–99% yields within 20–25 minutes using NaBH_4_ and green solvents like methanol and ethanol. Optimization studies identified ethanol as the best solvent, giving up to 99% yield even at room temperature. The catalyst was essential for selectivity, as reactions without it produced undesired products. Other reducing agents were largely ineffective, confirming NaBH_4_ as optimal. The system also showed excellent tolerance toward various functional groups, including halogens and electron-donating substituents. Additionally, the catalyst demonstrated strong reusability, maintaining performance over five cycles.

**Scheme 42:**
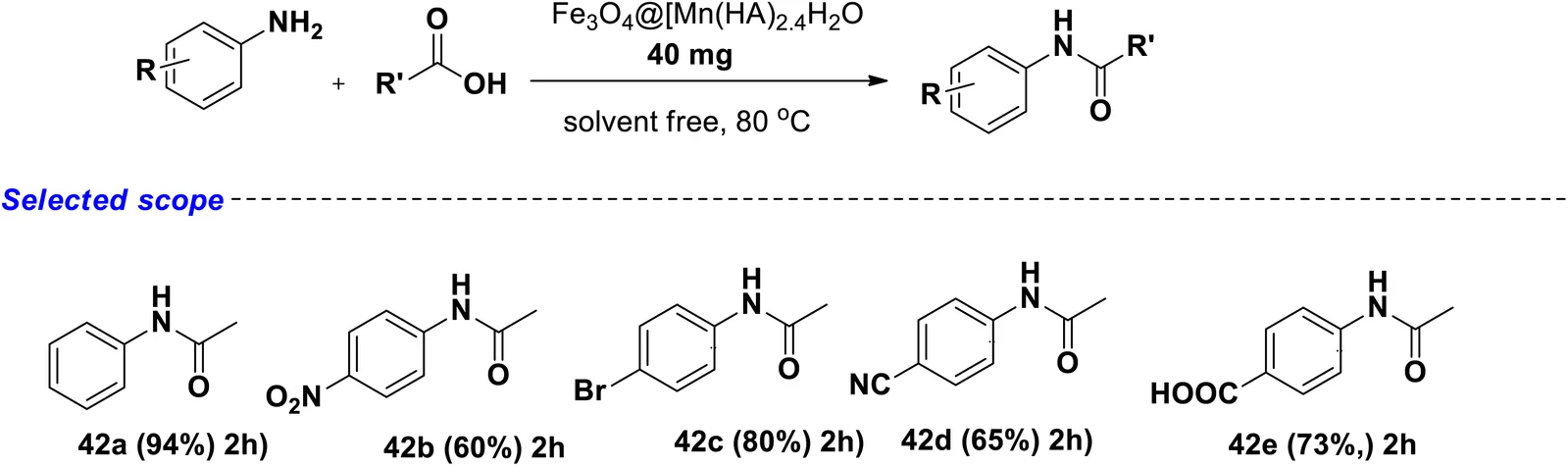
Direct and selective amidation of aliphatic carboxylic acids by Fe_3_O_4_@[Mn(HA)_2_ 4H_2_O] catalyst.

Ghassamipour *et al.* reported the direct and selective amidation of aliphatic carboxylic acids using Mn^2+^-ascorbate functionalized Fe_3_O_4_ nanoparticles (Fe_3_O_4_@[Mn(HA)_2_ 4H_2_O], 40 mg) as magnetic nano catalyst^86^ (Scheme 42). In this methodology, the catalyst demonstrated good catalytic activity (60–94% yield), facile magnetic recovery, and reusability with minimal loss of efficiency across multiple cycles. The method showed high selectivity toward aliphatic amides without requiring prior activation of carboxylic acids. Reactivity was mainly influenced by steric factors rather than electronic effects, with bulky and aromatic carboxylic acids showing poor or no conversion, likely due to reduced molecular interactions in the solid state. Additionally, reactions with *ortho*-aminophenol led to benzoxazole formation *via* intramolecular cyclization. Mechanistic studies suggested Lewis acid activation by the nanocatalyst, while comparison with existing methods highlighted its selectivity, operational simplicity, and avoidance of hazardous solvents.

## 5. Mechanistic insights

A detailed understanding of reaction mechanisms provides valuable insight into reactant activation, surface interactions, and the elementary steps involved in nanocatalyst-mediated processes. In conventional amidation strategies that rely on coupling reagents, the reaction typically proceeds through pre-activation of the carboxylic acid to form reactive intermediates such as acyl chlorides or anhydrides. In contrast, nanocatalyst-assisted systems exhibit a more diverse mechanistic landscape, often involving multiple and concurrent activation pathways.

In direct amidation reactions catalyzed by metal oxide nanomaterials such as nano-TiO_2_ or ZnO, the process generally begins with adsorption of the carboxylic acid onto the catalyst surface, where it is activated through Lewis acid–base interactions with surface metal centers.^76–82^ This interaction increases the electrophilicity of the carbonyl carbon, facilitating nucleophilic attack by the amine. Subsequent proton transfer steps promote dehydration and amide bond formation. Surface hydroxyl groups on the nanocatalyst often play an important role in stabilizing intermediates and assisting proton shuttling, thereby enabling the reaction to proceed efficiently under milder conditions.

In oxidative amidation, particularly having alcohols or aldehydes as starting materials, nanocatalysts serve as redox-active centers. In case of supported gold nanoparticles used for oxidation of benzyl alcohols to benzaldehydes followed by the formation of hemiaminal intermediates upon reaction with amines. These intermediates further oxidized by oxidant or O_2_ to form corresponding amides.^83^

Overall, mechanistic investigations of nanocatalyst catalyzed for synthesis of amides revealed the wide range of activation modes and catalytic cycles. These studies not only enhance fundamental understanding but also provide guiding principles for designing more selective, robust, and recyclable catalytic systems. Future progress will benefit from the integration of advanced spectroscopic techniques, theoretical modelling, and real-time kinetic analysis to further elucidate these complex pathways and support the development of next-generation nanocatalysts. The mechanistic schemes for the various synthetic strategies have been summarized earlier (Schemes 1–42).

## 6. Applications in the synthesis of amide derivatives

The applications of nanocatalyst for the synthesis of amide and their derivatives in various fields particularly industrial, pharmaceutical, and materials chemistry. Nanocatalysts have ability to operate under mild conditions, combined with high catalytic efficiency, selectivity, and recyclability, makes them particularly attractive for the development of sustainable synthetic protocols. In pharmaceutical chemistry, nanocatalyst-mediated amidation is widely applied in the construction of complex bioactive molecules, including peptides, β-lactams, and non-steroidal anti-inflammatory drugs (NSAIDs).^87^ Nanostructured zinc oxide and iron oxide systems have been reported to promote amide bond formation in drug intermediates with high yields and broad functional group tolerance.

Beyond pharmaceutical applications, nanocatalytic amidation strategies are also extensively employed in the synthesis of agrochemicals and advanced functional materials. In this context, selective amide bond formation plays a key role in determining the biological activity and stability of herbicides and pesticides.^88^ The use of nanocatalysts not only enhances synthetic efficiency but also helps reduce reliance on hazardous solvents and stoichiometric reagents, aligning with greener process design.

In polymer chemistry, nanocatalyst-assisted amide formation contributes to the production of high-performance polyamides such as nylon and aramids.^89^ The superior thermal and mechanical properties of these materials are closely associated with the robustness and regularity of amide linkages within the polymer backbone. In addition, the use of magnetic nanocatalysts, such as Fe_3_O_4_-based systems, enables straightforward separation and recovery after reaction, which is particularly advantageous for scalable industrial applications.

Furthermore, functionalized nanocatalysts have been applied in bioconjugation chemistry for the selective installation of amide-linked reporter groups and drug conjugates.^90^ This approach is especially important in chemical biology and diagnostic applications, where site-selective modification allows labeling without disturbing the native structure or function of biomolecules.

Overall, the integration of nanocatalysts into amide derivative synthesis not only enhances reaction efficiency and selectivity but also represents a significant step toward more sustainable and environmentally responsible chemical manufacturing.

## 7. Green chemistry aspects: environmental and economic considerations

Nanocatalysts are increasingly being used to promote more sustainable approaches to amide synthesis and its derivatives. Compared to traditional methods, which often depend on hazardous coupling reagents and produce considerable waste, nanocatalytic systems offer a cleaner alternative. By using nanomaterial many these reactions can be carried out under milder conditions, including solvent-free or aqueous media, reducing both environmental impact and operational complexity.^85^ An additional advantage lies in catalyst recovery which helps limit waste generation. Their high surface area and intrinsic activity also mean that smaller amounts of catalyst are often sufficient, improving overall efficiency and lowering costs. In some cases, these systems can be adapted to continuous-flow setups, which further enhances scalability, safety, and process control. Future development of nanocatalysts should based on earth-abundant and less toxic materials is likely to play an important role in making these processes even more practical for large-scale and sustainable applications.

## 8. Challenges and future directions

Despite considerable progress, several challenges continue to limit the broader application of nanocatalysts in amide synthesis. Achieving high selectivity, particularly with structurally complex substrates, remains difficult, as multifunctional catalytic surfaces can sometimes lead to competing side reactions. Issues related to catalyst stability and reuse also persist; problems such as particle agglomeration, surface deactivation, and metal leaching can gradually reduce catalytic performance and affect long-term sustainability. The preparation of nanocatalysts in a cost-effective and reproducible manner, especially those based on noble metals, remains a practical limitation, which has encouraged ongoing efforts toward more abundant and economical alternatives. At the same time, the environmental and toxicological implications of nanomaterials need careful evaluation across their lifecycle, highlighting the importance of designing safer and more benign catalytic systems from the outset. In addition, the use of chiral nanocatalysts for asymmetric amide synthesis is still relatively underexplored and represents a promising direction for future research. Further advances in this area are likely to benefit from the integration of nanotechnology with data-driven approaches, including machine learning and high-throughput experimentation, to enable more efficient catalyst design and optimization.

## 9. Conclusion

The use of nanocatalysts in amide synthesis has developed into a valuable approach in modern organic chemistry. Their high surface area, tunable properties, and strong catalytic performance provide clear advantages over conventional systems. In many cases, these catalysts enable reactions to proceed under milder conditions, often with reduced solvent use and lower catalyst loadings, which helps limit both cost and environmental impact. This review has highlighted the range of nanocatalyst systems reported to date and their application across different synthetic routes, including direct amidation, oxidative processes, reductive coupling, and related transformations. Mechanistic aspects and catalyst reusability have also been discussed, reflecting the practical relevance of these systems. Overall, nanocatalysis offers a promising platform for more efficient and sustainable amide bond formation, and continued developments in catalyst design and process optimization are expected to further expand its scope and applicability.

## Conflicts of interest

There are no conflicts of interest.

## Data Availability

No primary research data, software, or datasets were generated or analysed as part of this review. All information discussed in this article is derived from previously published literature, which has been appropriately cited throughout the manuscript.
